# A portrait of the Higgs boson by the CMS experiment ten years after the discovery

**DOI:** 10.1038/s41586-022-04892-x

**Published:** 2022-07-04

**Authors:** The CMS Collaboration, A. Tumasyan, W. Adam, J. W. Andrejkovic, T. Bergauer, S. Chatterjee, K. Damanakis, M. Dragicevic, A. Escalante Del Valle, P. S. Hussain, M. Jeitler, N. Krammer, L. Lechner, D. Liko, I. Mikulec, P. Paulitsch, F. M. Pitters, J. Schieck, R. Schöfbeck, D. Schwarz, S. Templ, W. Waltenberger, C.-E. Wulz, M. R. Darwish, T. Janssen, T. Kello, H. Rejeb Sfar, P. Van Mechelen, E. S. Bols, J. D’Hondt, A. De Moor, M. Delcourt, H. El Faham, S. Lowette, S. Moortgat, A. Morton, D. Müller, A. R. Sahasransu, S. Tavernier, W. Van Doninck, D. Vannerom, B. Clerbaux, G. De Lentdecker, L. Favart, J. Jaramillo, K. Lee, M. Mahdavikhorrami, I. Makarenko, A. Malara, S. Paredes, L. Pétré, N. Postiau, E. Starling, L. Thomas, M. Vanden Bemden, C. Vander Velde, P. Vanlaer, D. Dobur, J. Knolle, L. Lambrecht, G. Mestdach, M. Niedziela, C. Rendón, C. Roskas, A. Samalan, K. Skovpen, M. Tytgat, N. Van Den Bossche, B. Vermassen, L. Wezenbeek, A. Benecke, G. Bruno, F. Bury, C. Caputo, P. David, C. Delaere, I. S. Donertas, A. Giammanco, K. Jaffel, Sa. Jain, V. Lemaitre, K. Mondal, J. Prisciandaro, A. Taliercio, T. T. Tran, P. Vischia, S. Wertz, G. A. Alves, E. Coelho, C. Hensel, A. Moraes, P. Rebello Teles, W. L. Aldá Júnior, M. Alves Gallo Pereira, M. Barroso Ferreira Filho, H. Brandao Malbouisson, W. Carvalho, J. Chinellato, E. M. Da Costa, G. G. Da Silveira, D. De Jesus Damiao, V. Dos Santos Sousa, S. Fonseca De Souza, J. Martins, C. Mora Herrera, K. Mota Amarilo, L. Mundim, H. Nogima, A. Santoro, S. M. Silva Do Amaral, A. Sznajder, M. Thiel, F. Torres Da Silva De Araujo, A. Vilela Pereira, C. A. Bernardes, L. Calligaris, E. M. Gregores, P. G. Mercadante, S. F. Novaes, Sandra S. Padula, T. R. Fernandez Perez Tomei, A. Aleksandrov, G. Antchev, R. Hadjiiska, P. Iaydjiev, M. Misheva, M. Rodozov, M. Shopova, G. Sultanov, A. Dimitrov, T. Ivanov, L. Litov, B. Pavlov, P. Petkov, A. Petrov, E. Shumka, T. Cheng, T. Javaid, M. Mittal, L. Yuan, M. Ahmad, G. Bauer, Z. Hu, S. Lezki, K. Yi, G. M. Chen, H. S. Chen, M. Chen, F. Iemmi, C. H. Jiang, A. Kapoor, H. Liao, Z.-A. Liu, V. Milosevic, F. Monti, R. Sharma, J. Tao, J. Thomas-Wilsker, J. Wang, H. Zhang, J. Zhao, A. Agapitos, Y. An, Y. Ban, C. Chen, A. Levin, C. Li, Q. Li, X. Lyu, Y. Mao, S. J. Qian, X. Sun, D. Wang, J. Xiao, H. Yang, J. Li, M. Lu, Z. You, X. Gao, D. Leggat, H. Okawa, Y. Zhang, Z. Lin, C. Lu, M. Xiao, C. Avila, D. A. Barbosa Trujillo, A. Cabrera, C. Florez, J. Fraga, J. Mejia Guisao, F. Ramirez, M. Rodriguez, J. D. Ruiz Alvarez, D. Giljanovic, N. Godinovic, D. Lelas, I. Puljak, Z. Antunovic, M. Kovac, T. Sculac, V. Brigljevic, B. K. Chitroda, D. Ferencek, D. Majumder, M. Roguljic, A. Starodumov, T. Susa, A. Attikis, K. Christoforou, G. Kole, M. Kolosova, S. Konstantinou, J. Mousa, C. Nicolaou, F. Ptochos, P. A. Razis, H. Rykaczewski, H. Saka, M. Finger, M. Finger, A. Kveton, E. Ayala, E. Carrera Jarrin, A. A. Abdelalim, E. Salama, M. A. Mahmoud, Y. Mohammed, S. Bhowmik, R. K. Dewanjee, K. Ehataht, M. Kadastik, S. Nandan, C. Nielsen, J. Pata, M. Raidal, L. Tani, C. Veelken, P. Eerola, H. Kirschenmann, K. Osterberg, M. Voutilainen, S. Bharthuar, E. Brücken, F. Garcia, J. Havukainen, M. S. Kim, R. Kinnunen, T. Lampén, K. Lassila-Perini, S. Lehti, T. Lindén, M. Lotti, L. Martikainen, M. Myllymäki, J. Ott, M. M. Rantanen, H. Siikonen, E. Tuominen, J. Tuominiemi, P. Luukka, H. Petrow, T. Tuuva, C. Amendola, M. Besancon, F. Couderc, M. Dejardin, D. Denegri, J. L. Faure, F. Ferri, S. Ganjour, P. Gras, G. Hamel de Monchenault, P. Jarry, V. Lohezic, J. Malcles, J. Rander, A. Rosowsky, M. Ö. Sahin, A. Savoy-Navarro, P. Simkina, M. Titov, C. Baldenegro Barrera, F. Beaudette, A. Buchot Perraguin, P. Busson, A. Cappati, C. Charlot, O. Davignon, B. Diab, G. Falmagne, B. A. Fontana Santos Alves, S. Ghosh, R. Granier de Cassagnac, A. Hakimi, B. Harikrishnan, J. Motta, M. Nguyen, C. Ochando, L. Portales, J. Rembser, R. Salerno, U. Sarkar, J. B. Sauvan, Y. Sirois, A. Tarabini, E. Vernazza, A. Zabi, A. Zghiche, J.-L. Agram, J. Andrea, D. Apparu, D. Bloch, G. Bourgatte, J.-M. Brom, E. C. Chabert, C. Collard, D. Darej, U. Goerlach, C. Grimault, A.-C. Le Bihan, P. Van Hove, S. Beauceron, C. Bernet, G. Boudoul, C. Camen, A. Carle, N. Chanon, J. Choi, D. Contardo, P. Depasse, C. Dozen, H. El Mamouni, J. Fay, S. Gascon, M. Gouzevitch, G. Grenier, B. Ille, I. B. Laktineh, M. Lethuillier, L. Mirabito, S. Perries, K. Shchablo, V. Sordini, L. Torterotot, M. Vander Donckt, P. Verdier, S. Viret, I. Lomidze, T. Toriashvili, Z. Tsamalaidze, V. Botta, L. Feld, K. Klein, M. Lipinski, D. Meuser, A. Pauls, N. Röwert, M. Teroerde, S. Diekmann, A. Dodonova, N. Eich, D. Eliseev, M. Erdmann, P. Fackeldey, B. Fischer, T. Hebbeker, K. Hoepfner, F. Ivone, M. Y. Lee, L. Mastrolorenzo, M. Merschmeyer, A. Meyer, S. Mondal, S. Mukherjee, D. Noll, A. Novak, F. Nowotny, A. Pozdnyakov, Y. Rath, W. Redjeb, H. Reithler, A. Schmidt, S. C. Schuler, A. Sharma, L. Vigilante, S. Wiedenbeck, S. Zaleski, C. Dziwok, G. Flügge, W. Haj Ahmad, O. Hlushchenko, T. Kress, A. Nowack, O. Pooth, A. Stahl, T. Ziemons, A. Zotz, H. Aarup Petersen, M. Aldaya Martin, P. Asmuss, S. Baxter, M. Bayatmakou, O. Behnke, A. Bermúdez Martínez, S. Bhattacharya, A. A. Bin Anuar, F. Blekman, K. Borras, D. Brunner, A. Campbell, A. Cardini, C. Cheng, F. Colombina, S. Consuegra Rodríguez, G. Correia Silva, M. De Silva, L. Didukh, G. Eckerlin, D. Eckstein, L. I. Estevez Banos, O. Filatov, E. Gallo, A. Geiser, A. Giraldi, G. Greau, A. Grohsjean, V. Guglielmi, M. Guthoff, A. Jafari, N. Z. Jomhari, B. Kaech, M. Kasemann, H. Kaveh, C. Kleinwort, R. Kogler, M. Komm, D. Krücker, W. Lange, D. Leyva Pernia, K. Lipka, W. Lohmann, R. Mankel, I.-A. Melzer-Pellmann, M. Mendizabal Morentin, J. Metwally, A. B. Meyer, G. Milella, A. Kasem, M. Mormile, A. Mussgiller, A. Nürnberg, Y. Otarid, D. Pérez Adán, A. Raspereza, B. Ribeiro Lopes, J. Rübenach, A. Saggio, A. Saibel, M. Savitskyi, M. Scham, V. Scheurer, S. Schnake, P. Schütze, C. Schwanenberger, M. Shchedrolosiev, R. E. Sosa Ricardo, D. Stafford, N. Tonon, M. Van De Klundert, F. Vazzoler, A. Ventura Barroso, R. Walsh, D. Walter, Q. Wang, Y. Wen, K. Wichmann, L. Wiens, C. Wissing, S. Wuchterl, Y. Yang, A. Zimermmane Castro Santos, R. Aggleton, A. Albrecht, S. Albrecht, M. Antonello, S. Bein, L. Benato, M. Bonanomi, P. Connor, K. De Leo, M. Eich, K. El Morabit, F. Feindt, A. Fröhlich, C. Garbers, E. Garutti, M. Hajheidari, J. Haller, A. Hinzmann, H. R. Jabusch, G. Kasieczka, R. Klanner, W. Korcari, T. Kramer, V. Kutzner, J. Lange, T. Lange, A. Lobanov, C. Matthies, A. Mehta, L. Moureaux, M. Mrowietz, A. Nigamova, Y. Nissan, A. Paasch, K. J. Pena Rodriguez, M. Rieger, O. Rieger, P. Schleper, M. Schröder, J. Schwandt, H. Stadie, G. Steinbrück, A. Tews, M. Wolf, J. Bechtel, S. Brommer, M. Burkart, E. Butz, R. Caspart, T. Chwalek, A. Dierlamm, A. Droll, N. Faltermann, M. Giffels, J. O. Gosewisch, A. Gottmann, F. Hartmann, C. Heidecker, M. Horzela, U. Husemann, P. Keicher, M. Klute, R. Koppenhöfer, S. Maier, S. Mitra, Th. Müller, M. Neukum, G. Quast, K. Rabbertz, J. Rauser, D. Savoiu, M. Schnepf, D. Seith, I. Shvetsov, H. J. Simonis, N. Trevisani, R. Ulrich, J. van der Linden, R. F. Von Cube, M. Wassmer, M. Weber, S. Wieland, R. Wolf, S. Wozniewski, S. Wunsch, G. Anagnostou, P. Assiouras, G. Daskalakis, A. Kyriakis, A. Stakia, M. Diamantopoulou, D. Karasavvas, P. Kontaxakis, A. Manousakis-Katsikakis, A. Panagiotou, I. Papavergou, N. Saoulidou, K. Theofilatos, E. Tziaferi, K. Vellidis, E. Vourliotis, G. Bakas, T. Chatzistavrou, K. Kousouris, I. Papakrivopoulos, G. Tsipolitis, A. Zacharopoulou, K. Adamidis, I. Bestintzanos, I. Evangelou, C. Foudas, P. Gianneios, C. Kamtsikis, P. Katsoulis, P. Kokkas, P. G. Kosmoglou Kioseoglou, N. Manthos, I. Papadopoulos, J. Strologas, M. Csanád, K. Farkas, M. M. A. Gadallah, S. Lökös, P. Major, K. Mandal, G. Pásztor, A. J. Rádl, O. Surányi, G. I. Veres, M. Bartók, G. Bencze, C. Hajdu, D. Horvath, F. Sikler, V. Veszpremi, N. Beni, S. Czellar, D. Fasanella, J. Karancsi, J. Molnar, Z. Szillasi, D. Teyssier, B. Ujvari, G. Zilizi, T. Csorgo, F. Nemes, T. Novak, J. Babbar, S. Bansal, S. B. Beri, V. Bhatnagar, G. Chaudhary, S. Chauhan, N. Dhingra, R. Gupta, A. Kaur, A. K. Virdi, H. Kaur, M. Kaur, S. Kumar, P. Kumari, M. Meena, K. Sandeep, T. Sheokand, J. B. Singh, A. Singla, A. Ahmed, A. Bhardwaj, B. C. Choudhary, M. Gola, S. Keshri, A. Kumar, M. Naimuddin, P. Priyanka, K. Ranjan, S. Saumya, A. Shah, S. Baradia, S. Barman, S. Bhattacharya, D. Bhowmik, S. Dutta, B. Gomber, M. Maity, P. Palit, P. K. Rout, G. Saha, B. Sahu, S. Sarkar, P. K. Behera, S. C. Behera, P. Kalbhor, J. R. Komaragiri, D. Kumar, A. Muhammad, L. Panwar, R. Pradhan, P. R. Pujahari, A. Sharma, A. K. Sikdar, P. C. Tiwari, S. Verma, K. Naskar, T. Aziz, I. Das, S. Dugad, M. Kumar, G. B. Mohanty, P. Suryadevara, S. Banerjee, R. Chudasama, M. Guchait, S. Karmakar, S. Kumar, G. Majumder, K. Mazumdar, S. Mukherjee, A. Thachayath, S. Bahinipati, A. K. Das, C. Kar, P. Mal, T. Mishra, V. K. Muraleedharan Nair Bindhu, A. Nayak, P. Saha, N. Sur, S. K. Swain, D. Vats, A. Alpana, S. Dube, B. Kansal, A. Laha, S. Pandey, A. Rastogi, S. Sharma, H. Bakhshiansohi, E. Khazaie, M. Zeinali, S. Chenarani, S. M. Etesami, M. Khakzad, M. Mohammadi Najafabadi, M. Felcini, M. Grunewald, M. Abbrescia, R. Aly, C. Aruta, A. Colaleo, D. Creanza, N. De Filippis, M. De Palma, A. Di Florio, W. Elmetenawee, F. Errico, L. Fiore, G. Iaselli, M. Ince, G. Maggi, M. Maggi, I. Margjeka, V. Mastrapasqua, S. My, S. Nuzzo, A. Pellecchia, A. Pompili, G. Pugliese, R. Radogna, D. Ramos, A. Ranieri, G. Selvaggi, L. Silvestris, F. M. Simone, Ü. Sözbilir, A. Stamerra, R. Venditti, P. Verwilligen, A. Zaza, G. Abbiendi, C. Battilana, D. Bonacorsi, L. Borgonovi, L. Brigliadori, R. Campanini, P. Capiluppi, A. Castro, F. R. Cavallo, M. Cuffiani, G. M. Dallavalle, T. Diotalevi, F. Fabbri, A. Fanfani, P. Giacomelli, L. Giommi, C. Grandi, L. Guiducci, S. Lo Meo, L. Lunerti, S. Marcellini, G. Masetti, F. L. Navarria, A. Perrotta, F. Primavera, A. M. Rossi, T. Rovelli, G. P. Siroli, S. Costa, A. Di Mattia, R. Potenza, A. Tricomi, C. Tuve, G. Barbagli, B. Camaiani, A. Cassese, R. Ceccarelli, V. Ciulli, C. Civinini, R. D’Alessandro, E. Focardi, G. Latino, P. Lenzi, M. Lizzo, M. Meschini, S. Paoletti, R. Seidita, G. Sguazzoni, L. Viliani, L. Benussi, S. Bianco, S. Meola, D. Piccolo, M. Bozzo, F. Ferro, R. Mulargia, E. Robutti, S. Tosi, A. Benaglia, G. Boldrini, F. Brivio, F. Cetorelli, F. De Guio, M. E. Dinardo, P. Dini, S. Gennai, A. Ghezzi, P. Govoni, L. Guzzi, M. T. Lucchini, M. Malberti, S. Malvezzi, A. Massironi, D. Menasce, L. Moroni, M. Paganoni, D. Pedrini, B. S. Pinolini, S. Ragazzi, N. Redaelli, T. Tabarelli de Fatis, D. Zuolo, S. Buontempo, F. Carnevali, N. Cavallo, A. De Iorio, F. Fabozzi, A. O. M. Iorio, L. Lista, P. Paolucci, B. Rossi, C. Sciacca, P. Azzi, N. Bacchetta, D. Bisello, P. Bortignon, A. Bragagnolo, R. Carlin, P. Checchia, T. Dorigo, F. Gasparini, U. Gasparini, G. Grosso, L. Layer, E. Lusiani, M. Margoni, A. T. Meneguzzo, J. Pazzini, P. Ronchese, R. Rossin, F. Simonetto, G. Strong, M. Tosi, H. Yarar, M. Zanetti, P. Zotto, A. Zucchetta, G. Zumerle, C. Aimè, A. Braghieri, S. Calzaferri, D. Fiorina, P. Montagna, V. Re, C. Riccardi, P. Salvini, I. Vai, P. Vitulo, P. Asenov, G. M. Bilei, D. Ciangottini, L. Fanò, M. Magherini, G. Mantovani, V. Mariani, M. Menichelli, F. Moscatelli, A. Piccinelli, M. Presilla, A. Rossi, A. Santocchia, D. Spiga, T. Tedeschi, P. Azzurri, G. Bagliesi, V. Bertacchi, R. Bhattacharya, L. Bianchini, T. Boccali, E. Bossini, D. Bruschini, R. Castaldi, M. A. Ciocci, V. D’Amante, R. Dell’Orso, M. R. Di Domenico, S. Donato, A. Giassi, F. Ligabue, E. Manca, G. Mandorli, D. Matos Figueiredo, A. Messineo, M. Musich, F. Palla, S. Parolia, G. Ramirez-Sanchez, A. Rizzi, G. Rolandi, S. Roy Chowdhury, A. Scribano, N. Shafiei, P. Spagnolo, R. Tenchini, G. Tonelli, N. Turini, A. Venturi, P. G. Verdini, P. Barria, M. Campana, F. Cavallari, D. Del Re, E. Di Marco, M. Diemoz, E. Longo, P. Meridiani, G. Organtini, F. Pandolfi, R. Paramatti, C. Quaranta, S. Rahatlou, C. Rovelli, F. Santanastasio, L. Soffi, R. Tramontano, N. Amapane, R. Arcidiacono, S. Argiro, M. Arneodo, N. Bartosik, R. Bellan, A. Bellora, J. Berenguer Antequera, C. Biino, N. Cartiglia, M. Costa, R. Covarelli, N. Demaria, M. Grippo, B. Kiani, F. Legger, C. Mariotti, S. Maselli, A. Mecca, E. Migliore, E. Monteil, M. Monteno, M. M. Obertino, G. Ortona, L. Pacher, N. Pastrone, M. Pelliccioni, M. Ruspa, K. Shchelina, F. Siviero, V. Sola, A. Solano, D. Soldi, A. Staiano, M. Tornago, D. Trocino, G. Umoret, A. Vagnerini, S. Belforte, V. Candelise, M. Casarsa, F. Cossutti, A. Da Rold, G. Della Ricca, G. Sorrentino, S. Dogra, C. Huh, B. Kim, D. H. Kim, G. N. Kim, J. Kim, J. Lee, S. W. Lee, C. S. Moon, Y. D. Oh, S. I. Pak, S. Sekmen, Y. C. Yang, H. Kim, D. H. Moon, E. Asilar, T. J. Kim, J. Park, S. Cho, S. Choi, S. Han, B. Hong, K. Lee, K. S. Lee, J. Lim, J. Park, S. K. Park, J. Yoo, J. Goh, H. S. Kim, Y. Kim, S. Lee, J. Almond, J. H. Bhyun, J. Choi, S. Jeon, W. Jun, J. S. Kim, J. Kim, S. Ko, H. Kwon, H. Lee, J. Lee, S. Lee, B. H. Oh, M. Oh, S. B. Oh, H. Seo, U. K. Yang, I. Yoon, W. Jang, D. Y. Kang, Y. Kang, D. Kim, S. Kim, B. Ko, J. S. H. Lee, Y. Lee, J. A. Merlin, I. C. Park, Y. Roh, M. S. Ryu, D. Song, I. J. Watson, S. Yang, S. Ha, H. D. Yoo, M. Choi, H. Lee, Y. Lee, I. Yu, T. Beyrouthy, Y. Maghrbi, K. Dreimanis, A. Gaile, A. Potrebko, T. Torims, V. Veckalns, M. Ambrozas, A. Carvalho Antunes De Oliveira, A. Juodagalvis, A. Rinkevicius, G. Tamulaitis, N. Bin Norjoharuddeen, S. Y. Hoh, I. Yusuff, Z. Zolkapli, J. F. Benitez, A. Castaneda Hernandez, H. A. Encinas Acosta, L. G. Gallegos Maríñez, M. León Coello, J. A. Murillo Quijada, A. Sehrawat, L. Valencia Palomo, G. Ayala, H. Castilla-Valdez, E. De La Cruz-Burelo, I. Heredia-De La Cruz, R. Lopez-Fernandez, C. A. Mondragon Herrera, D. A. Perez Navarro, A. Sánchez Hernández, C. Oropeza Barrera, F. Vazquez Valencia, I. Pedraza, H. A. Salazar Ibarguen, C. Uribe Estrada, I. Bubanja, J. Mijuskovic, N. Raicevic, A. Ahmad, M. I. Asghar, A. Awais, M. I. M. Awan, M. Gul, H. R. Hoorani, W. A. Khan, V. Avati, L. Grzanka, M. Malawski, H. Bialkowska, M. Bluj, B. Boimska, M. Górski, M. Kazana, M. Szleper, P. Zalewski, K. Bunkowski, K. Doroba, A. Kalinowski, M. Konecki, J. Krolikowski, M. Araujo, P. Bargassa, D. Bastos, A. Boletti, P. Faccioli, M. Gallinaro, J. Hollar, N. Leonardo, T. Niknejad, M. Pisano, J. Seixas, O. Toldaiev, J. Varela, P. Adzic, M. Dordevic, P. Milenovic, J. Milosevic, M. Aguilar-Benitez, J. Alcaraz Maestre, A. Álvarez Fernández, M. Barrio Luna, C. A. Carrillo Montoya, M. Cepeda, M. Cerrada, N. Colino, B. De La Cruz, A. Delgado Peris, Cristina F. Bedoya, D. Fernández Del Val, J. P. Fernández Ramos, J. Flix, M. C. Fouz, O. Gonzalez Lopez, S. Goy Lopez, J. M. Hernandez, M. I. Josa, J. León Holgado, D. Moran, C. Perez Dengra, A. Pérez-Calero Yzquierdo, J. Puerta Pelayo, D. D. Redondo Ferrero, I. Redondo, L. Romero, S. Sánchez Navas, J. Sastre, L. Urda Gómez, J. Vazquez Escobar, C. Willmott, J. F. de Trocóniz, B. Alvarez Gonzalez, J. Cuevas, J. Fernandez Menendez, S. Folgueras, I. Gonzalez Caballero, J. R. González Fernández, E. Palencia Cortezon, C. Ramón Álvarez, V. Rodríguez Bouza, A. Soto Rodríguez, A. Trapote, C. Vico Villalba, J. A. Brochero Cifuentes, I. J. Cabrillo, A. Calderon, J. Duarte Campderros, C. Fernandez Madrazo, M. Fernandez, A. García Alonso, G. Gomez, C. Lasaosa García, C. Martinez Rivero, P. Martinez Ruiz del Arbol, P. Matorras Cuevas, F. Matorras, J. Piedra Gomez, C. Prieels, A. Ruiz-Jimeno, L. Scodellaro, I. Vila, J. M. Vizan Garcia, D. D. C. Wickramarathna, B. Kailasapathy, M. K. Jayananda, D. U. J. Sonnadara, W. G. D. Dharmaratna, K. Liyanage, N. Perera, N. Wickramage, D. Abbaneo, J. Alimena, E. Auffray, G. Auzinger, J. Baechler, P. Baillon, D. Barney, J. Bendavid, M. Bianco, B. Bilin, A. Bocci, E. Brondolin, C. Caillol, T. Camporesi, G. Cerminara, N. Chernyavskaya, S. S. Chhibra, S. Choudhury, M. Cipriani, L. Cristella, D. d’Enterria, A. Dabrowski, A. David, A. De Roeck, M. M. Defranchis, M. Deile, M. Dobson, M. Dünser, N. Dupont, A. Elliott-Peisert, F. Fallavollita, A. Florent, L. Forthomme, G. Franzoni, W. Funk, S. Ghosh, S. Giani, D. Gigi, K. Gill, F. Glege, L. Gouskos, E. Govorkova, M. Haranko, J. Hegeman, V. Innocente, T. James, P. Janot, J. Kaspar, J. Kieseler, N. Kratochwil, S. Laurila, P. Lecoq, A. Lintuluoto, C. Lourenço, B. Maier, L. Malgeri, M. Mannelli, A. C. Marini, F. Meijers, S. Mersi, E. Meschi, F. Moortgat, M. Mulders, S. Orfanelli, L. Orsini, F. Pantaleo, E. Perez, M. Peruzzi, A. Petrilli, G. Petrucciani, A. Pfeiffer, M. Pierini, D. Piparo, M. Pitt, H. Qu, T. Quast, D. Rabady, A. Racz, G. Reales Gutiérrez, M. Rovere, H. Sakulin, J. Salfeld-Nebgen, S. Scarfi, M. Selvaggi, A. Sharma, P. Silva, P. Sphicas, A. G. Stahl Leiton, S. Summers, K. Tatar, V. R. Tavolaro, D. Treille, P. Tropea, A. Tsirou, J. Wanczyk, K. A. Wozniak, W. D. Zeuner, L. Caminada, A. Ebrahimi, W. Erdmann, R. Horisberger, Q. Ingram, H. C. Kaestli, D. Kotlinski, C. Lange, M. Missiroli, L. Noehte, T. Rohe, T. K. Aarrestad, K. Androsov, M. Backhaus, P. Berger, A. Calandri, K. Datta, A. De Cosa, G. Dissertori, M. Dittmar, M. Donegà, F. Eble, M. Galli, K. Gedia, F. Glessgen, T. A. Gómez Espinosa, C. Grab, D. Hits, W. Lustermann, A.-M. Lyon, R. A. Manzoni, L. Marchese, C. Martin Perez, A. Mascellani, M. T. Meinhard, F. Nessi-Tedaldi, J. Niedziela, F. Pauss, V. Perovic, S. Pigazzini, M. G. Ratti, M. Reichmann, C. Reissel, T. Reitenspiess, B. Ristic, F. Riti, D. Ruini, D. A. Sanz Becerra, J. Steggemann, D. Valsecchi, R. Wallny, C. Amsler, P. Bärtschi, C. Botta, D. Brzhechko, M. F. Canelli, K. Cormier, A. De Wit, R. Del Burgo, J. K. Heikkilä, M. Huwiler, W. Jin, A. Jofrehei, B. Kilminster, S. Leontsinis, S. P. Liechti, A. Macchiolo, P. Meiring, V. M. Mikuni, U. Molinatti, I. Neutelings, A. Reimers, P. Robmann, S. Sanchez Cruz, K. Schweiger, M. Senger, Y. Takahashi, C. Adloff, C. M. Kuo, W. Lin, S. S. Yu, L. Ceard, Y. Chao, K. F. Chen, P. S. Chen, H. Cheng, W.-S. Hou, Y. Y. Li, R.-S. Lu, E. Paganis, A. Psallidas, A. Steen, H. Y. Wu, E. Yazgan, P. R. Yu, C. Asawatangtrakuldee, N. Srimanobhas, D. Agyel, F. Boran, Z. S. Demiroglu, F. Dolek, I. Dumanoglu, E. Eskut, Y. Guler, E. Gurpinar Guler, C. Isik, O. Kara, A. Kayis Topaksu, U. Kiminsu, G. Onengut, K. Ozdemir, A. Polatoz, A. E. Simsek, B. Tali, U. G. Tok, S. Turkcapar, E. Uslan, I. S. Zorbakir, G. Karapinar, K. Ocalan, M. Yalvac, B. Akgun, I. O. Atakisi, E. Gülmez, M. Kaya, O. Kaya, Ö. Özçelik, S. Tekten, A. Cakir, K. Cankocak, Y. Komurcu, S. Sen, O. Aydilek, S. Cerci, B. Hacisahinoglu, I. Hos, B. Isildak, B. Kaynak, S. Ozkorucuklu, C. Simsek, D. Sunar Cerci, B. Grynyov, L. Levchuk, D. Anthony, E. Bhal, J. J. Brooke, A. Bundock, E. Clement, D. Cussans, H. Flacher, M. Glowacki, J. Goldstein, G. P. Heath, H. F. Heath, L. Kreczko, B. Krikler, S. Paramesvaran, S. Seif El Nasr-Storey, V. J. Smith, N. Stylianou, K. Walkingshaw Pass, R. White, A. H. Ball, K. W. Bell, A. Belyaev, C. Brew, R. M. Brown, D. J. A. Cockerill, C. Cooke, K. V. Ellis, K. Harder, S. Harper, M.-L. Holmberg, J. Linacre, K. Manolopoulos, D. M. Newbold, E. Olaiya, D. Petyt, T. Reis, G. Salvi, T. Schuh, C. H. Shepherd-Themistocleous, I. R. Tomalin, T. Williams, R. Bainbridge, P. Bloch, S. Bonomally, J. Borg, S. Breeze, C. E. Brown, O. Buchmuller, V. Cacchio, V. Cepaitis, G. S. Chahal, D. Colling, J. S. Dancu, P. Dauncey, G. Davies, J. Davies, M. Della Negra, S. Fayer, G. Fedi, G. Hall, M. H. Hassanshahi, A. Howard, G. Iles, J. Langford, L. Lyons, A.-M. Magnan, S. Malik, A. Martelli, M. Mieskolainen, D. G. Monk, J. Nash, M. Pesaresi, B. C. Radburn-Smith, D. M. Raymond, A. Richards, A. Rose, E. Scott, C. Seez, A. Shtipliyski, R. Shukla, A. Tapper, K. Uchida, G. P. Uttley, L. H. Vage, T. Virdee, M. Vojinovic, N. Wardle, S. N. Webb, D. Winterbottom, K. Coldham, J. E. Cole, A. Khan, P. Kyberd, I. D. Reid, L. Teodorescu, S. Abdullin, A. Brinkerhoff, B. Caraway, J. Dittmann, K. Hatakeyama, A. R. Kanuganti, B. McMaster, M. Saunders, S. Sawant, C. Sutantawibul, J. Wilson, R. Bartek, A. Dominguez, R. Uniyal, A. M. Vargas Hernandez, A. Buccilli, S. I. Cooper, D. Di Croce, S. V. Gleyzer, C. Henderson, C. U. Perez, P. Rumerio, C. West, A. Akpinar, A. Albert, D. Arcaro, C. Cosby, Z. Demiragli, C. Erice, E. Fontanesi, D. Gastler, S. May, J. Rohlf, K. Salyer, D. Sperka, D. Spitzbart, I. Suarez, A. Tsatsos, S. Yuan, G. Benelli, B. Burkle, X. Coubez, D. Cutts, M. Hadley, U. Heintz, J. M. Hogan, T. Kwon, G. Landsberg, K. T. Lau, D. Li, J. Luo, M. Narain, N. Pervan, S. Sagir, F. Simpson, E. Usai, W. Y. Wong, X. Yan, D. Yu, W. Zhang, J. Bonilla, C. Brainerd, R. Breedon, M. Calderon De La Barca Sanchez, M. Chertok, J. Conway, P. T. Cox, R. Erbacher, G. Haza, F. Jensen, O. Kukral, G. Mocellin, M. Mulhearn, D. Pellett, B. Regnery, D. Taylor, Y. Yao, F. Zhang, M. Bachtis, R. Cousins, A. Datta, D. Hamilton, J. Hauser, M. Ignatenko, M. A. Iqbal, T. Lam, W. A. Nash, S. Regnard, D. Saltzberg, B. Stone, V. Valuev, Y. Chen, R. Clare, J. W. Gary, M. Gordon, G. Hanson, G. Karapostoli, O. R. Long, N. Manganelli, W. Si, S. Wimpenny, J. G. Branson, P. Chang, S. Cittolin, S. Cooperstein, D. Diaz, J. Duarte, R. Gerosa, L. Giannini, J. Guiang, R. Kansal, V. Krutelyov, R. Lee, J. Letts, M. Masciovecchio, F. Mokhtar, M. Pieri, B. V. Sathia Narayanan, V. Sharma, M. Tadel, F. Würthwein, Y. Xiang, A. Yagil, N. Amin, C. Campagnari, M. Citron, G. Collura, A. Dorsett, V. Dutta, J. Incandela, M. Kilpatrick, J. Kim, A. J. Li, B. Marsh, P. Masterson, H. Mei, M. Oshiro, M. Quinnan, J. Richman, U. Sarica, R. Schmitz, F. Setti, J. Sheplock, P. Siddireddy, D. Stuart, S. Wang, A. Bornheim, O. Cerri, I. Dutta, J. M. Lawhorn, N. Lu, J. Mao, H. B. Newman, T. Q. Nguyen, M. Spiropulu, J. R. Vlimant, C. Wang, S. Xie, Z. Zhang, R. Y. Zhu, J. Alison, S. An, M. B. Andrews, P. Bryant, T. Ferguson, A. Harilal, C. Liu, T. Mudholkar, S. Murthy, M. Paulini, A. Roberts, A. Sanchez, W. Terrill, J. P. Cumalat, W. T. Ford, A. Hassani, G. Karathanasis, E. MacDonald, F. Marini, R. Patel, A. Perloff, C. Savard, N. Schonbeck, K. Stenson, K. A. Ulmer, S. R. Wagner, N. Zipper, J. Alexander, S. Bright-Thonney, X. Chen, D. J. Cranshaw, J. Fan, X. Fan, D. Gadkari, S. Hogan, J. Monroy, J. R. Patterson, D. Quach, J. Reichert, M. Reid, A. Ryd, J. Thom, P. Wittich, R. Zou, M. Albrow, M. Alyari, G. Apollinari, A. Apresyan, L. A. T. Bauerdick, D. Berry, J. Berryhill, P. C. Bhat, K. Burkett, J. N. Butler, A. Canepa, G. B. Cerati, H. W. K. Cheung, F. Chlebana, K. F. Di Petrillo, J. Dickinson, V. D. Elvira, Y. Feng, J. Freeman, A. Gandrakota, Z. Gecse, L. Gray, D. Green, S. Grünendahl, O. Gutsche, R. M. Harris, R. Heller, T. C. Herwig, J. Hirschauer, L. Horyn, B. Jayatilaka, S. Jindariani, M. Johnson, U. Joshi, T. Klijnsma, B. Klima, K. H. M. Kwok, S. Lammel, D. Lincoln, R. Lipton, T. Liu, C. Madrid, K. Maeshima, C. Mantilla, D. Mason, P. McBride, P. Merkel, S. Mrenna, S. Nahn, J. Ngadiuba, V. Papadimitriou, N. Pastika, K. Pedro, C. Pena, F. Ravera, A. Reinsvold Hall, L. Ristori, E. Sexton-Kennedy, N. Smith, A. Soha, L. Spiegel, J. Strait, L. Taylor, S. Tkaczyk, N. V. Tran, L. Uplegger, E. W. Vaandering, H. A. Weber, I. Zoi, P. Avery, D. Bourilkov, L. Cadamuro, V. Cherepanov, R. D. Field, D. Guerrero, M. Kim, E. Koenig, J. Konigsberg, A. Korytov, K. H. Lo, K. Matchev, N. Menendez, G. Mitselmakher, A. Muthirakalayil Madhu, N. Rawal, D. Rosenzweig, S. Rosenzweig, K. Shi, J. Wang, Z. Wu, T. Adams, A. Askew, R. Habibullah, V. Hagopian, R. Khurana, T. Kolberg, G. Martinez, H. Prosper, C. Schiber, O. Viazlo, R. Yohay, J. Zhang, M. M. Baarmand, S. Butalla, T. Elkafrawy, M. Hohlmann, R. Kumar Verma, D. Noonan, M. Rahmani, F. Yumiceva, M. R. Adams, H. Becerril Gonzalez, R. Cavanaugh, D. S. Lemos, S. Dittmer, O. Evdokimov, C. E. Gerber, D. J. Hofman, A. H. Merrit, C. Mills, G. Oh, T. Roy, S. Rudrabhatla, M. B. Tonjes, N. Varelas, X. Wang, Z. Ye, J. Yoo, M. Alhusseini, K. Dilsiz, L. Emediato, R. P. Gandrajula, G. Karaman, O. K. Köseyan, J.-P. Merlo, A. Mestvirishvili, J. Nachtman, O. Neogi, H. Ogul, Y. Onel, A. Penzo, C. Snyder, E. Tiras, O. Amram, B. Blumenfeld, L. Corcodilos, J. Davis, A. V. Gritsan, L. Kang, S. Kyriacou, P. Maksimovic, J. Roskes, S. Sekhar, M. Swartz, T. Á. Vámi, A. Abreu, L. F. Alcerro Alcerro, J. Anguiano, P. Baringer, A. Bean, Z. Flowers, T. Isidori, S. Khalil, J. King, G. Krintiras, M. Lazarovits, C. Le Mahieu, C. Lindsey, J. Marquez, N. Minafra, M. Murray, M. Nickel, C. Rogan, C. Royon, R. Salvatico, S. Sanders, E. Schmitz, C. Smith, Q. Wang, Z. Warner, J. Williams, G. Wilson, B. Allmond, S. Duric, R. Gujju Gurunadha, A. Ivanov, K. Kaadze, D. Kim, Y. Maravin, T. Mitchell, A. Modak, K. Nam, J. Natoli, D. Roy, F. Rebassoo, D. Wright, E. Adams, A. Baden, O. Baron, A. Belloni, A. Bethani, S. C. Eno, N. J. Hadley, S. Jabeen, R. G. Kellogg, T. Koeth, Y. Lai, S. Lascio, A. C. Mignerey, S. Nabili, C. Palmer, C. Papageorgakis, M. Seidel, L. Wang, K. Wong, D. Abercrombie, R. Bi, W. Busza, I. A. Cali, Y. Chen, M. D’Alfonso, J. Eysermans, C. Freer, G. Gomez-Ceballos, M. Goncharov, P. Harris, M. Hu, D. Kovalskyi, J. Krupa, Y.-J. Lee, K. Long, C. Mironov, C. Paus, D. Rankin, C. Roland, G. Roland, Z. Shi, G. S. F. Stephans, J. Wang, Z. Wang, B. Wyslouch, R. M. Chatterjee, B. Crossman, A. Evans, J. Hiltbrand, Sh. Jain, B. M. Joshi, C. Kapsiak, M. Krohn, Y. Kubota, J. Mans, M. Revering, R. Rusack, R. Saradhy, N. Schroeder, N. Strobbe, M. A. Wadud, L. M. Cremaldi, K. Bloom, M. Bryson, S. Chauhan, D. R. Claes, C. Fangmeier, L. Finco, F. Golf, C. Joo, I. Kravchenko, I. Reed, J. E. Siado, G. R. Snow, W. Tabb, A. Wightman, F. Yan, A. G. Zecchinelli, G. Agarwal, H. Bandyopadhyay, L. Hay, I. Iashvili, A. Kharchilava, C. McLean, M. Morris, D. Nguyen, J. Pekkanen, S. Rappoccio, A. Williams, G. Alverson, E. Barberis, Y. Haddad, Y. Han, A. Krishna, J. Li, J. Lidrych, G. Madigan, B. Marzocchi, D. M. Morse, V. Nguyen, T. Orimoto, A. Parker, L. Skinnari, A. Tishelman-Charny, T. Wamorkar, B. Wang, A. Wisecarver, D. Wood, S. Bhattacharya, J. Bueghly, Z. Chen, A. Gilbert, T. Gunter, K. A. Hahn, Y. Liu, N. Odell, M. H. Schmitt, M. Velasco, R. Band, R. Bucci, S. Castells, M. Cremonesi, A. Das, R. Goldouzian, M. Hildreth, K. Hurtado Anampa, C. Jessop, K. Lannon, J. Lawrence, N. Loukas, L. Lutton, J. Mariano, N. Marinelli, I. Mcalister, T. McCauley, C. Mcgrady, K. Mohrman, C. Moore, Y. Musienko, H. Nelson, R. Ruchti, A. Townsend, M. Wayne, H. Yockey, M. Zarucki, L. Zygala, B. Bylsma, M. Carrigan, L. S. Durkin, B. Francis, C. Hill, A. Lesauvage, M. Nunez Ornelas, K. Wei, B. L. Winer, B. R. Yates, F. M. Addesa, B. Bonham, P. Das, G. Dezoort, P. Elmer, A. Frankenthal, B. Greenberg, N. Haubrich, S. Higginbotham, A. Kalogeropoulos, G. Kopp, S. Kwan, D. Lange, D. Marlow, K. Mei, I. Ojalvo, J. Olsen, D. Stickland, C. Tully, S. Malik, S. Norberg, A. S. Bakshi, V. E. Barnes, R. Chawla, S. Das, L. Gutay, M. Jones, A. W. Jung, D. Kondratyev, A. M. Koshy, M. Liu, G. Negro, N. Neumeister, G. Paspalaki, S. Piperov, A. Purohit, J. F. Schulte, M. Stojanovic, J. Thieman, F. Wang, R. Xiao, W. Xie, J. Dolen, N. Parashar, D. Acosta, A. Baty, T. Carnahan, M. Decaro, S. Dildick, K. M. Ecklund, P. J. Fernández Manteca, S. Freed, P. Gardner, F. J. M. Geurts, A. Kumar, W. Li, B. P. Padley, R. Redjimi, J. Rotter, W. Shi, S. Yang, E. Yigitbasi, L. Zhang, Y. Zhang, X. Zuo, A. Bodek, P. de Barbaro, R. Demina, J. L. Dulemba, C. Fallon, T. Ferbel, M. Galanti, A. Garcia-Bellido, O. Hindrichs, A. Khukhunaishvili, E. Ranken, R. Taus, G. P. Van Onsem, K. Goulianos, B. Chiarito, J. P. Chou, Y. Gershtein, E. Halkiadakis, A. Hart, M. Heindl, O. Karacheban, I. Laflotte, A. Lath, R. Montalvo, K. Nash, M. Osherson, S. Salur, S. Schnetzer, S. Somalwar, R. Stone, S. A. Thayil, S. Thomas, H. Wang, H. Acharya, A. G. Delannoy, S. Fiorendi, T. Holmes, E. Nibigira, S. Spanier, O. Bouhali, M. Dalchenko, A. Delgado, R. Eusebi, J. Gilmore, T. Huang, T. Kamon, H. Kim, S. Luo, S. Malhotra, R. Mueller, D. Overton, D. Rathjens, A. Safonov, N. Akchurin, J. Damgov, V. Hegde, K. Lamichhane, S. W. Lee, T. Mengke, S. Muthumuni, T. Peltola, I. Volobouev, Z. Wang, A. Whitbeck, E. Appelt, S. Greene, A. Gurrola, W. Johns, A. Melo, F. Romeo, P. Sheldon, S. Tuo, J. Velkovska, J. Viinikainen, B. Cardwell, B. Cox, G. Cummings, J. Hakala, R. Hirosky, M. Joyce, A. Ledovskoy, A. Li, C. Neu, C. E. Perez Lara, B. Tannenwald, P. E. Karchin, N. Poudyal, S. Banerjee, K. Black, T. Bose, S. Dasu, I. De Bruyn, P. Everaerts, C. Galloni, H. He, M. Herndon, A. Herve, C. K. Koraka, A. Lanaro, A. Loeliger, R. Loveless, J. Madhusudanan Sreekala, A. Mallampalli, A. Mohammadi, S. Mondal, G. Parida, D. Pinna, A. Savin, V. Shang, V. Sharma, W. H. Smith, D. Teague, H. F. Tsoi, W. Vetens, S. Afanasiev, V. Andreev, Yu. Andreev, T. Aushev, M. Azarkin, A. Babaev, A. Belyaev, V. Blinov, E. Boos, V. Borshch, D. Budkouski, V. Chekhovsky, R. Chistov, A. Dermenev, T. Dimova, I. Dremin, M. Dubinin, L. Dudko, V. Epshteyn, A. Ershov, G. Gavrilov, V. Gavrilov, S. Gninenko, V. Golovtcov, N. Golubev, I. Golutvin, I. Gorbunov, A. Gribushin, Y. Ivanov, V. Ivanchenko, V. Kachanov, L. Kardapoltsev, V. Karjavine, A. Karneyeu, V. Kim, M. Kirakosyan, D. Kirpichnikov, M. Kirsanov, V. Klyukhin, O. Kodolova, D. Konstantinov, V. Korenkov, A. Kozyrev, N. Krasnikov, E. Kuznetsova, A. Lanev, P. Levchenko, A. Litomin, N. Lychkovskaya, V. Makarenko, A. Malakhov, V. Matveev, V. Murzin, A. Nikitenko, S. Obraztsov, V. Okhotnikov, A. Oskin, I. Ovtin, V. Palichik, P. Parygin, V. Perelygin, S. Petrushanko, G. Pivovarov, S. Polikarpov, V. Popov, E. Popova, O. Radchenko, M. Savina, V. Savrin, D. Selivanova, V. Shalaev, S. Shmatov, S. Shulha, Y. Skovpen, S. Slabospitskii, V. Smirnov, A. Snigirev, D. Sosnov, A. Stepennov, V. Sulimov, E. Tcherniaev, A. Terkulov, O. Teryaev, I. Tlisova, M. Toms, A. Toropin, L. Uvarov, A. Uzunian, E. Vlasov, A. Vorobyev, N. Voytishin, B. S. Yuldashev, A. Zarubin, I. Zhizhin, A. Zhokin

**Affiliations:** 1 Yerevan Physics Institute, Yerevan, Armenia; 2 Institut für Hochenergiephysik, Vienna, Austria; 3 Universiteit Antwerpen, Antwerpen, Belgium; 4 Vrije Universiteit Brussel, Brussel, Belgium; 5 Université Libre de Bruxelles, Bruxelles, Belgium; 6 Ghent University, Ghent, Belgium; 7 Université Catholique de Louvain, Louvain-la-Neuve, Belgium; 8 Centro Brasileiro de Pesquisas Fisicas, Rio de Janeiro, Brazil; 9 Universidade do Estado do Rio de Janeiro, Rio de Janeiro, Brazil; 10 Universidade Estadual Paulista, Universidade Federal do ABC, São Paulo, Brazil; 11 Institute for Nuclear Research and Nuclear Energy, Bulgarian Academy of Sciences, Sofia, Bulgaria; 12 University of Sofia, Sofia, Bulgaria; 13 Beihang University, Beijing, China; 14 Department of Physics, Tsinghua University, Beijing, China; 15 Institute of High Energy Physics, Beijing, China; 16 State Key Laboratory of Nuclear Physics and Technology, Peking University, Beijing, China; 17 Sun Yat-Sen University, Guangzhou, China; 18 Institute of Modern Physics and Key Laboratory of Nuclear Physics and Ion-beam Application (MOE) -Fudan University, Shanghai, China; 19 Zhejiang University, Hangzhou, Zhejiang, China; 20 Universidad de Los Andes, Bogota, Colombia; 21 Universidad de Antioquia, Medellin, Colombia; 22 University of Split, Faculty of Electrical Engineering, Mechanical Engineering and Naval Architecture, Split, Croatia; 23 University of Split, Faculty of Science, Split, Croatia; 24 Institute Rudjer Boskovic, Zagreb, Croatia; 25 University of Cyprus, Nicosia, Cyprus; 26 Charles University, Prague, Czech Republic; 27 Escuela Politecnica Nacional, Quito, Ecuador; 28 Universidad San Francisco de Quito, Quito, Ecuador; 29 Academy of Scientific Research and Technology of the Arab Republic of Egypt, Egyptian Network of High EnergyPhysics, Cairo, Egypt; 30 Center for High Energy Physics (CHEP-FU), Fayoum University, El-Fayoum, Egypt; 31 National Institute of Chemical Physics and Biophysics, Tallinn, Estonia; 32 Department of Physics, University of Helsinki, Helsinki, Finland; 33 Helsinki Institute of Physics, Helsinki, Finland; 34 Lappeenranta-Lahti University of Technology, Lappeenranta, Finland; 35 IRFU, CEA, Université Paris-Saclay, Gif-sur-Yvette, France; 36 Laboratoire Leprince-Ringuet, CNRS/IN2P3, Ecole Polytechnique, Institut Polytechnique de Paris, Palaiseau, France; 37 Université de Strasbourg, CNRS, IPHC UMR 7178, Strasbourg, France; 38 Institut de Physique des 2 Infinis de Lyon (IP2I), Villeurbanne, France; 39 Georgian Technical University, Tbilisi, Georgia; 40 RWTH Aachen University, I. Physikalisches Institut, Aachen, Germany; 41 RWTH Aachen University, III. Physikalisches Institut A, Aachen, Germany; 42 RWTH Aachen University, III. Physikalisches Institut B, Aachen, Germany; 43 Deutsches Elektronen-Synchrotron, Hamburg, Germany; 44 University of Hamburg, Hamburg, Germany; 45 Karlsruher Institut fuer Technologie, Karlsruhe, Germany; 46 Institute of Nuclear and Particle Physics (INPP), NCSR Demokritos, Aghia Paraskevi, Greece; 47 National and Kapodistrian University of Athens, Athens, Greece; 48 National Technical University of Athens, Athens, Greece; 49 University of Ioánnina, Ioánnina, Greece; 50 MTA-ELTE Lendület CMS Particle and Nuclear Physics Group, Eötvös Loránd University, Budapest, Hungary; 51 Wigner Research Centre for Physics, Budapest, Hungary; 52 Institute of Nuclear Research ATOMKI, Debrecen, Hungary; 53 Institute of Physics, University of Debrecen, Debrecen, Hungary; 54 Karoly Robert Campus, MATE Institute of Technology, Gyongyos, Hungary; 55 Panjab University, Chandigarh, India; 56 University of Delhi, Delhi, India; 57 Saha Institute of Nuclear Physics, HBNI, Kolkata, India; 58 Indian Institute of Technology Madras, Madras, India; 59 Bhabha Atomic Research Centre, Mumbai, India; 60 Tata Institute of Fundamental Research-A, Mumbai, India; 61 Tata Institute of Fundamental Research-B, Mumbai, India; 62 National Institute of Science Education and Research, An OCC of Homi Bhabha National Institute, Bhubaneswar, Odisha India; 63 Indian Institute of Science Education and Research (IISER), Pune, India; 64 Isfahan University of Technology, Isfahan, Iran; 65 Institute for Research in Fundamental Sciences (IPM), Tehran, Iran; 66 University College Dublin, Dublin, Ireland; 67 INFN Sezione di Bari, Bari, Italy; 68 Università di Bari, Bari, Italy; 69 Politecnico di Bari, Bari, Italy; 70 INFN Sezione di Bologna, Bologna, Italy; 71 Università di Bologna, Bologna, Italy; 72 INFN Sezione di Catania, Catania, Italy; 73 Università di Catania, Catania, Italy; 74 INFN Sezione di Firenze, Firenze, Italy; 75 Università di Firenze, Firenze, Italy; 76 INFN Laboratori Nazionali di Frascati, Frascati, Italy; 77 INFN Sezione di Genova, Genova, Italy; 78 Università di Genova, Genova, Italy; 79 INFN Sezione di Milano-Bicocca, Milano, Italy; 80 Università di Milano-Bicocca, Milano, Italy; 81 INFN Sezione di Napoli, Napoli, Italy; 82 Università di Napoli ’Federico II’, Napoli, Italy; 83 Università della Basilicata, Potenza, Italy; 84 Università G. Marconi, Roma, Italy; 85 INFN Sezione di Padova, Padova, Italy; 86 Università di Padova, Padova, Italy; 87 Università di Trento, Trento, Italy; 88 INFN Sezione di Pavia, Pavia, Italy; 89 Università di Pavia, Pavia, Italy; 90 INFN Sezione di Perugia, Perugia, Italy; 91 Università di Perugia, Perugia, Italy; 92 INFN Sezione di Pisa, Pisa, Italy; 93 Scuola Normale Superiore di Pisa, Pisa, Italy; 94 Università di Pisa, Pisa, Italy; 95 Università di Siena, Siena, Italy; 96 INFN Sezione di Roma, Roma, Italy; 97 Sapienza Università di Roma, Roma, Italy; 98 INFN Sezione di Torino, Torino, Italy; 99 Università di Torino, Torino, Italy; 100 Università del Piemonte Orientale, Novara, Italy; 101 INFN Sezione di Trieste, Trieste, Italy; 102 Università di Trieste, Trieste, Italy; 103 Kyungpook National University, Daegu, Korea; 104 Chonnam National University, Institute for Universe and Elementary Particles, Kwangju, Korea; 105 Hanyang University, Seoul, Korea; 106 Korea University, Seoul, Korea; 107 Kyung Hee University, Department of Physics, Seoul, Korea; 108 Sejong University, Seoul, Korea; 109 Seoul National University, Seoul, Korea; 110 University of Seoul, Seoul, Korea; 111 Yonsei University, Department of Physics, Seoul, Korea; 112 Sungkyunkwan University, Suwon, Korea; 113 College of Engineering and Technology, American University of the Middle East (AUM), Dasman, Kuwait; 114 Riga Technical University, Riga, Latvia; 115 Vilnius University, Vilnius, Lithuania; 116 National Centre for Particle Physics, Universiti Malaya, Kuala Lumpur, Malaysia; 117 Universidad de Sonora (UNISON), Hermosillo, Mexico; 118 Centro de Investigacion y de Estudios Avanzados del IPN, Mexico City, Mexico; 119 Universidad Iberoamericana, Mexico City, Mexico; 120 Benemerita Universidad Autonoma de Puebla, Puebla, Mexico; 121 University of Montenegro, Podgorica, Montenegro; 122 National Centre for Physics, Quaid-I-Azam University, Islamabad, Pakistan; 123 AGH University of Science and Technology Faculty of Computer Science, Electronics and Telecommunications, Krakow, Poland; 124 National Centre for Nuclear Research, Swierk, Poland; 125 Institute of Experimental Physics, Faculty of Physics, University of Warsaw, Warsaw, Poland; 126 Laboratório de Instrumentação e Física Experimental de Partículas, Lisboa, Portugal; 127 VINCA Institute of Nuclear Sciences, University of Belgrade, Belgrade, Serbia; 128 Centro de Investigaciones Energéticas Medioambientales y Tecnológicas (CIEMAT), Madrid, Spain; 129 Universidad Autónoma de Madrid, Madrid, Spain; 130 Universidad de Oviedo, Instituto Universitario de Ciencias y Tecnologías Espaciales de Asturias (ICTEA), Oviedo, Spain; 131 Instituto de Física de Cantabria (IFCA), CSIC-Universidad de Cantabria, Santander, Spain; 132 University of Colombo, Colombo, Sri Lanka; 133 University of Ruhuna, Department of Physics, Matara, Sri Lanka; 134 CERN, European Organization for Nuclear Research, Geneva, Switzerland; 135 Paul Scherrer Institut, Villigen, Switzerland; 136 ETH Zurich -Institute for Particle Physics and Astrophysics (IPA), Zurich, Switzerland; 137 Universität Zürich, Zurich, Switzerland; 138 National Central University, Chung-Li, Taiwan; 139 National Taiwan University (NTU), Taipei, Taiwan; 140 Chulalongkorn University, Faculty of Science, Department of Physics, Bangkok, Thailand; 141 ukurovaÇ University, Physics Department, Science and Art Faculty, Adana, Turkey; 142 Middle East Technical University, Physics Department, Ankara, Turkey; 143 Bogazici University, Istanbul, Turkey; 144 Istanbul Technical University, Istanbul, Turkey; 145 Istanbul University, Istanbul, Turkey; 146 Institute for Scintillation Materials of National Academy of Science of Ukraine, Kharkiv, Ukraine; 147 National Science Centre, Kharkiv Institute of Physics and Technology, Kharkiv, Ukraine; 148 University of Bristol, Bristol, United Kingdom; 149 Rutherford Appleton Laboratory, Didcot, United Kingdom; 150 Imperial College, London, United Kingdom; 151 Brunel University, Uxbridge, United Kingdom; 152 Baylor University, Waco, Texas USA; 153 Catholic University of America, Washington, DC USA; 154 The University of Alabama, Tuscaloosa, Alabama USA; 155 Boston University, Boston, Massachusetts, USA; 156 Brown University, Providence, Rhode Island USA; 157 University of California, Davis, Davis, California USA; 158 University of California, Los Angeles, California USA; 159 University of California, Riverside, Riverside, California USA; 160 University of California, San Diego, La Jolla, California, USA; 161 University of California, Santa Barbara -Department of Physics, Santa Barbara, California, USA; 162 California Institute of Technology, Pasadena, California, USA; 163 Carnegie Mellon University, Pittsburgh, Pennsylvania USA; 164 University of Colorado Boulder, Boulder, Colorado, USA; 165 Cornell University, Ithaca, New York, USA; 166 Fermi National Accelerator Laboratory, Batavia, Illinois USA; 167 California Institute of Technology, Pasadena, California USA; 168 University of Florida, Gainesville, Florida USA; 169 Florida State University, Tallahassee, Florida USA; 170 Florida Institute of Technology, Melbourne, Florida USA; 171 University of Illinois at Chicago (UIC), Chicago, Illinois USA; 172 The University of Iowa, Iowa City, Iowa USA; 173 Johns Hopkins University, Baltimore, Maryland USA; 174 The University of Kansas, Lawrence, Kansas USA; 175 Kansas State University, Manhattan, Kansas USA; 176 Lawrence Livermore National Laboratory, Livermore, California USA; 177 University of Maryland, College Park, Maryland USA; 178 Massachusetts Institute of Technology, Cambridge, Massachusetts USA; 179 University of Minnesota, Minneapolis, Minnesota USA; 180 University of Mississippi, Oxford, Mississippi USA; 181 University of Nebraska-Lincoln, Lincoln, Nebraska USA; 182 State University of New York at Buffalo, Buffalo, New York USA; 183 Northeastern University, Boston, Massachusetts USA; 184 Northwestern University, Evanston, Illinois USA; 185 University of Notre Dame, Notre Dame, Indiana, USA; 186 The Ohio State University, Columbus, Ohio USA; 187 Princeton University, Princeton, New Jersey USA; 188 University of Puerto Rico, Mayaguez, Puerto Rico USA; 189 Purdue University, West Lafayette, Indiana, USA; 190 Purdue University Northwest, Hammond, Indiana, USA; 191 Rice University, Houston, Texas USA; 192 University of Rochester, Rochester, New York, USA; 193 The Rockefeller University, New York, New York USA; 194 Rutgers, The State University of New Jersey, Piscataway, New Jersey USA; 195 University of Tennessee, Knoxville, Tennessee USA; 196 Texas A&M University, College Station, Texas USA; 197 Texas Tech University, Lubbock, Texas USA; 198 Vanderbilt University, Nashville, Tennessee USA; 199 University of Virginia, Charlottesville, Virginia USA; 200 Wayne State University, Detroit, Michigan USA; 201 University of Wisconsin -Madison, Madison, Wisconsin USA; 202 Present Address: Istanbul University, Istanbul, Turkey; 203 Yerevan Physics Institute, Yerevan, Armenia; 204 Present Address: University of Florida, Gainesville, Florida USA; 205 Imperial College, London, United Kingdom; 206 Present Address: University of Rochester, Rochester, New York USA; 207 Present Address: University of Cyprus, Nicosia, Cyprus; 208 Present Address: Baylor University, Waco, Texas USA; 209 Present Address: INFN Sezione di Torino, Università di Torino, Torino, Italy; 210 Università del Piemonte Orientale., Novara, Italy; 211 Institute of Nuclear Physics of the Uzbekistan Academy of Sciences, Tashkent, Uzbekistan; 212 TU Wien, Vienna, Austria; 213 Institute of Basic and Applied Sciences, Faculty of Engineering, Arab Academy for Science, Technology and Maritime Transport, Alexandria, Egypt; 214 Université Libre de Bruxelles, Bruxelles, Belgium; 215 Universidade Estadual de Campinas, Campinas, Brazil; 216 Federal University of Rio Grande do Sul, Porto Alegre, Brazil; 217 UFMS, Nova Andradina, Brazil; 218 The University of the State of Amazonas, Manaus, Brazil; 219 University of Chinese Academy of Sciences, Beijing, China; 220 Nanjing Normal University Department of Physics, Nanjing, China; 221 Present Address: The University of Iowa, Iowa City, Iowa USA; 222 University of Chinese Academy of Sciences., Beijing, China; 223 Helwan University, Cairo, Egypt; 224 Present Address: Zewail City of Science and Technology, Zewail, Egypt; 225 British University in Egypt, Cairo, Egypt; 226 Ain Shams University, Cairo, Egypt; 227 Purdue University, West Lafayette, Indiana USA; 228 Université de Haute Alsace, Mulhouse, France; 229 Department of Physics, Tsinghua University, Beijing, China; 230 Tbilisi State University, Tbilisi, Georgia; 231 Erzincan Binali Yildirim University, Erzincan, Turkey; 232 CERN, European Organization for Nuclear Research, Geneva, Switzerland; 233 University of Hamburg, Hamburg, Germany; 234 RWTH Aachen University, III. Physikalisches Institut A, Aachen, Germany; 235 Isfahan University of Technology, Isfahan, Iran; 236 Brandenburg University of Technology, Cottbus, Germany; 237 Forschungszentrum Jülich, Juelich, Germany; 238 Physics Department, Faculty of Science, Assiut University, Assiut, Egypt; 239 Karoly Robert Campus, MATE Institute of Technology, Gyongyos, Hungary; 240 Wigner Research Centre for Physics, Budapest, Hungary; 241 Institute of Physics, University of Debrecen, Debrecen, Hungary; 242 Institute of Nuclear Research ATOMKI, Debrecen, Hungary; 243 Present Address: Universitatea Babes-Bolyai -Facultatea de Fizica, Cluj-Napoca, Romania; 244 Faculty of Informatics, University of Debrecen, Debrecen, Hungary; 245 Punjab Agricultural University, Ludhiana, India; 246 UPES -University of Petroleum and Energy Studies, Dehradun, India; 247 University of Visva-Bharati, Santiniketan, India; 248 University of Hyderabad, Hyderabad, India; 249 Indian Institute of Science (IISc), Bangalore, India; 250 Indian Institute of Technology (IIT), Mumbai, India; 251 IIT Bhubaneswar, Bhubaneswar, India; 252 Institute of Physics, Bhubaneswar, India; 253 Deutsches Elektronen-Synchrotron, Hamburg, Germany; 254 Department of Physics, Isfahan University of Technology, Isfahan, Iran; 255 Sharif University of Technology, Tehran, Iran; 256 Department of Physics, University of Science and Technology of Mazandaran, Behshahr, Iran; 257 Italian National Agency for New Technologies, Energy and Sustainable Economic Development, Bologna, Italy; 258 Centro Siciliano di Fisica Nucleare e di Struttura Della Materia, Catania, Italy; 259 Scuola Superiore Meridionale, Università di Napoli ’Federico II’, Napoli, Italy; 260 Fermi National Accelerator Laboratory, Batavia, Illinois USA; 261 Università di Napoli ’Federico II’, Napoli, Italy; 262 Consiglio Nazionale delle Ricerche -Istituto Officina dei Materiali, Perugia, Italy; 263 Riga Technical University, Riga, Latvia; 264 Department of Applied Physics, Faculty of Science and Technology, Universiti Kebangsaan Malaysia, Bangi, Malaysia; 265 Consejo Nacional de Ciencia y Tecnología, Mexico City, Mexico; 266 IRFU, CEA, Université Paris-Saclay, Gif-sur-Yvette, France; 267 Faculty of Physics, University of Belgrade, Belgrade, Serbia; 268 Trincomalee Campus, Eastern University, Sri Lanka, Nilaveli, Sri Lanka; 269 INFN Sezione di Pavia, Università di Pavia, Pavia, Italy; 270 National and Kapodistrian University of Athens, Athens, Greece; 271 Ecole Polytechnique Fédérale Lausanne, Lausanne, Switzerland; 272 Universität Zürich, Zurich, Switzerland; 273 Stefan Meyer Institute for Subatomic Physics, Vienna, Austria; 274 Laboratoire d’Annecy-le-Vieux de Physique des Particules, IN2P3-CNRS, Annecy-le-Vieux, France; 275 Near East University, Research Center of Experimental Health Science, Nicosia, Turkey; 276 Konya Technical University, Konya, Turkey; 277 Piri Reis University, Istanbul, Turkey; 278 Adiyaman University, Adiyaman, Turkey; 279 Necmettin Erbakan University, Konya, Turkey; 280 Bozok Universitetesi Rektörlügü, Yozgat, Turkey; 281 Marmara University, Istanbul, Turkey; 282 Milli Savunma University, Istanbul, Turkey; 283 Kafkas University, Kars, Turkey; 284 Hacettepe University, Ankara, Turkey; 285 Istanbul University -Cerrahpasa, Faculty of Engineering, Istanbul, Turkey; 286 Ozyegin University, Istanbul, Turkey; 287 Vrije Universiteit Brussel, Brussel, Belgium; 288 School of Physics and Astronomy, University of Southampton, Southampton, United Kingdom; 289 University of Bristol, Bristol, United Kingdom; 290 IPPP Durham University, Durham, United Kingdom; 291 Monash University, Faculty of Science, Clayton, Australia; 292 Università di Torino, Torino, Italy; 293 Bethel University, St. Paul, Minnesota, USA; 294 Karamanoğlu Mehmetbey University, Karaman, Turkey; 295 University of Science and Technology of China, Hefei, China; 296 United States Naval Academy, Annapolis, Maryland, USA; 297 Bingol University, Bingol, Turkey; 298 Georgian Technical University, Tbilisi, Georgia; 299 Sinop University, Sinop, Turkey; 300 Erciyes University, Kayseri, Turkey; 301 Institute of Modern Physics and Key Laboratory of Nuclear Physics and Ion-beam Application (MOE) -Fudan University, Shanghai, China; 302 Texas A&M University at Qatar, Doha, Qatar; 303 Kyungpook National University, Daegu, Korea

**Keywords:** Experimental particle physics

## Abstract

In July 2012, the ATLAS and CMS collaborations at the CERN Large Hadron Collider announced the observation of a Higgs boson at a mass of around 125 gigaelectronvolts. Ten years later, and with the data corresponding to the production of a 30-times larger number of Higgs bosons, we have learnt much more about the properties of the Higgs boson. The CMS experiment has observed the Higgs boson in numerous fermionic and bosonic decay channels, established its spin–parity quantum numbers, determined its mass and measured its production cross-sections in various modes. Here the CMS Collaboration reports the most up-to-date combination of results on the properties of the Higgs boson, including the most stringent limit on the cross-section for the production of a pair of Higgs bosons, on the basis of data from proton–proton collisions at a centre-of-mass energy of 13 teraelectronvolts. Within the uncertainties, all these observations are compatible with the predictions of the standard model of elementary particle physics. Much evidence points to the fact that the standard model is a low-energy approximation of a more comprehensive theory. Several of the standard model issues originate in the sector of Higgs boson physics. An order of magnitude larger number of Higgs bosons, expected to be examined over the next 15 years, will help deepen our understanding of this crucial sector.

## Abstract (web summary)

The most up-to-date combination of results on the properties of the Higgs boson is reported, which indicate that its properties are consistent with the standard model predictions, within the precision achieved to date.

## Main

The established theory of elementary particle physics, commonly referred to as the standard model (SM), provides a complete description of the electromagnetic, weak and strong interactions of matter particles, which are spin-1/2 fermions, through three different sets of mediators, which are spin-1 bosons. (In quantum mechanics, spin is an intrinsic form of angular momentum carried by elementary particles). These vector bosons are the massless photons (gluons) for the electromagnetic (strong) interaction, and the heavy W and Z bosons for the weak interaction. The SM has been very successful in providing accurate predictions for essentially all particle physics experiments carried out so far. In 2012, the final missing particle of the SM, the Higgs boson, was observed by the ATLAS^1^ and CMS^2,3^ collaborations at CERN.

The Higgs boson is a prediction of a mechanism that took place in the early Universe, less than a picosecond after the Big Bang, which led to the electromagnetic and the weak interactions becoming distinct in their actions. In the SM, this mechanism, labelled as the Brout–Englert–Higgs (BEH) mechanism, introduces a complex scalar (spin-0) field that permeates the entire Universe. Its quantum manifestation is known as the SM Higgs boson. Scalar fields are described only by a number at every point in space that is invariant under Lorentz transformations. An analogy can be drawn of a map of an area where temperature is shown at various positions mimicking a scalar field. The same map, where instead the wind speed and direction are shown, would correspond to a vector field.

## The long road to the Higgs boson

The BEH mechanism was first proposed in 1964 in the works of Brout and Englert^4^, Higgs^5,6^, and Guralnik, Hagen and Kibble^7^. Further details of the mechanism were presented in 1966 by Higgs^8^ and in 1967 by Kibble^9^. In 1967, Weinberg^10^ and Salam^11^, extending the 1961 work of Glashow^12^, proposed the use of the BEH mechanism for a theory of the unification of the electromagnetic and weak interactions, labelled as the electroweak interaction. The key element in this work was the conjecture that nature possesses an electroweak symmetry, mathematically described by the Lagrangian of the theory, which is spontaneously broken, granting mass to the W and Z bosons. An additional feature of this model is that it provides a mechanism for granting masses to fermions as well, through the so-called Yukawa interactions^10,13^. Thus, the elementary particles interacting with the BEH field acquire mass. The impact is far reaching: for example, electrons become massive, allowing atoms to form, and endowing our Universe with the observed complexity.

Salam and Weinberg had further conjectured that the model they put forward might be renormalizable (that is, give finite answers). In 1971, ’t Hooft and Veltman^14,15^ showed how indeed this theory could be renormalized. This development put the Glashow–Salam–Weinberg model on a firm basis deserving serious experimental scrutiny.

After the W and Z bosons were discovered by the UA1 and UA2 experiments at CERN in 1983^16–19^, the search for the Higgs boson became a central thrust in particle physics and an important motivation for the CERN Large Hadron Collider (LHC)^20^, and the ATLAS and CMS experiments.

Finding the Higgs boson has been demanding. This is a consequence of its large mass, which puts it beyond the reach of previous electron–positron colliders, such as the Large Electron–Positron (LEP) collider^21^ at CERN, and low cross-section modes coupled with unfavourable decay channels in the range of mass in which it was eventually found, which made it challenging to observe at previous hadron colliders, such as the Tevatron^22^ at Fermilab. In the SM, the Higgs boson is an elementary scalar particle, a type that had never been observed before. Fundamental scalar particles are subject to quantum corrections that can be as large as the scale of the physics beyond the SM (BSM). As this scale can be many orders of magnitude larger than the electroweak scale, which is about 100 GeV, the measured mass of the Higgs boson is puzzlingly small. How to resolve this puzzle is part of the motivation for future work and accelerators.

The BEH mechanism does not predict the mass of the Higgs boson, but once the mass is fixed, all its other properties are precisely defined. The Higgs boson, once produced, decays directly to the heaviest allowed elementary particles. However, decays to massless particles can also occur through quantum loops. At the LHC, the production of Higgs bosons is dominated by gluon–gluon fusion (ggH) proceeding via a virtual top quark loop. The mass of a real particle is defined as *m*^2^ = *E*^2^ − *p*^2^, where *E* is the energy and **p** is the momentum vector of the particle. For a virtual particle, this equation is not valid and thus a virtual particle does not have a defined value of the mass. A virtual particle is denoted by an asterisk, for example, W* denoting a virtual W boson. Henceforth the distinction between real and virtual particles will be dropped, unless mentioned otherwise. At a mass of around 125 GeV, the Higgs boson decays dominantly into a b quark and its antiquark. Henceforth, the distinction between a particle and its antiparticle will be dropped.

From the accurate observation and measurement of the products of the Higgs boson decays and of those associated with its production, experiments are able to infer its properties, including the strength of its self-interaction (*λ*)^23^ and, potentially, decays into BSM particles.

This paper presents the combination of results from single Higgs boson production and decay, and its pair production, using datasets corresponding to an integrated luminosity $( {\mathcal L} )$ up to 138 fb^−1^ (ref. ^24^), collected by the CMS in 2016–2018. An integrated luminosity of 1 fb^−1^ corresponds to about 100 trillion proton–proton collisions at a centre-of-mass energy of 13 TeV.

In addition, a few projections are made for an assumed data sample corresponding to ${\mathcal{L}}=3,000\,{{\rm{fb}}}^{-1}$, recorded at $\sqrt{s}=14\,{\rm{TeV}}$, expected to be accumulated by the end of the next decade during the high-luminosity operation of the LHC accelerator (HL-LHC).

## The CMS experiment and datasets

**Extended Data Fig. 1:**
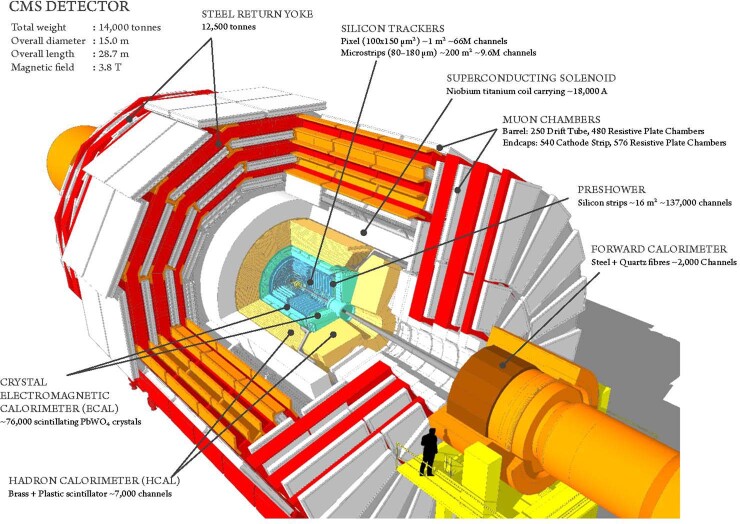
The CMS detector at the CERN LHC. Schematic longitudinal cut-away view of the CMS detector, showing the different layers around the LHC beam axis, with the collision point in the centre.

The CMS apparatus^25^, illustrated in Extended Data Fig. 1, is a multipurpose, nearly hermetic detector, designed to trigger on^26,27^ and identify electrons (e), muons (μ), photons (γ) and (charged and neutral) hadrons^28–30^. A trigger is a filter that selects interesting events, where ‘event’ refers to the result of the selected interaction in a beam crossing, as observed in the detector. A global event reconstruction algorithm^31^ combines the information provided by the all-silicon inner tracker, crystal electromagnetic calorimeter, and brass and scintillator hadron calorimeters, operating inside a 3.8-T superconducting solenoid, with data from gas-ionization muon detectors embedded in the solenoid flux-return yoke, to build electrons, muons, tau (τ) leptons, photons, hadronic jets, missing transverse momentum $({p}_{{\rm{T}}}^{{\rm{miss}}})$ and other physics objects^32–34^. Collimated streams of particles arising from the fragmentation of quarks or gluons are called ‘jets’. These jets are identified, and their energies measured, by specialized reconstruction algorithms^31,33^. The missing transverse momentum vector is measured with respect to the incoming proton beams, and it is computed as the negative vector sum of transverse momenta of all particles in an event.

Several improvements have been introduced into the CMS experiment since the discovery of the Higgs boson in 2012 (Methods).

By July 2012, CMS had collected data corresponding to ${\mathcal{L}}=5.1\,{{\rm{fb}}}^{-1}$ at a proton–proton (pp) collision centre-of-mass energy $\sqrt{s}=7\,{\rm{TeV}}$ (in 2011) and ${\mathcal{L}}=5.3\,{{\rm{fb}}}^{-1}$ at $\sqrt{s}=8\,{\rm{TeV}}$ (in the first half of 2012), with which the Higgs boson was discovered. By the end of 2012 (Run 1), CMS had collected data corresponding to ${\mathcal{L}}=19.7\,{{\rm{fb}}}^{-1}$ at $\sqrt{s}=8\,{\rm{TeV}}$ (ref. ^35^).

In LHC Run 2 (2015–2018), the accelerator delivered collisions at $\sqrt{s}=13\,{\rm{TeV}}$. At this larger energy, the cross-section for Higgs boson production increases by a factor of 2.2–4.0, depending on the production mode^36–39^. Physics analyses presented here are based on 2016–2018 data, corresponding to ${\mathcal L}$ of up to 138 fb^−1^ (the additional approximately 2 fb^−1^ recorded in 2015 are not used in this combination). This enabled a reduction of not only statistical but also systematic uncertainties, as well as a more precise calibration of the calorimeters and alignment of the tracking detectors. During Run 2, approximately 8 million Higgs bosons were produced. Many more final states could be studied, as it was possible to separate the events by production mode and decay channel, as well as by kinematic properties; and differential distributions could be measured. Furthermore, improved analysis methods were deployed.

To enable comparison with the more precise experimental results, theoretical calculations have been carried out with commensurate improvements in accuracy^36–39^, involving higher orders in perturbation theory.

The statistical procedure was developed in preparation for the search and discovery of the Higgs boson and has not changed much since then. It is based on building a combined likelihood from the various input channels (‘Statistical analysis’ in Methods). Parameter estimation and limit setting are performed using a profile likelihood technique with asymptotic approximation^40^, taking into account the full correlation of the systematic uncertainties between individual channels and the years of data taking. The different channels included in the combination correlate nuisance parameters related to the same underlying effect, such as the uncertainty in the theoretical prediction or the energy-scale uncertainty of the final-state objects. The inclusive signal strength (*μ*) combination has a total of ${\mathscr{O}}({10}^{4})$ nuisance parameters. The references to the individual analyses presented in the next section each contain more details of the statistical procedure used for combining the several categories used, created according to various criteria, such as signal-to-background ratios, mass resolutions and multiplicities of physics objects.

## Portrait of the Higgs boson

The portrait of the Higgs boson is defined by its production modes, via cross-sections, and its decay channels, via branching fractions. For the value of mass measured by CMS *m*_H_ = 125.38 ± 0.14 GeV (ref. ^41^), these are given in Extended Data Table 1^39^.

### Production

**Fig. 1:**
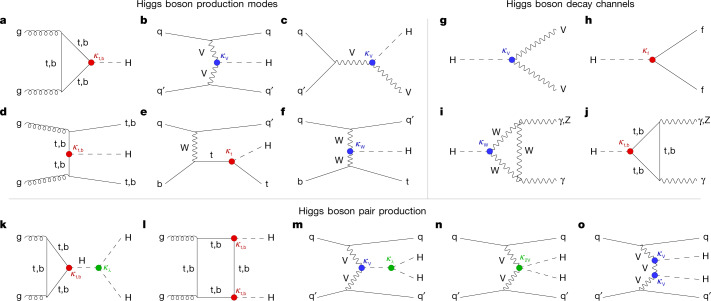
Feynman diagrams for the leading Higgs boson interactions. **a**–**f**, Higgs boson production in ggH (**a**) and VBF (**b**), associated production with a W or Z (V) boson (VH; **c**), associated production with a top or bottom quark pair (ttH or bbH; **d**) and associated production with a single top quark (tH; **e**,**f**). **g**–**j**, Higgs boson decays into heavy vector boson pairs (**g**), fermion–antifermion pairs (**h**) and photon pairs or Zγ (**i**,**j**). **k**–**o**, Higgs boson pair production through ggH (**k**,**l**) and through VBF (**m**,**n**,**o**). The different Higgs boson interactions are labelled with the coupling modifiers *κ*, and highlighted in different colours for Higgs–fermion interactions (red), Higgs–gauge-boson interactions (blue) and multiple Higgs boson interactions (green). The distinction between a particle and its antiparticle is dropped.

The rate of production of Higgs bosons is given by the product of the instantaneous luminosity, measured in units of cm^−2^ s^−1^, and the cross-section, measured in units of cm^2^. For *m*_H_ = 125.38 GeV, the total cross-section for the production of the SM Higgs boson at $\sqrt{s}=13\,{\rm{TeV}}$ is 54 ± 2.6 pb (ref. ^39^). (A cross-section of 1 pb (picobarn) corresponds to an area of 10^−36^ cm^2^). This results in the production of one Higgs boson every second at an instantaneous luminosity of 2 × 10^34^ cm^−2^ s^−1^. The dominant production mode in the SM is ggH, where a pair of gluons, one from each of the incident protons, fuses, predominantly via a virtual top quark quantum loop. This is depicted in Fig. 1a and represents 87% of the total cross-section. The next most important production mode is vector boson fusion (VBF) depicted in Fig. 1b, where a quark from each of the protons radiates a virtual vector boson (W or Z), which then fuse together to make a Higgs boson. Other processes, with smaller cross-sections, are: production in association with a vector boson or ‘Higgsstrahlung’ (VH) depicted in Fig. 1c, and production in association with top (tH and ttH) or bottom (bbH) quarks, depicted in Fig. 1d–f. The bbH mode has not been studied in the context of the SM Higgs boson because of limited sensitivity.

Events are categorized according to the signatures particular to each production mechanism. For example, they are categorized as VBF-produced if there are two high transverse momentum (*p*_T_) jets, or as VH-produced if there are additional charged leptons (${\ell }$) and/or ${p}_{{\rm{T}}}^{{\rm{miss}}}$, or ttH- and tH-produced if there are jets identified as coming from b quarks, or otherwise ggH-produced. (The top quark predominantly decays into a W boson and a b-quark jet).

### Decays

In the SM, particle masses arise from spontaneous breaking of the gauge symmetry, through gauge couplings to the Higgs field in the case of vector bosons, and Yukawa couplings in the case of fermions. The SM Higgs boson couples to vector bosons, with an amplitude proportional to the gauge boson mass squared ${m}_{{\rm{V}}}^{2}$, and to fermions with an amplitude proportional to the fermion mass *m*_f_. Hence, for example, the coupling is stronger for the third generation of quarks and leptons than for those in the second generation. The observation of many Higgs boson decays to SM particles and the measurement of their branching fractions are a crucial test of the validity of the theory. Any sizeable deviation from the predictions could indicate the presence of BSM physics.

The Higgs boson, once produced, rapidly decays into a pair of fermions or a pair of bosons. In the SM, its lifetime is ${\tau }_{{\rm{H}}}\approx 1.6\times {10}^{-22}\,{\rm{s}}$, and its inverse, the natural width, is $\varGamma =\hbar /{\tau }_{{\rm{H}}}=4.14\pm 0.02\,{\rm{MeV}}$ (ref. ^39^), where *ħ* is the reduced Planck’s constant. The natural width is the sum of all the partial widths, and the ratios of the partial widths to the total width are called branching fractions and represent the probabilities for that decay channel to occur. The Higgs boson does not couple directly to massless particles (for example, the gluon or the photon), but can do so through quantum loops (for example, Fig. 1a,i,j).

By design, the event selections do not overlap among analyses targeting different final states. Where the final states are similar, the overlap has been checked and found to be negligible.

Detailed information on the analyses included in the new combination along with improvements, and the online and offline criteria used to select events for the analyses can be found in Methods, Extended Data Tables 2 and 3, and the associated references. Online reconstruction is performed in real time as the data are being collected. Offline reconstruction is performed later on stored data. The background-subtracted distributions of the invariant mass of final-state particles in the individual decay channels are shown in Extended Data Figs. 3 and 4. The channels that are used in this combination are as follows.

Bosonic decay channels: H → γγ (Fig. 1i, j)^42^; H → ZZ → 4${\ell }$ (Fig. 1g)^43^; H → WW → ${\ell }$ν${\ell }$v (Fig. 1g)^44^, H → Zγ (Fig. 1i, j)^45^; fermionic decay channels: H → ττ, third-generation fermion (Fig. 1h)^46^, H → bb, third-generation fermion (Fig. 1h)^47–51^, H → μμ, second-generation fermion (Fig. 1h)^52^; ttH and tH with multileptons (Fig. 1d–f)^53^; Higgs boson decays beyond the SM^35^.

## Higgs boson pair production

The measurement of the pair production of Higgs bosons can probe its self-interaction *λ*. The pair production modes are shown in Fig. 1k–o.

In the ggH mode, there are two leading contributions: in the first (Fig. 1l), two Higgs bosons emerge from a top or bottom quark loop; in the second (Fig. 1k), a single virtual Higgs boson, H*, emerges from the top or bottom quark loop and then decays to two Higgs bosons $({\rm{gg}}\to {{\rm{H}}}^{* }\to {\rm{HH}}).$ Explicit establishment of the latter contribution, a direct manifestation of the Higgs boson’s self-interaction, would elucidate the strikingly unusual potential of the BEH field.

**Fig. 2:**
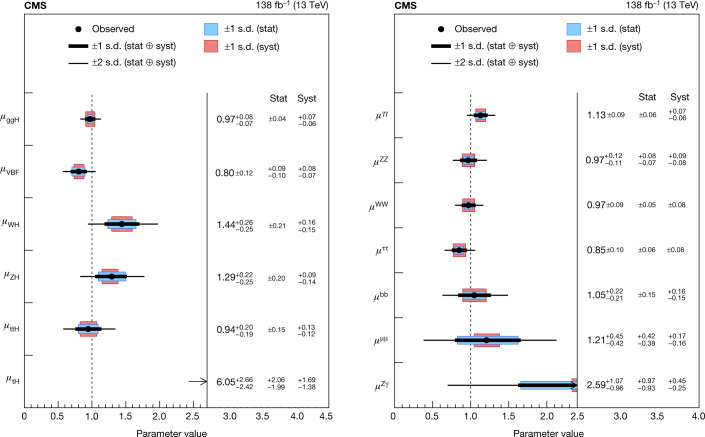
The agreement with the SM predictions for production modes and decay channels. Signal-strength parameters extracted for various production modes *μ*_*i*_, assuming ${ {\mathcal B} }^{f}={({ {\mathcal B} }^{f})}_{{\rm{SM}}}$ (left), and decay channels *μ*^*f*^, assuming *σ*_*i*_ = (*σ*_*i*_)_SM_ (right). The thick and thin black lines indicate the 1-s.d. and 2-s.d. confidence intervals, respectively, with the systematic (syst) and statistical (stat) components of the 1-s.d. interval indicated by the red and blue bands, respectively. The vertical dashed line at unity represents the values of *μ*_*i*_ and *μ*^*f*^ in the SM. The covariance matrices of the fitted signal-strength parameters are shown in Extended Data Fig. 5. The *P* values with respect to the SM prediction are 3.1% and 30.1% for the left plot and the right plot, respectively. The *P* value corresponds to the probability that a result deviates as much, or more, from the SM prediction as the observed one.

In the VBF mode, there are three subprocesses that can lead to production of a pair of Higgs bosons: (1) through a virtual Higgs boson (Fig. 1m); (2) through a four-point interaction: VV → HH (Fig. 1n); and (3) through the exchange of a vector boson (Fig. 1o).

The scattering amplitudes of the processes giving rise to Higgs boson pair production through ggH (Fig. 1k,l) are similar in magnitude, but have opposite signs and interfere destructively. This makes the overall Higgs boson pair production rate small, rendering its experimental observation challenging. The SM Higgs boson pair production cross-section is calculated for *m*_H_ = 125 GeV to be ${32.76}_{-6.83}^{+1.95}\,{\rm{fb}}$ (refs. ^54–56^), three orders of magnitude smaller than the single Higgs boson cross-section.

The search for Higgs boson pair production is performed by combining Higgs boson candidates reconstructed from different final states^57–62^. All final states analysed are defined to be mutually exclusive so that they could be combined as statistically independent observations.

## Measurement of the properties of the Higgs boson

At the time of the Higgs boson discovery^2,3^, the combination of CMS data gave an observed (obs.) statistical significance of 5.0 standard deviation (s.d.) with an expected (exp.) significance of 5.8 s.d. Individually, the most sensitive channels, H → γγ and H → ZZ → 4${\ell }$, yielded 4.1 s.d. obs. (2.8 s.d exp.) and 3.2 s.d. obs. (3.8 s.d. exp.), respectively.

Using all the Run 1 data, it was possible to observe separately the bosonic decay channels with significances of 6.5 s.d for H → ZZ → 4${\ell }$, 5.6 s.d. for H → γγ, 4.7 s.d. for H → WW and 3.8 s.d. for the fermionic decay channel H → ττ (ref. ^35^). Earlier, the first results of the Higgs boson decay into fermions were presented in ref. ^63^, reaching a significance of 3.8 s.d by combining the H → ττ and H → bb decay modes. The mass was measured to a precision of about 0.2% (ref. ^35^). Using the angular distributions of the leptons in the bosonic decay channels, the spin (*J*) and parity (*P*, a parity transformation that effectively turns a phenomenon into its mirror image) were also found to be compatible with the SM prediction (*J*^*P*^ = 0^+^) with a large number of alternative spin–parity hypotheses ruled out at the >99.9% confidence level (CL)^64,65^. The total cross-section, combining all of the different decay channels, was measured to be in agreement with the SM, with an uncertainty of 14% (ref. ^35^). Each of the VBF, VH and ttH production modes was measured at a level of 3 s.d. (ref. ^35^).

With the Run 2 data, CMS has observed the Higgs boson decaying into a pair of τ leptons with a significance of 5.9 s.d. (ref. ^66^), a pair of bottom quarks with a significance of 5.6 s.d. (ref. ^48^) and the ttH production mode at 5.2 s.d. (ref. ^67^). The Higgs boson has also been seen in its decays into muons with a significance of 3 s.d. (ref. ^52^). The mass of the Higgs boson has been measured to be 125.38 ± 0.14 GeV using the decay channels H → γγ and ${\rm{H}}\to {\rm{ZZ}}\to 4{\ell }$ (ref. ^41^). The natural width of the Higgs boson has been extracted and is found to be ${\varGamma }_{{\rm{H}}}={3.2}_{-1.7}^{+2.4}\,{\rm{MeV}}$ by using off-mass-shell and on-mass-shell Higgs boson production^68^. On-mass-shell refers to a particle with its physical mass, and off-mass-shell refers to a virtual particle.

### The *μ* framework for signal strengths

The agreement between the observed signal yields and the SM expectations can be quantified by fitting the data with a model that introduces signal-strength parameters. These are generically labelled *μ*, and scale the observed yields with respect to those predicted by the SM, without altering the shape of the distributions. The specific meaning of *μ* varies depending on the analysis. For given initial (*i*) and final (*f*) states, *i* → H → *f*, the signal strengths for individual production channels, *μ*_*i*_, and decay modes, *μ*^f^, are defined as *μ*_*i*_ = *σ*_*i*_/(*σ*_*i*_)_SM_ and ${\mu }^{f}={ {\mathcal B} }^{f}/{({ {\mathcal B} }^{f})}_{{\rm{SM}}}$, where *σ* is the production cross-section and ${\mathcal B}$ is the branching fraction. Perfect agreement with SM expectations would yield all *μ* equal to one.

A first test of compatibility is performed by fitting all data from production modes and decay channels with a common signal-strength parameter, *μ*. At the time of discovery, the common *μ* was found to be 0.87 ± 0.23. The new combination of all the Run 2 data yields *μ* = 1.002 ± 0.057, in excellent agreement with the SM expectation. The uncertainties in the new measurement correspond to an improvement by a factor of 4.5 in precision compared with what was achieved at the time of discovery. At present, the theoretical uncertainties in the signal prediction, and the experimental statistical and the systematic uncertainties separately contribute at a similar level, and they are 0.036, 0.029 and 0.033, respectively.

Relaxing the assumption of a common signal-strength parameter, and introducing different *μ*_*i*_ and *μ*^*f*^, our measurements are shown in Fig. 2. The production modes ggH, VBF, WH, ZH and ttH are all observed with a significance of 5 s.d. or larger.

### The *κ* framework for coupling modifiers

BSM physics is expected to affect the production modes and decay channels in a correlated way if they are governed by similar interactions. Any modification in the interaction between the Higgs boson and, for example, the W bosons and top quarks would affect not only the H → WW (Fig. 1g) or H → γγ (Fig. 1i,j) decay rates but also the production cross-section for the ggH (Fig. 1a), WH (Fig. 1c) and VBF (Fig. 1b) modes. To probe such deviations from the predictions of the SM, the *κ* framework^38^ is used. The quantities, such as *σ*_*i*_, *Γ*^*f*^ and *Γ*_H_, computed from the corresponding SM predictions, are scaled by ${\kappa }_{i}^{2}$, as indicated by the vertex labels in Fig. 1. As an example, for the decay H → γγ proceeding via the loop processes of Fig. 1i,j, the branching fraction is proportional to ${\kappa }_{{\rm{\gamma }}}^{2}$ or ${(1.26{\kappa }_{{\rm{W}}}-0.26{\kappa }_{{\rm{t}}})}^{2}$. In the SM, all *κ* values are equal to one.

**Fig. 3:**
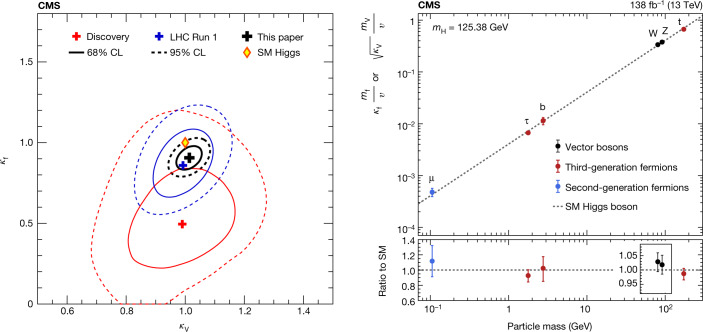
A portrait of the Higgs boson couplings to fermions and vector bosons. Left: constraints on the Higgs boson coupling modifiers to fermions (*κ*_f_) and heavy gauge bosons (*κ*_V_), in different datasets: discovery (red), the full LHC Run 1 (blue) and the data presented here (black). The SM prediction corresponds to *κ*_V_ = *κ*_f_ = 1 (diamond marker). Right: the measured coupling modifiers of the Higgs boson to fermions and heavy gauge bosons, as functions of fermion or gauge boson mass, where *υ* is the vacuum expectation value of the BEH field (‘Notes on self-interaction strength’ in Methods). For gauge bosons, the square root of the coupling modifier is plotted, to keep a linear proportionality to the mass, as predicted in the SM. The *P* value with respect to the SM prediction for the right plot is 37.5%.

A first such fit to Higgs boson couplings introduces two parameters, *κ*_V_ and *κ*_f_, scaling the Higgs boson couplings to massive gauge bosons and to fermions, respectively. With the limited dataset available at the time of discovery, such a fit provided first indications for the existence of both kinds of coupling. The sensitivity with the present data is much improved, and both coupling modifiers are measured to be in agreement, within an uncertainty of 10%, with the predictions from the SM, as shown in Fig. 3 (left).

A second fit is performed to extract the coupling modifiers *κ* for the heavy gauge bosons (*κ*_W_ and *κ*_Z_) and the fermions probed in the present analyses (*κ*_t_, *κ*_b_, *κ*_τ_ and *κ*_μ_). Predictions for processes that in the SM occur via loops of intermediate virtual particles, for example, Higgs boson production via ggH, or Higgs boson decay to a pair of gluons, photons or Zγ, are computed in terms of the *κ*_*i*_ above. The result is shown in Fig. 3 (right), as a function of the mass of the probed particles. The remarkable agreement with the predictions of the BEH mechanism over three orders of magnitude of mass is a powerful test of the validity of the underlying physics. Statistical and systematic uncertainties contribute at the same level to all measurements, except for *κ*_μ_, which still is dominated by the statistical uncertainty.

**Fig. 4:**
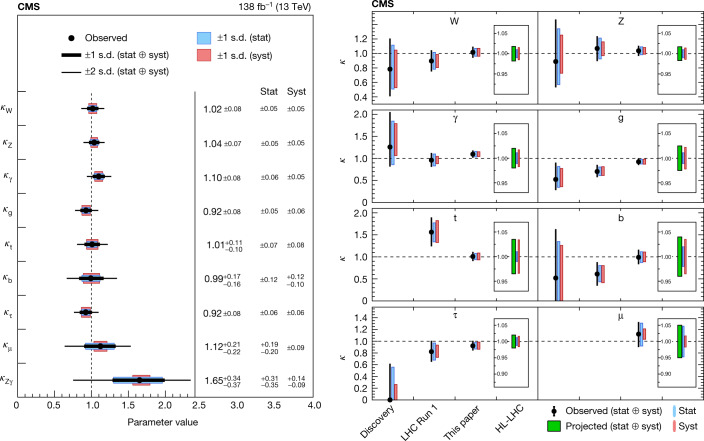
Coupling modifier measurements and their evolution in time. Left: coupling modifiers resulting from the fit. The *P* value with respect to the SM prediction is 28%. Right: observed and projected values resulting from the fit in the *κ* framework in different datasets: at the time of the Higgs boson discovery, using the full data from LHC Run 1, in the dataset used in this paper and the expected 1-s.d. uncertainty at the HL-LHC for ${\mathcal{L}}=3,000\,{{\rm{fb}}}^{-1}$. The H → μμ and *κ*_t_ measurements were not available for earlier datasets owing to the lack of sensitivity.

In extensions of the SM with new particles, the loop-induced processes may receive additional contributions. A more general fit for deviations in the Higgs boson couplings can then be defined by introducing additional modifiers for the effective coupling of the Higgs boson to gluons (*κ*_g_), photons (*κ*_γ_) and Zγ (*κ*_Zγ_). The results for this fit are shown in Fig. 4 (left). Coupling modifiers are probed at a level of uncertainty of 10%, except for *κ*_b_ and *κ*_μ_ (about 20%) and *κ*_Zγ_ (about 40%), and all measured values are compatible with the SM expectations, to within 1.5 s.d. These measurements correspond to an increase in precision by a factor of about five compared with what was possible with the discovery dataset. Figure 4 (right) and Extended Data Fig. 8 (left) illustrate the evolution of several *κ* measurements and their uncertainties using the dataset: at the time of discovery (July 2012)^2,3^; for the full Run 1 (end of 2012)^35^; for results presented in this paper; and expected to be accumulated by the end of the HL-LHC running^69^, corresponding to ${\mathcal{L}}=3,000\,{{\rm{fb}}}^{-1}$. The statistical uncertainties have been scaled by $1/\sqrt{ {\mathcal L} }$, the experimental systematic ones by $1/\sqrt{ {\mathcal L} }$ where possible, or fixed at values suggested in ref. ^69^, whereas the theoretical uncertainties have been halved.

A sizeable improvement is expected after HL-LHC operation. The H → μμ measurements were not available for the first two datasets owing to the lack of sensitivity. The evolution of several signal-strength measurements *μ* are shown in Extended Data Fig. 7.

If new particles exist with masses smaller than *m*_H_, other decay channels may be open. Examples of such decays could be into new neutral long-lived particles or into dark-matter particles, neither leaving a trace in the CMS detector. We refer to these as ‘invisible’ Higgs boson decays, which could be inferred from the presence of large ${p}_{{\rm{T}}}^{{\rm{miss}}}$ in the direction of the Higgs boson momentum. The events are selected based on other particles accompanying the Higgs boson. Dedicated searches for such decays^70–72^ yielded ${ {\mathcal B} }_{{\rm{Inv}}.} < 0.16$ at 95% CL, where ${ {\mathcal B} }_{{\rm{Inv}}.}$ is the branching fraction to invisible decays.

## Results from the search for Higgs boson pair production

**Fig. 5:**
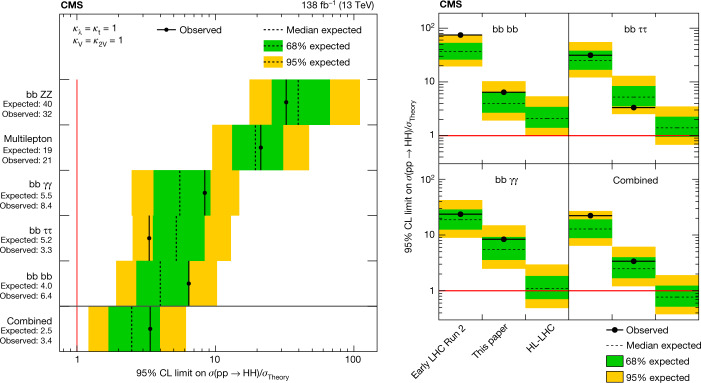
Limits on the production of Higgs boson pairs and their time evolution. Left: the expected and observed limits on the ratio of experimentally estimated production cross-section and the expectation from the SM (*σ*_Theory_) in searches using different final states and their combination. The search modes are ordered, from upper to lower, by their expected sensitivities from the least to the most sensitive. The overall combination of all searches is shown by the lowest entry. Right: expected and observed limits on HH production in different datasets: early LHC Run 2 data (35.9 fb^−1^), present results using full LHC Run 2 data (138 fb^−1^) and projections for the HL-LHC (3,000 fb^−1^).

The cross-section for Higgs boson pair production in the SM is extremely small, thus escaping detection at the LHC so far. The results of the search are therefore expressed as an upper limit on the production cross-section. Figure 5 (left) shows the expected and observed limits on Higgs boson pair production, expressed as ratios with respect to the SM expectation, in searches using the different final states and their combination. With the current dataset, and combining data from all currently studied modes and channels, the Higgs boson pair production cross-section is found to be less than 3.4 times the SM expectation at 95% CL. Figure 5 (right) shows the evolution of the limits from the three most sensitive modes and the overall combination for: the first comprehensive set of measurements using early LHC Run 2 data (35.9 fb^−1^)^73^, the present measurements using the full LHC Run 2 data (138 fb^−1^) and the projections for the HL-LHC (3,000 fb^−1^)^69^. The HL-LHC projections are also expressed as limits, assuming that there is no Higgs boson pair production. The fact that the combined limit is expected to be below unity shows that the sensitivity is sufficient to establish the existence of the SM HH production.

**Fig. 6:**
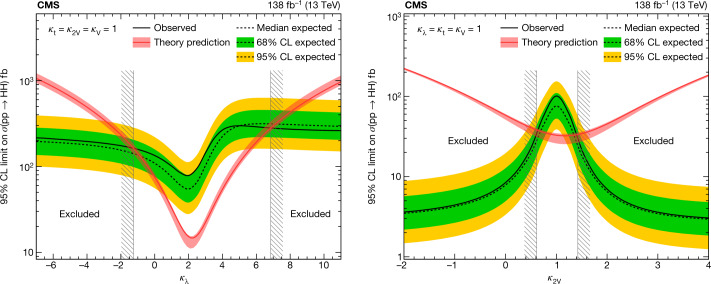
Limits on the Higgs boson self-interaction and quartic coupling. Combined expected and observed 95% CL upper limits on the HH production cross-section for different values of *κ*_λ_ (left) and *κ*_2V_ (right), assuming the SM values for the modifiers of Higgs boson couplings to top quarks and vector bosons. The green and yellow bands represent the 1-s.d. and 2-s.d. extensions beyond the expected limit, respectively; the red solid line (band) shows the theoretical prediction for the HH production cross-section (its 1-s.d. uncertainty). The areas to the left and to the right of the hatched regions are excluded at the 95% CL.

Figure 6 presents the expected and observed experimental limits on the HH production cross-section as functions of the Higgs boson self-interaction coupling modifier *κ*_λ_ and the quartic VVHH coupling modifier *κ*_2V_. Cross-section values above the solid black lines are experimentally excluded at 95% CL. The red lines show the predicted cross-sections as functions of *κ*_λ_ or *κ*_2V_, which exhibit a characteristic dip in the vicinity of the SM values (*κ* = 1) owing to the destructive interference of the contributing production amplitudes, as highlighted in ‘Higgs boson pair production’. The experimental limits on the Higgs boson pair production cross-section (black lines) also show a strong dependence on the assumed values of *κ*. This is because the interference between different subprocesses, besides changing the expected cross-sections, also changes the differential kinematic properties of the two Higgs bosons, which in turn affects strongly the efficiency for detecting signal events. With the current dataset, we can ascertain at the 95% CL that the Higgs boson self-interaction coupling modifier *κ*_λ_ is in the range of −1.24 to 6.49, whereas the quartic *κ*_2V_ coupling modifier is in the range of 0.67 to 1.38. Figure 6 (right) shows that *κ*_2V_ = 0 is excluded, with a significance of 6.6 s.d., establishing the existence of the quartic coupling VVHH depicted in Fig. 1n.

## Current knowledge and future prospects

The discovery of the Higgs boson in 2012 completed the particle content of the SM of elementary particle physics, a theory that explains visible matter and its interactions in exquisite detail. The completion of the SM spanned 60 years of theoretical and experimental work. In the ten years following the discovery, great progress has been made in painting a clearer portrait of the Higgs boson.

In this paper, the CMS Collaboration reports the most up-to-date combination of results on the properties of the Higgs boson, based on data corresponding to an ${\mathcal L}$ of up to 138 fb^−1^, recorded at 13 TeV. Many of its properties have been determined with accuracies better than 10%. All measurements made so far are found to be consistent with the expectations of the SM. In particular, the overall signal-strength parameter has been measured to be *μ* = 1.002 ± 0.057. It has been shown that the Higgs boson directly couples to bottom quarks, tau leptons and muons, which had not been observed at the time of the discovery, and also proven that it is indeed a scalar particle. The CMS experiment is approaching the sensitivity necessary to probe Higgs boson couplings to charm quarks^74^. The observed (expected) 95% CL value for *κ*_c_ is found to be $1.1 < |{\kappa }_{{\rm{c}}}| < 5.5$ ($| {\kappa }_{{\rm{c}}}|  < 3.40$), the most stringent result so far. Moreover, the recent progress in searches for the pair production of Higgs bosons has allowed the setting of tight constraints on the Higgs boson self-interaction strength, and the setting of limits on the Higgs boson pair production cross-section not much above twice the expected SM value.

Much evidence points to the fact that the SM is a low-energy approximation of a more comprehensive theory. In connection with the mechanism of spontaneous symmetry breaking, several puzzles appear: the so-called naturalness, a technical issue related to the fact that the Higgs boson mass is close to the electroweak scale; in relation to cosmology, the metastability of the vacuum state of the SM and the conjectured period of inflation in the early Universe; the dynamics of the electroweak phase transition and its connection to the matter–antimatter asymmetry of our Universe. These issues motivate attempts at obtaining a deeper understanding of the physics of the Higgs boson. The impressive progress made over the past decade is foreseen to continue into the next one. The current dataset is expected to be doubled in size by the middle of this decade, enabling the establishment of rare decays channels such as H → μμ and H → Zγ. Operation with the high-luminosity LHC is expected during the next decade and should yield ten times more data then originally foreseen. This should allow the ATLAS and CMS experiments to establish the SM Higgs boson pair production with a significance of 4 s.d., as well as the Higgs boson coupling to charm quarks, and to search for any exotic decays. Improvements in experimental techniques and theoretical calculations are also anticipated to continue. The CMS experiment is entering the era of precision Higgs physics that will shed light on BSM physics.

## Methods

### LHC project and the Higgs boson

The primary goals of the LHC and its two general-purpose experiments, ATLAS and CMS, are to: (1) elucidate the mechanism of electroweak symmetry breaking and find the associated particle, which in the SM of particle physics is the Higgs boson^4–6^; and (2) search for BSM physics.

The necessity to study the wide range of processes in Fig. 1 largely drove the design of the ATLAS and CMS experiments. The production cross-sections and the decay branching fractions for a SM Higgs boson with a mass of 125.38 GeV are shown in Extended Data Table 1.

The LHC^20^ is designed to accelerate protons to an energy up to 7 TeV by powerful electric fields generated in superconducting radio-frequency cavities and guided around their circular orbits by strong (8.3 T) superconducting dipole magnets in tubes under very high vacuum. The counterrotating LHC beams are organized in approximately 2,800 bunches comprising more than 10^11^ protons per bunch, separated by 25 ns, leading to a bunch crossing rate of about 32 MHz. The two proton beams are brought into collision at the centre of the four LHC experiments. In Run 2, pp, interaction rates of 2 GHz were reached. Multiple pairs of protons interact in each bunch crossing, the average number ranging from 21 in 2012 to 32 in 2018. These are superposed on the triggered interaction and are labelled ‘pileup’.

### The CMS experiment

#### Design criteria and the SM Higgs boson

In the early 1990s, during the design phase of the Compact Muon Solenoid (CMS) experiment, considerable emphasis was placed on the identification and measurement of high-energy electrons, photons and muons, as these particles were expected to play an important role in the search for the SM Higgs boson and in the search for BSM physics.

As the rate of production of energetic muons at high-luminosity hadron colliders is very large, the online selection of events using muons is a particularly formidable task. The muon momentum has to be measured in real time and a momentum threshold placed to limit the rate. This requires a high bending power (high magnetic field) and an adequately precise and robust measurement of the trajectory of muons. This consideration determined the starting point of the design of CMS, and by implication the choice, size and the power of the analysing magnet. The next design priority was driven by the search for the Higgs boson via its decay H → γγ, requiring an excellent electromagnetic calorimeter (ECAL). The muon system and the ECAL were to be complemented by a precision inner-tracking system, immersed in a high magnetic field, giving good momentum resolution, and a hadron calorimeter (HCAL) that provided an almost full calorimetric coverage (for example, for the search for the Higgs boson if its mass turned out to be larger than 500 GeV).

#### The CMS detector

The longitudinal cut-away view of the CMS detector is shown in Extended Data Fig. 1. The CMS detector comprises four principal layers: the inner tracker, the ECAL, the HCAL and the muon system. The various types of detecting element and their channel counts are also indicated. Physics objects (for example, electrons, photons, muons, quark or gluon jets, and so on) are identified by different combinations of the patterns of energy deposits and/or traces in these four layers.

The defining choice and the central element of the CMS detector is the long (13 m), large-inner-diameter (about 6 m), state-of-the-art high-field (3.8 T) superconducting solenoid, generating the magnetic field for both the inner tracker and the muon system. The large size of the solenoid allows the inner tracker and almost all the calorimetry to be installed inside the solenoid.

##### Inner tracking

Particles emerge from the interaction region into the inner tracker, housed in a cylindrical volume with a length of 5.8 m and a diameter of 2.5 m. The particles first encounter the pixel detector, configured in three (four) cylindrical layers of silicon sensors in the barrel region, and two (three) disks in the endcap region before (after) 2017. The pixel detector is surrounded by 10 concentric layers of silicon sensors in the barrel region, with 10-cm-long or 20-cm-long silicon microstrips, and 12 vertical planes in each endcap region. Points are measured with an accuracy of about 15 μm in the bending plane. The geometric coverage extends down to angles of 9° from the beamline.

##### Electromagnetic and hadron calorimeters

The ECAL employs dense lead tungstate scintillating crystals. Each crystal has a length of about 23 cm that is sufficient to contain the full energy of high-energy electron and photon showers. The amount of generated or collected light is proportional to the energy of the incident particle. The fine transverse size of the crystals means that the energy of an electromagnetic shower is distributed over a cluster of crystals ranging from 9 (3 × 3) to 25 (5 × 5) crystals. The geometric coverage of the ECAL goes down to about 6° from the beamline.

The HCAL, comprising about 7,000 channels, is a sandwich of about 5-cm-thick brass absorber plates and about 4-mm-thick scintillator plates. The charged particles in the shower, generated in the absorber plates, traverse the scintillator plates and produce light that is collected and guided by fibres to the photodetectors. The geometric coverage of the HCAL goes down to about 6° from the beamline. This coverage is augmented by the very forward calorimeter, comprising an iron absorber with quartz fibres embedded in a matrix arrangement. The relativistic charged particles in the showers traverse the fibres and generate Cherenkov light, a part of which is guided by the fibres to the photodetectors. This calorimeter extends the calorimetric coverage down to an angle of about 0.75° from the beamline. The thickness of the hadron calorimetry is sufficient to absorb almost all of the energy of high-energy hadrons.

##### Muon system

Muons (and neutrinos) are the only particles that normally reach the muon system. All other particles deposit almost all of their energies in the calorimeters, and hence are said to have been absorbed. In addition to the measurements inside the inner tracker, the momentum of muons is measured a second time in gas-ionization chambers. These chambers are organized in four ‘stations’ that measure several points, to a precision of about 150 μm, and generate track segments whose direction is measured online with an angular precision of about 5 mrad. An independent set of gas-ionization chambers provide a signal timing resolution of about 3 ns, aiding the triggering process. The instrumented geometric coverage of the muon system goes down to an angle of 10° from the beamline.

##### Event selection

As the resources needed to record data for later use from all of the approximately 32 million beam crossings per second would be prohibitively costly, specific filters (known as triggers) are used to select the most interesting ones. An online two-tiered trigger system^26,27^ is deployed, with the first tier (Level 1) being hardware-based and the second one (high-level or HLT) being software-based. The Level 1 uses custom hardware that processes coarse information from the calorimeters or the muon chambers to select around 100,000 crossings of interest per second, corresponding to a reduction of a factor of about 400. Crossings of interest are selected if the energy deposits in the calorimeters or the momentum of muons, are above predefined thresholds. Upon the issuance of a Level-1 trigger, and after a fixed latency of just under 4μs, all data from the ‘triggered’ crossing are off-loaded from the pipeline memories in the approximately 100 million on-detector electronics channels. These data, after suitable treatment in electronics housed in the underground ‘services’ cavern, are sent up 100 m to the surface as fragments on approximately 1,000 optical fibres and fed into a commercial telecommunication ‘switch’. The switch takes the individual fragments, puts them together, ‘builds’ the event, and feeds the event into the next available central processing unit (CPU) core, in a computer farm of some 50,000 CPU cores. There, in real time, full-event physics-grade software algorithms, optimized for fast processing, reconstruct physics objects and select for permanent storage some 1,000 events or crossings per second, based on topological and kinematic information (Extended Data Table 3).

##### Event reconstruction

The CMS experiment generates a large amount of collision and simulated data. To handle, store and analyse all these data required the development of the worldwide LHC distributed computing grid (wLCG), providing universal access to data for all CMS Collaboration members.

The data from the stored events are transferred to the Tier-0 centre housed on CERN’s main site, where a first processing stage is performed. The result of this stage is then distributed to seven other major centres worldwide, labelled Tier-1 centres, for offline analysis. The Tier-1s are designed to carry out tasks of further reconstruction of the collision data with improved calibration and alignment of the various CMS subdetectors, whereas the generation and reconstruction of Monte Carlo event samples is carried out both at the Tier-1 centres and smaller university-based locations, labelled Tier-2 centres.

The particle-flow (PF) algorithm^31^ reconstructs and identifies each individual particle in an event, with an optimized combination of information from the various elements of the CMS detector. The energy of photons is obtained from the measurements in the ECAL. The energy of electrons is determined from a combination of the electron momentum at the primary interaction vertex as determined by the tracker, and the energy in the corresponding cluster of crystals, including the energy sum of all bremsstrahlung photons spatially compatible with originating from the electron track. The momentum of muons is derived from the curvature of the corresponding track. The energy of charged hadrons is determined from a combination of their momentum measured in the tracker, and the matching ECAL and HCAL energy deposits. The energy of neutral hadrons is obtained from the corresponding corrected ECAL and HCAL energies.

Hadronic jets, arising from quarks or gluons, are created from all the particles reconstructed by the PF algorithm within a cone of half-angle of about 25°, centred on the axis determined by the vectorial sum of the momenta of all particles in the jet.

##### Improvements of the CMS detector

Several improvements have been introduced into the CMS experiment since the discovery of the Higgs boson in 2012. These include:

- The replacement, in late 2016, of the silicon pixel detector, with a new one comprising four concentric layers in the barrel region, at radii of 29 mm, 68 mm, 109 mm and 160 mm, and six endcap disks placed at ±34, ±41, and ±51 mm from the interaction point, along the beam line. The new configuration leads to an improvement in the reconstruction of the secondary vertices and in the quality of tagging of b quarks. The sensitivity of H → bb analysis is found to be improved by a factor of 2.
- The replacement of photodetectors in HCAL (hybrid photodiodes replaced by silicon photomultipliers) and implementation of more precise timing, allowing a reduction of accidental or instrumental backgrounds, for example, stray or out-of-time particles.
- The installation in 2013 and 2014 of chambers in the fourth endcap muon station that were left out for Run 1.
- The upgrade of the Level-1 trigger hardware before LHC Run 2 to improve the selection of physics events of interest. The trigger rate from background processes is reduced and the trigger efficiency improved for a wide variety of physics signals. In the muon system, new trigger processor boards deploy powerful commercial field-programmable gate arrays (FPGAs). A time-multiplexed architecture was introduced that enabled data from all the calorimetry in each crossing to be pushed into a single FPGA of the type used in the muon trigger system. The FPGAs allow sophisticated and innovative algorithms to be implemented and evolved as conditions change.
- In the data acquisition system, a new switch was installed and the CPU power of the computer farm increased. The whole fabric of the distributed computing systems was upgraded to allow more events to be stored (at least 1,000 events per second instead of the initially foreseen 100 events per second).

### Offline event analysis

The principal physics objects are required to have transverse momenta or energies above a set threshold. The thresholds are lowered for the second, or any further, objects. Typical values of these thresholds are listed in Extended Data Table 3.

Leptons and photons resulting from the decays of Higgs bosons are expected to be unaccompanied by other particles; they are said to be ‘isolated’. Isolation criteria are imposed by requiring no additional energetic particles within a cone of about 20° opening angle around the object’s direction. Particles, other than from decays of b and c quarks or τ leptons, are expected to emerge directly from the primary interaction vertex, defined as the vertex corresponding to the pp collision identified by the online selection.

Increased use of regression and classification algorithms implemented using machine-learning methods, such as deep neural networks (DNNs) and boosted decision trees, led to a simultaneous increase in purity and in efficiencies of identification and reconstruction of physics objects (electrons, muons, photons, b quarks, τ leptons, jets and ${p}_{{\rm{T}}}^{{\rm{miss}}}$), and improvements in the calibration of related kinematic observables.

All analyses make extensive use of Monte Carlo simulation of the signal and background processes. The CMS detector is precisely described in software code that is used to generate Monte Carlo event samples. Multiple interactions are included, which match the distribution of the number of pileup interactions observed in data. All the simulated event samples are then processed through the same chain of software programs and procedures as are collision data. Simulated samples are used to evaluate or determine geometric acceptances, energy, momentum and mass resolutions, as well as for online and offline particle identification and reconstruction efficiencies, and for training for the many boosted decision tree algorithms and DNNs.

### Notes on Higgs boson decay channels

The distributions of the invariant mass of final-state particles in the individual decay channels are shown in Extended Data Figs. 3 and  4.

#### Bosonic decay channels

For H → γγ, the signal is extracted by measuring the narrow signal peak over a smoothly falling background distribution^42^. Despite its small branching fraction (0.23%), this mode is a sensitive one owing to the excellent precision in the measurement of the energies of photons. The diphoton invariant mass resolution is ${\sigma }_{{m}_{{\rm{\gamma }}{\rm{\gamma }}}}/{m}_{{\rm{H}}}\approx 1 \%$. All the principal production modes can be studied (ggH, VBF, VH, ttH and tH). The background largely consists of an irreducible one from quantum chromodynamics (QCD) production of two photons. There is also a reducible background where one or more of the reconstructed photon candidates originate from misidentification of jet fragments, that is dominated by QCD Compton scattering from quarks.

The study of the H → ZZ → 4${\ell }$ decay channel uses the distinctive decay of the Z bosons to charged leptons (${\ell }$) leading to a final state with 4e, or 4μ, or 2e2μ (ref. ^43^). The signal appears as a narrow peak on top of a smooth and small background. The momentum (energy) measurement of muons (electrons) is precise enough to give an invariant mass resolution with ${\sigma }_{{m}_{4{\ell }}}/{m}_{{\rm{H}}}\approx 1 \%$. The background comprises an irreducible part arising from the non-resonant production of two Z bosons or Zγ*, and a reducible part from the production of Z+ jets and top pair events, where the jets originate from heavy quarks, and thus could contain charged leptons, or are misidentified as charged leptons. The event yield for this process is tiny owing to the small branching fractions of H → ZZ (2.71%) and subsequent Z → ${\ell }{\ell }$ (3.37% per lepton type) decays. To enhance the signal over background and to categorize events, discriminants exploiting the production and decay kinematics expected for the signal and background events based on a matrix element likelihood approach are used together with the invariant mass of the particle.

**Extended Data Fig. 2:**
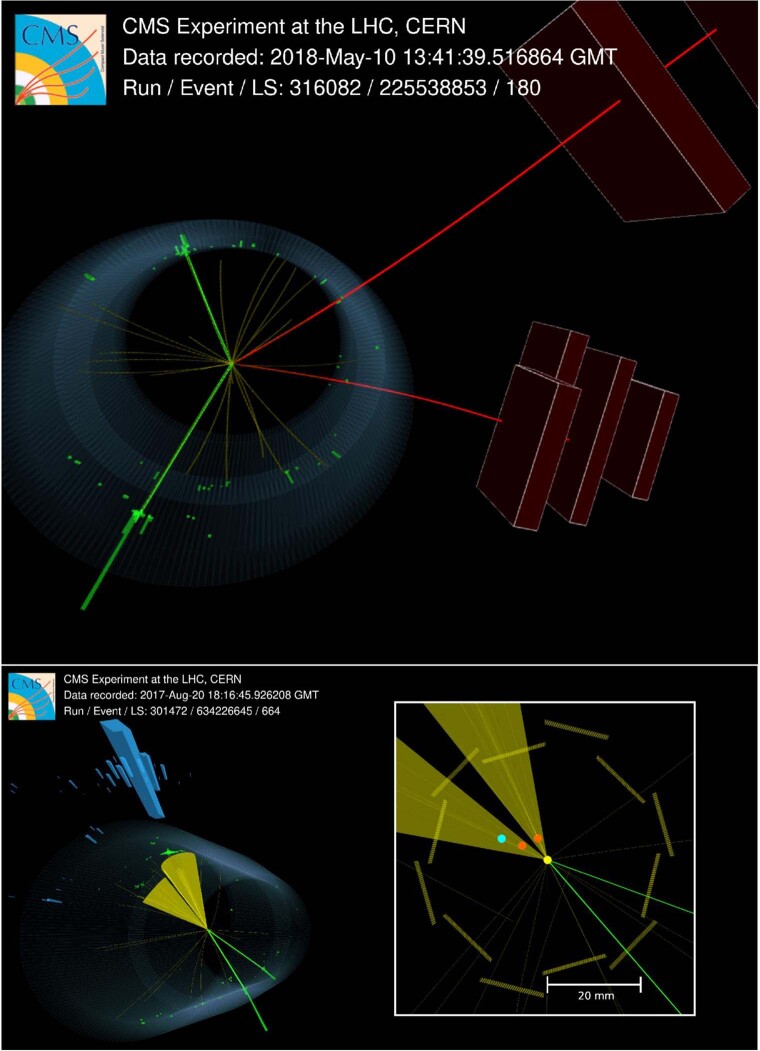
Higgs boson candidate events. (upper) An event display of a candidate H → ZZ → eeμμ. (lower) An event display of an H → bb candidate produced in association with a boson decaying into an electron–positron pair, in pp collisions at $\sqrt{s}=13\,{\rm{TeV}}$ recorded by CMS. The charged-particle tracks, as reconstructed in the inner tracker, are shown in yellow; the electrons are shown in green, the energy deposited by the electrons in the ECAL is shown as large green towers, the size of which is proportional of the amount of energy deposited; the blue towers are indicative of the energy deposits in the HCAL, while the red boxes are the muon chambers crossed by the muons (red tracks); the yellow cones represent the reconstructed jets. (lower, inset) The zoom into the collision region shows the displaced secondary vertices (in red) of the two b quarks decaying away from the primary vertex (in yellow). One of the bottom hadrons decays into a charm hadron that moves away from the secondary vertex before decaying (b → c → X; vertex in cyan).

**Extended Data Fig. 3:**
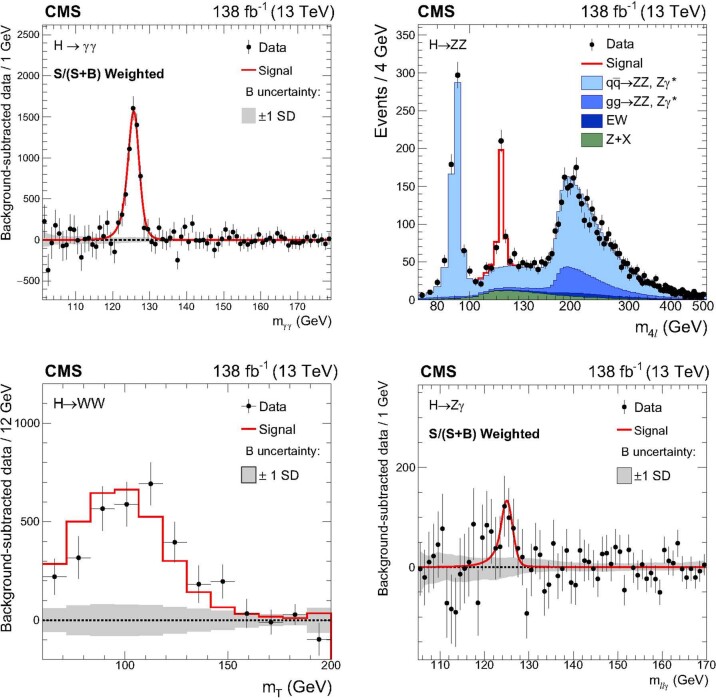
Higgs boson mass peak in diboson decay channels. (upper left) The background-subtracted diphoton invariant mass distribution targeting the study of the decay channel H → γγ. (upper right) The invariant mass distribution of four charged leptons targeting the study of the decay channel H → ZZ → 4*l*. (lower left) The background-subtracted transverse mass *m*_T_ distribution targeting the study of the decay channel H → WW. (lower right) The background-subtracted ${\ell }{\ell }\gamma$ invariant mass distribution targeting the study of the decay channel H → Zγ. The SM prediction for the signal (red line) is scaled by the value of *μ*, as estimated in the dedicated analysis for that channel, and computed for *m*_H_ = 125.38 GeV. The grey band around zero shows the 1 s.d. uncertainty in the background subtraction.

**Extended Data Fig. 4:**
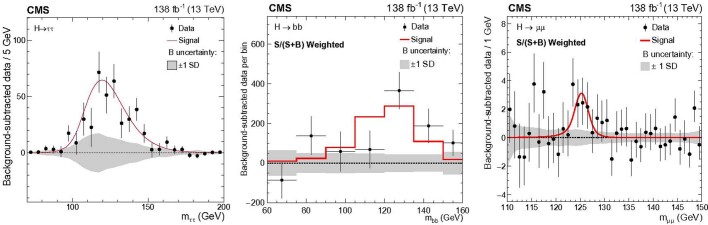
Higgs boson mass peak in difermion decay channels. The background-subtracted diparticle invariant mass distribution targeting the study of the decay channel (left) H → ττ, (center) H → bb, (right) H → μμ. The SM prediction for the signal (red line) is scaled by the value of *μ*, as estimated in the dedicated analysis for that channel, and computed for *m*_H_ = 125.38 GeV. The grey band around zero shows the 1 s.d. uncertainty in the background subtraction.

Extended Data Fig. 2 (top) shows a display of a candidate H → ZZ → eeμμ event produced in pp collisions at a centre-of-mass energy $\sqrt{s}=13\,{\rm{TeV}}$ and recorded in the CMS detector.

For H → WW → ${\ell }$ν${\ell }$ν, two high-*p*_T_${\ell }$ and large ${p}_{{\rm{T}}}^{{\rm{miss}}}$ characterize this final state^44^ and benefit from the H → WW decay having one of the largest branching fractions (about 22%). Owing to the presence of two neutrinos, the computation of the WW invariant mass is not possible. However, an associated variable, the transverse mass, *m*_T_, can be computed from the ${{\bf{p}}}_{{\rm{T}}}$ of the charged leptons and the ${{\bf{p}}}_{{\rm{T}}}^{{\rm{miss}}}$. The square of transverse mass for a collection of particles $[{P}_{i}]$ is defined as ${m}_{{\rm{T}}}^{2}([{P}_{i}])={(\sum | {{\bf{p}}}_{{\rm{T}},i}| )}^{2}-{| \sum {{\bf{p}}}_{{\rm{T}},i}| }^{2}$. The dominant background arises from irreducible non-resonant WW production and is estimated from data. The channel has a good sensitivity to the ggH and VBF production processes. In the analysis, $3{\ell }$ and $4{\ell }$ categories are also included, which are sensitive to production of the Higgs boson in association with a leptonically decaying vector boson. The analysis does not target the ttH and tH production modes, which are covered by a dedicated analysis discussed in ‘ttH and tH with multileptons’.

The H → Zγ signal is sought as a peak over a smoothly falling background distribution^45^. This analysis targets decays of the Z boson into 2e or 2μ. To increase the sensitivity to the signal, the events are divided into different categories on the basis of the production mode. Multivariate analysis (MVA) techniques are used to further categorize regions with high and low signal-to-background ratios. The dominant background arises from Drell–Yan dilepton production in association with an initial-state photon.

#### Fermionic decay channels

For H → ττ, four different ditau final states are studied^46^: eμ, eτ_h_, μτ_h_ and τ_h_τ_h_, where τ_h_ refers to a hadronically decaying τ lepton. The analysis of this decay channel targets the ggH, VBF and VH production modes. The identification of τ_h_ candidates uses DNN discriminants to reject quark and gluon jets misidentified as τ_h_. To separate the H → ττ signal events from the sizeable contribution of irreducible Z → ττ events, the likelihood estimate of the reconstructed mass of the system is used. This analysis does not target ttH production, which is covered by the dedicated analysis discussed in ‘ttH and tH with multileptons’.

The H → bb decay channel has by far the largest branching fraction of all the decay channels considered, with around 60% of Higgs bosons decaying in this way. The background from QCD production of pairs of b jets is very large; hence, final states with special characteristics have been chosen to enhance the signal-to-background ratio^47–51^.

To select jets most likely to originate from b quarks, a DNN algorithm is used^75,76^. It provides a continuous discriminant score, which combines information typical of b-quark jets, such as the presence of tracks displaced from the primary vertex, identified secondary vertices and the presence of low  *p*_T_ leptons in the jet. The threshold on the discriminant score is set such that the misidentification rate for light (u, d and s) quarks or gluons is low. For example, setting this misidentification rate at 0.1% gives a 50% efficiency for b-quark jet identification when applied to jets in top quark–antiquark events.

The VH production mode uses the presence of one or more leptons from the decay of the vector boson, or large ${p}_{{\rm{T}}}^{{\rm{miss}}}$. In the signal-sensitive region, DNNs are used to separate the signal from the background dominated by QCD multijet production.

The ttH and tH production modes are included in the combination and MVA techniques are used to separate the signal from the large multijet backgrounds. This analysis uses the 2016 dataset.

Lastly, an inclusive analysis is included that targets Higgs bosons produced with large *p*_T_ (ref. ^51^). In this kinematic region, the signal-to-background ratio is larger. The two b jets from decays of highly Lorentz-boosted Higgs bosons are close in space and appear in the detector as a single broad jet with distinctive internal structure.

Extended Data Fig. 2 (bottom) shows a candidate H → bb event produced in pp collisions at a centre-of-mass energy $\sqrt{s}=13\,{\rm{TeV}}$ and recorded in the CMS detector.

The H → μμ signal is searched for as a peak in the dimuon mass distribution, over a smoothly falling background^52^. The dimuon invariant mass resolution is ${\sigma }_{{m}_{{\rm{\mu }}{\rm{\mu }}}}/{m}_{{\rm{H}}}\approx 1 \%$. The analysis of this decay channel targets the ggH, VBF, VH and ttH production modes, and is most sensitive in the first two modes. The largest background in this decay channel comes from Drell–Yan dimuon production in which an off-shell Z* boson decays to a pair of muons. Events are split into production modes based on their kinematical properties. To improve the sensitivity of the analysis, MVA techniques are used in each of these different categories.

The analysis of the H → cc final state in the VH production mode (Fig. 1c) has recently been presented^74^ but has not been included in the present combination. The analysis yields $\sigma ({\rm{V}}{\rm{H}}){\mathcal{B}}({\rm{H}}\to {\rm{c}}{\rm{c}}) < 0.94\,$ pb at the 95% CL. The observed 95% CL interval (expected upper limit) for *κ* is found to be $1.1 < |{\kappa }_{{\rm{c}}}| < 5.5$ ($| {\kappa }_{{\rm{c}}}|  < 3.4$), the most stringent so far. A search for Z → cc in VZ events is used to validate the analysis strategy and yields a first observation of this decay channel, at a hadron collider, with a significance of 5.7 s.d.

#### ttH and tH with multileptons

The ttH (Fig. 1d) and tH (Fig. 1e,f) production channels, which probe the coupling of the Higgs boson to the top quarks, are studied in the case where the Higgs boson and the top quarks subsequently decay into final states with several leptons^53^, supplementing dedicated studies of the H → γγ, H → ZZ → 4${\ell }$ and H → bb decay modes.

This analysis uses a categorization based on the number of leptons and/or τ_h_ candidates to target both the different Higgs boson final states and the tt decay channels. Categories with at least two leptons, or one lepton and two τ_h_ candidates, target cases where at least one top quark decays via a leptonically decaying W boson. Categories with one lepton and one τ_h_, or with no leptons and two τ_h_ candidates are used to target events in which both top quarks decay via hadronically decaying W bosons. This analysis is sensitive to the H → WW, H → ττ and H → ZZ decay channels. Several MVA techniques are employed to better separate the ttH and tH production modes.

#### Higgs boson decays beyond the SM

In addition to the invisible Higgs boson decays discussed in ‘The κ framework for coupling modifiers’, other BSM decays are possible, into undetected particles. That is, these particles may or may not leave a trace in the CMS detector, but we do not have dedicated searches looking for these signatures. Nevertheless, the presence of undetected decays can be inferred indirectly from a reduction in the branching fraction for SM decays (or by an increase in the total Higgs boson width). In this interpretation, the total width becomes ${\varGamma }_{{\rm{H}}}=\sum {\varGamma }_{f}(\kappa )/(1-{{\mathcal{B}}}_{{\rm{Inv}}.}-{{\mathcal{B}}}_{{\rm{Undet}}.})$, where ${ {\mathcal B} }_{{\rm{Undet}}.}$ is the branching fraction to undetected particles.

To probe invisible or undetected decays of the Higgs boson, another fit can be performed, including ${ {\mathcal B} }_{{\rm{Inv}}.}$ and ${ {\mathcal B} }_{{\rm{Undet}}.}$ as additional floating parameters, while imposing as an upper bound on *κ*_W_ and *κ*_Z_ their SM values, also valid in most proposed extensions of the SM^77,78^. As can be seen from Extended Data Fig. 8 (right), ${ {\mathcal B} }_{{\rm{Inv}}.}$ and ${ {\mathcal B} }_{{\rm{Undet}}.}$ are found to be consistent with zero. The 95% CL upper limit on ${ {\mathcal B} }_{{\rm{Undet}}.}$ is found to be <0.16, with only small changes to the other *κ*_*i*_ fitted values, as shown in Extended Data Fig. 8 (right). The measurement of the width^68^ of the Higgs boson will be used in the future to constrain these quantities without imposing bounds on *κ*_W_ and *κ*_Z_.

### Statistical analysis

The statistical framework used to build the combination of all the channels is based on an established combined likelihood method (ref. ^40^ and references therein), and briefly detailed in this section.

Given the enormous number of pp collisions produced at the LHC and the relatively small probability that one of those collisions will produce a signal-like event, the observations in data are described by Poisson probability functions, ${\mathscr{P}}(k|\lambda )={{\rm{e}}}^{-\lambda }{\lambda }^{k}/k!$, where *k* is the observed number of events, and the parameter *λ* is the expected number of events in a particular bin or region of one or more of the discriminating distributions used for each channel entering the combination. The combined likelihood is obtained from the product of the individual Poisson probability functions, accounting for the observed data and expected number of events across all channels.

The parameters *λ* are functions of the model parameters of interest: *μ*, which represent the Higgs boson couplings or signal strengths, and nuisance parameters *θ*, which model the effect of systematic uncertainties on the predicted signal and background contributions. Additional terms are included in the combined likelihood to represent constraints on the nuisance parameters owing to external measurements, such as energy- and momentum-scale calibrations or an integrated luminosity determination. The measurements reported in this paper are determined using the profile likelihood ratio $q(\mu )=-\,2\,\mathrm{ln}\, {\mathcal L} (\mu ,{\hat{\theta }}_{\mu })/ {\mathcal L} (\hat{\mu },\hat{\theta })$ where $\hat{\mu }$ and $\hat{\theta }$ are the values of the parameters of interest and nuisance parameters that maximize the likelihood ${\mathcal L} (\mu ,\theta )$, and ${\hat{\theta }}_{\mu }$ are the values of the nuisance parameters that maximize the likelihood for a fixed value of $\mu$. The compatibility between a given set of measurements and their corresponding SM predictions is reported as a *P* value, derived from the difference between *q*_SM_ and $q(\hat{\mu })$. Expected intervals are derived from the Asimov dataset, in which the nuisance parameters are set to their maximum likelihood estimator values.

The modified likelihood ratio test statistic $$\tilde{q}(\mu )=-\,2\,\mathrm{ln}\,[ {\mathcal L} (\mu ,{\hat{\theta }}_{\mu })/$$$$ {\mathcal L} (\hat{\mu },\hat{\theta })]$$ with a constraint $$0\le \hat{\mu }\le \mu $$ is used to set 95% CL upper limits on signal strengths and production cross-sections using the “CL_*s*_ criterion”^40^.

All the reported confidence intervals, confidence regions and *P* values are obtained assuming various asymptotic approximations for the distributions of the (modified) likelihood ratio test statistic^79^. The validity of the asymptotic assumptions has been routinely checked in the context of individual analyses whenever the event yields are small or particular validity conditions are not met.

### Signal strengths of production channels and decay modes

For a Higgs boson produced in mode *i* and decaying into a final state *f*, the signal event yields are proportional to ${\sigma }_{i}{ {\mathcal B} }^{f}$, where *σ*_*i*_ is the production cross-section and ${ {\mathcal B} }^{f}$ is the decay branching fraction. The branching fraction is in turn given by ${{\mathcal{B}}}^{f}={\varGamma }^{f}/{\varGamma }_{{\rm{H}}},$ where *Γ*^*f*^ is the partial decay width in the final state *f* and *Γ*_H_ the total natural width of the Higgs boson.

Fits are performed under different assumptions: per overall single signal strength, yielding *μ* = 1.002 ± 0.057; per production channel signal strengths (${\mu }_{i}={\sigma }_{i}/{\sigma }_{i}^{{\rm{SM}}}$ with ${ {\mathcal B} }^{f}={ {\mathcal B} }_{{\rm{SM}}}^{f}$), Fig. 2 (left); per decay mode signal strengths (${\mu }^{f}={ {\mathcal B} }^{f}/{ {\mathcal B} }_{{\rm{SM}}}^{f}$, with ${\sigma }_{i}={\sigma }_{i}^{{\rm{SM}}}$), Fig. 2 (right); and with a free parameter per individual combination of production modes and decay channels, as illustrated in Extended Data Fig. 6.

**Extended Data Fig. 5:**
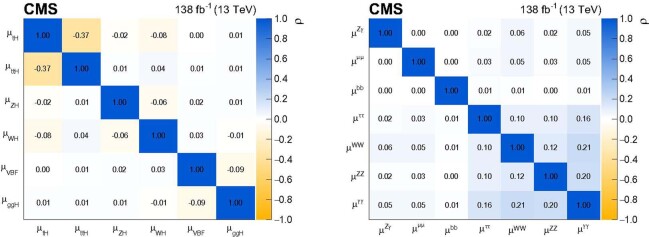
Correlations between the measurements of different couplings. Covariance matrices for the fits of the signal-strength parameters per production mode *μ*_*i*_ (left) and per decay mode *μ*^*f*^ (right). The values of the correlation coefficients, *ρ*, are indicated both in text and in the color scale.

**Extended Data Fig. 6:**
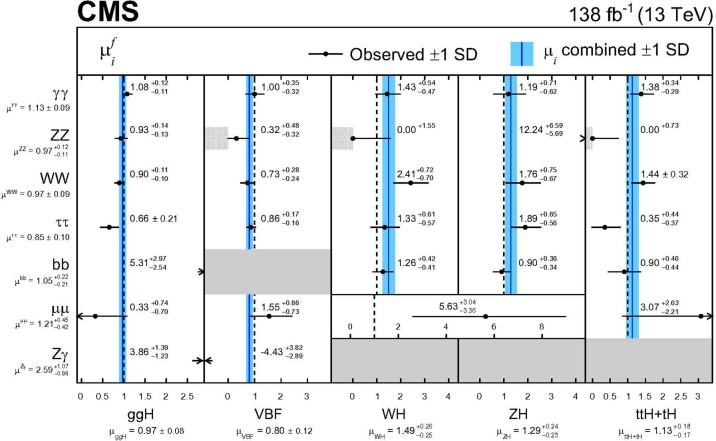
The agreement with the SM predictions in Higgs boson production and decay. Signal-strength parameters per individual production mode and decay channel ${\mu }_{i}^{f}$, and combined per production mode *μ*_*i*_ and decay channel *μ*^*f*^. In this fit, ttH and tH are considered together and the *μ*_*i*_ results are slightly different from those of Fig. 2 (left). The dashed vertical lines at 1 represent the SM value. Light grey shading indicates that *μ* is contained to be positive. Dark grey shading indicates the absence of measurement. The *p*-value with respect to the SM prediction is 5.8%.

**Extended Data Fig. 7:**
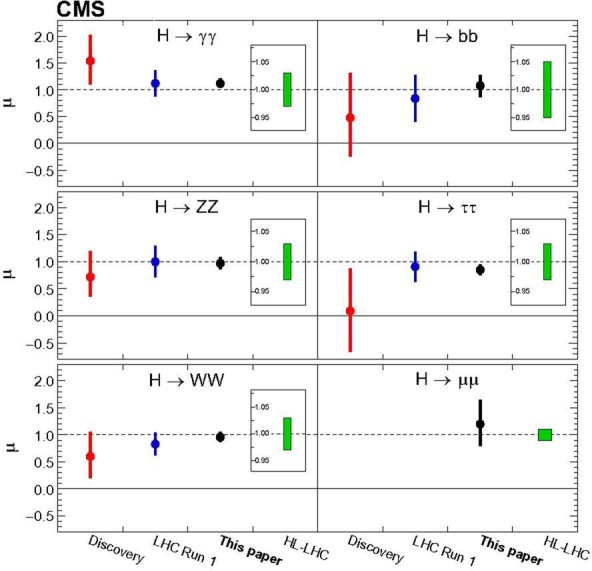
Time evolution of the signal-strength measurements and their precision. Comparison of the signal-strength parameter *μ* fit results in different datasets; in each panel, from left to right: at the time of the Higgs boson discovery, using the full data from LHC Run 1, in the dataset analyzed for this paper, and the expected 1 s.d. uncertainty for HL-LHC for ${\mathcal L} =3000\,{{\rm{fb}}}^{-1}$. The H → μμ measurements were not available for the earlier datasets due to the lack of sensitivity.

**Extended Data Fig. 8:**
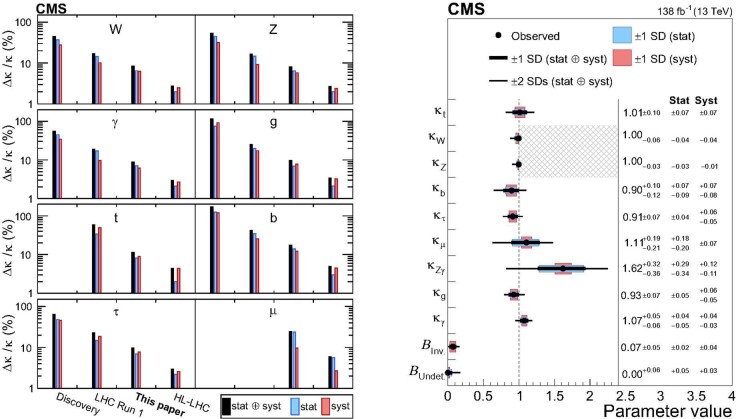
Time evolution of the coupling measurements and their precision. (left) Comparison of the expected 1 SD uncertainties in the *κ*-framework fit including coupling modifiers for both tree-level and loop-induced Higgs boson interactions, in different datasets: at the time of the Higgs boson discovery, using the full data from LHC Run 1, in the dataset used in this paper, and the expected 1SD uncertainty for HL-LHC for ${\mathcal L} =3000\,{{\rm{fb}}}^{-1}$. (right) Results of a fit to the coupling modifiers *κ* allowing both invisible and the undetected decay modes, with the SM value used as an upper bound on both *κ*_W_ and *κ*_Z_. The thick (thin) black lines indicate the 1 (2) s.d. confidence intervals, with the systematic and statistical components of the 1 s.d. interval indicated by the red and blue bands, respectively. The *p*-value with respect to the SM prediction is 33%.

The covariance matrices for the fitted signal strengths per production mode ${\mu }_{i}$ and per decay channel *μ*^*f*^ are shown in Extended Data Fig. 5.

### Notes on self-interaction strength

The potential energy of the BEH field (*ϕ*) is given by $$V(\phi )\,=$$$$\frac{1}{2}{m}_{{\rm{H}}}^{2}{\phi }^{2}+\sqrt{\lambda /2}{m}_{{\rm{H}}}{\phi }^{3}+\frac{1}{4}\lambda {\phi }^{4}.$$ The first term accounts for the mass of the Higgs boson *m*_H_. The second term represents the Higgs boson self-interaction, of strength *λ*. In the SM, $$\lambda ={m}_{{\rm{H}}}^{2}/(2{\upsilon }^{2})$$ (where the vacuum expectation value of the BEH field, corresponding to its minimum, is $$\upsilon =246\,{\rm{GeV}}$$) and it can be measured via the study of Higgs boson pair production. The third term represents the interaction of four Higgs bosons at a point, a process that is even rarer than its pair production. Knowledge of the exact shape of the potential *V* is crucial for understanding the phase transition that occurred in the early Universe and its consequences^80^.

The search for Higgs boson pair production is performed by combining Higgs boson pairs, each with differing decay modes. The decay modes that have been used are bb, ττ and WW^57–60^, benefitting from the large branching fractions, and γγ^61^ and ${\rm{ZZ}}\to 4{\ell }$^62^, benefitting from the presence of narrow mass peaks, thus improving the signal-to-background ratio. All final states analysed are defined to be mutually exclusive so that they could be properly combined as statistically independent observations.

Measurements of Higgs boson pair production are used to constrain the Higgs boson self-interaction strength *λ*. Several combinations of individual Higgs boson decay modes are used in this search. The highest rate for Higgs boson pair production and decays occurs when both Higgs bosons decay to b-quark pairs, HH → bbbb, corresponding to about 35% of all the possible HH decays in the SM.

The search in the 4b decay mode^57,58^ is performed separately under the assumptions that ${m}_{{{\rm{H}}}^{* }}\gg 2{m}_{{\rm{H}}}$ or not. In the case ${m}_{{{\rm{H}}}^{* }}\gg 2{m}_{{\rm{H}}}$, each Higgs boson is energetic (and hence said to be boosted), such that its decay products, for example, b-quark jets, merge and appear as one broad jet, but with a distinctive internal structure. In the latter case, all four b-quark jets rarely overlap, and hence are said to be resolved.

Another group of analyses targets the HH final states where one H decays to b quarks and the other to ττ^59^, γγ^61^ or ${\rm{ZZ}}\to 4{\ell }$^62^. Analyses targeting a set of multileptons final states with ${p}_{{\rm{T}}}^{{\rm{miss}}}$ are HH → (WW)(WW), HH → (WW)(ττ) or HH → (ττ)(ττ)^60^, where hadronic τ lepton decays are also included.

**Extended Data Fig. 9:**
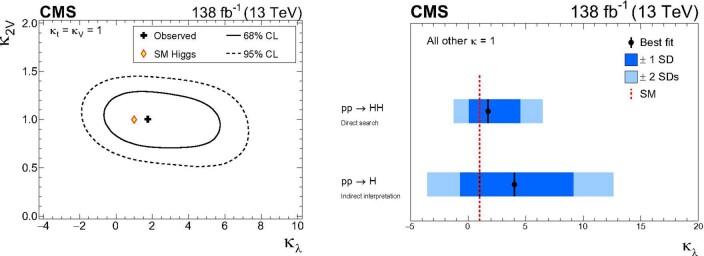
Constrains on Higgs boson self-interaction and quartic coupling. (left) Constraints on *κ*_λ_ and *κ*_2V_ from the production of Higgs boson pairs. (right) Constraint on the Higgs boson self-coupling modifier *κ*_λ_ from single and pair production of Higgs boson(s).

A fit to Higgs boson pair production data can be used to simultaneously constrain *κ*_λ_ and *κ*_2V_, as shown in Extended Data Fig. 9 (left).

Measurements of single Higgs boson production and decay can also be used to constrain *κ*_λ_ as quantum corrections to the SM Higgs boson production modes and decay channels depend on *κ*_λ_ (refs. ^81,82^). These corrections have been derived^83^ for the different production and decay modes entering the combination, as shown in Extended Data Table 2.

The values of *κ*_λ_ extracted from single and pair Higgs boson production are shown in Extended Data Fig. 9 (right).

### Upgrade of the CMS experiment for HL-LHC running

To exploit the full potential of the LHC, the accelerator and its experiments will be upgraded. The HL-LHC will operate at an instantaneous luminosity of 5 × 10^34^ cm^−2^ s^−1^. The intention is to collect ten times more data than the 300 fb^−1^ foreseen in the initial LHC phase. This means that the integrated radiation levels will be correspondingly larger.

The physics to be studied drives the technical choices for the upgrade. The physics goals are:

- precise measurements of the properties of the Higgs boson and its self-coupling, to elucidate further the physics of electroweak symmetry breaking;
- search for BSM physics; and
- selected precision SM measurements.

The translation of these physics goals into experimental design goals requires:

- The construction of a new higher-granularity, more radiation-hard silicon tracker. The design of the new front-end electronics will allow information from the inner tracker to participate in the Level-1 trigger. The size of the individual detecting elements will be decreased leading to about ten times larger number of electronics channels. All components inside the tracker (silicon sensors, front-end electronics, 10 Gb s^−1^ data links and so on) will have to withstand integrated doses of up to 500 Mrad and fluences of 10^16^ (1 MeV equivalent neutrons) per cm^2^. The geometric coverage of the inner tracker will be increased, extending it down an angle of 2° from the beamline.
- The replacement of other components affected by radiation. Principally, these are the endcap calorimeters and the ECAL front-end electronics. The endcap calorimeters will be replaced with a new high-granularity ‘imaging’ calorimeter with precision timing. It will be based on 600 m^2^ of silicon sensors with detecting cells of sizes of 0.5 cm^2^ to 1.0 cm^2^. Regions in this calorimeter will reach integrated doses of up to 500 Mrad and fluences of 10^16^ (1 MeV equivalent neutrons) per cm^2^. The new front-end electronics for the ECAL barrel will allow data from each crystal to be sent to the calorimeter Level-1 trigger processor, instead of the sum of 25 crystals today, and which will allow better measurement of the timing of the impact of electrons or photons.
- Higher-bandwidth Level-1 and high-level triggers. Information from the inner trackers will be used at Level 1. The Level-1 trigger latency will be increased from 4 μs to over 12 μs, requiring corresponding changes in the front-end electronics, allowing more processing time leading to a purer selection of events. The output rate from the Level-1 processors will be increased from 100 kHz to 750 kHz and correspondingly the number of events stored for later analysis will be increased from 1 kHz to 10 kHz.
- The introduction of precision timing detectors. A new set of detectors will be installed in the barrel and endcap regions, covering a region down to an angle of 9° from the beamline. The precision timing of photons (in the barrel region) and charged tracks will greatly improve the localization of the correct interaction vertex. At HL-LHC, on average, some 140 pairs of protons are expected to interact in each crossing, spread over a time characterized by *σ* ≈ 200 ps. Furthermore, suppression of energy can be carried out that is not consistent in time with the interaction of interest.

The upgraded CMS experiment at HL-LHC will be more powerful than the current one. Uncertainties in many measurements of the properties of the Higgs boson are expected to approach the percent level, benefitting from the anticipated larger event samples, reduced experimental systematic uncertainties and more accurate theoretical calculations.

### Theoretical references

The theoretical works used in our analyses can be found in the LHC Higgs Cross Section Working Group reports^36–39^ and in refs. ^54,56,84–108^.

## Online content

Any methods, additional references, Nature Research reporting summaries, source data, extended data, supplementary information, acknowledgements, peer review information; details of author contributions and competing interests; and statements of data and code availability are available at 10.1038/s41586-022-04892-x.

## Figures and Tables

**Extended Data Table 1:**
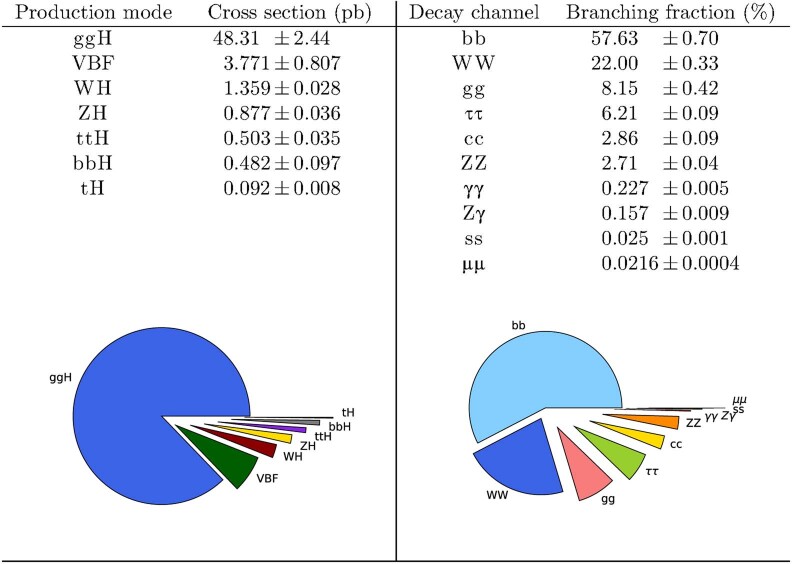
The SM Higgs production cross-sections and branching fractions

Theoretical cross-sections for each production mode and branching fractions for the decay channels, at $\sqrt{s}=13\,{\rm{TeV}}$ and for *m*_H_ = 125.38 GeV (ref. ^39^).

**Extended Data Table 2:**
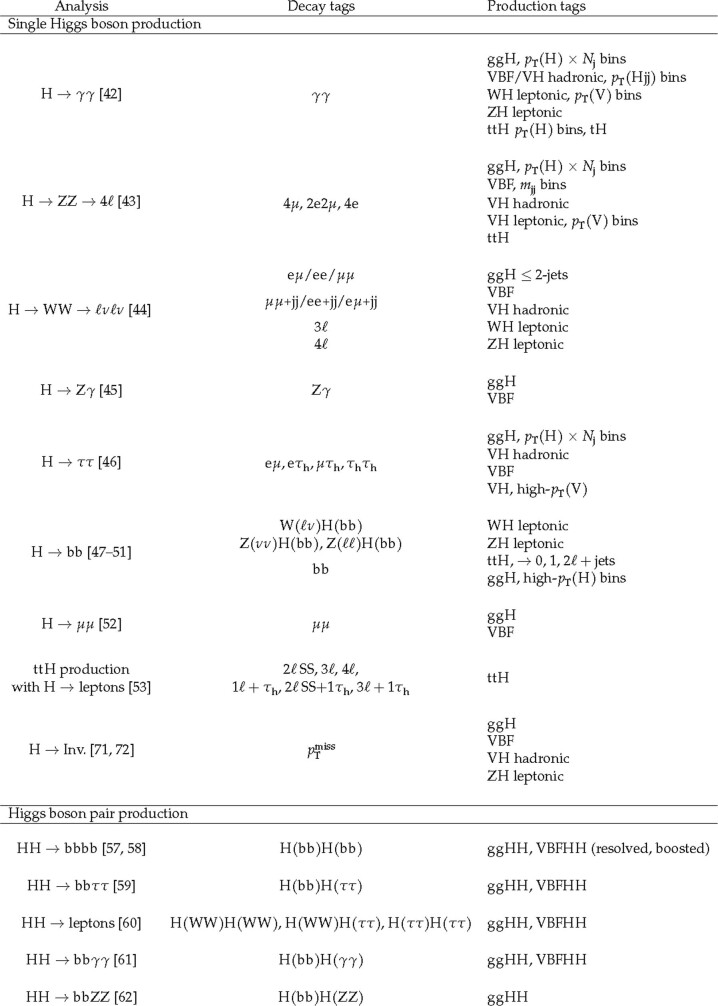
Summary of the analyses included in this paper

The analysis and decay channels are indicated in the first two columns, with the third column containing the production mechanism and kinematic regions targeted by each analysis. All analyses, apart from ttH in the H → bb final state (2016 data only) and VH in the H → bb final state (2016–2017 data), use the full dataset collected in Run 2. The various symbols are as follows: ${\ell }$ is e or μ, jet (j), di-jet mass (*m*_jj_), number of jets (*N*_j_), same-sign (SS) of electric charge, hadronic decay of the τ lepton (τ_h_).

**Extended Data Table 3:**
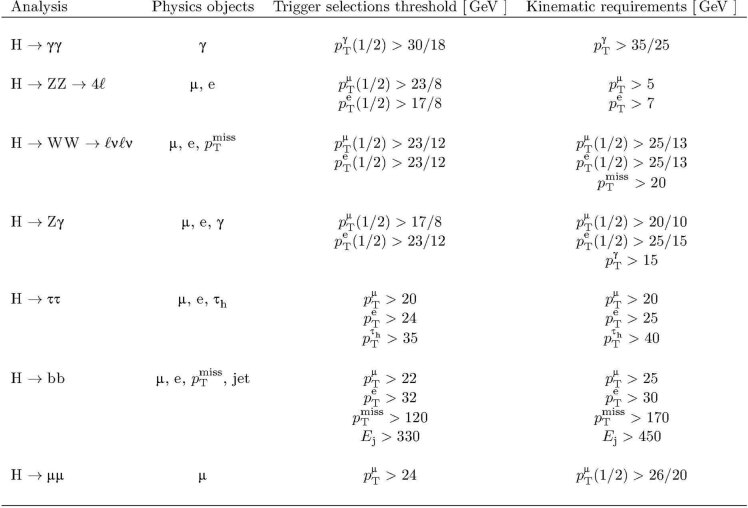
Summary of the event selection

Some of the typical selection criteria used in the trigger (online selection) and in offline analysis for some of the final states and for leading (1) and subleading (2) particles. The ${p}_{{\rm{T}}}^{{\rm{miss}}}$ is a measure of the imbalance in energy in the plane transverse to the colliding proton beams.

## Data Availability

Tabulated results are provided in the HEPData record for this analysis. Release and preservation of data used by the CMS Collaboration as the basis for publications is guided by the CMS data preservation, re-use and open acess policy.

## Code availability

The CMS core software is publicly available on GitHub (https://github.com/cms-sw/cmssw).
