# Author Correction: A portrait of the Higgs boson by the CMS experiment ten years after the discovery

**DOI:** 10.1038/s41586-023-06164-8

**Published:** 2023-10-18

**Authors:** The CMS Collaboration, A. Tumasyan, W. Adam, J. W. Andrejkovic, T. Bergauer, S. Chatterjee, K. Damanakis, M. Dragicevic, A. Escalante Del Valle, P. S. Hussain, M. Jeitler, N. Krammer, L. Lechner, D. Liko, I. Mikulec, P. Paulitsch, F. M. Pitters, J. Schieck, R. Schöfbeck, D. Schwarz, S. Templ, W. Waltenberger, C.-E. Wulz, M. R. Darwish, T. Janssen, T. Kello, H. Rejeb Sfar, P. Van Mechelen, E. S. Bols, J. D’Hondt, A. De Moor, M. Delcourt, H. El Faham, S. Lowette, S. Moortgat, A. Morton, D. Müller, A. R. Sahasransu, S. Tavernier, W. Van Doninck, D. Vannerom, B. Clerbaux, G. De Lentdecker, L. Favart, J. Jaramillo, K. Lee, M. Mahdavikhorrami, I. Makarenko, A. Malara, S. Paredes, L. Pétré, N. Postiau, E. Starling, L. Thomas, M. Vanden Bemden, C. Vander Velde, P. Vanlaer, D. Dobur, J. Knolle, L. Lambrecht, G. Mestdach, M. Niedziela, C. Rendón, C. Roskas, A. Samalan, K. Skovpen, M. Tytgat, N. Van Den Bossche, B. Vermassen, L. Wezenbeek, A. Benecke, G. Bruno, F. Bury, C. Caputo, P. David, C. Delaere, I. S. Donertas, A. Giammanco, K. Jaffel, Sa. Jain, V. Lemaitre, K. Mondal, J. Prisciandaro, A. Taliercio, T. T. Tran, P. Vischia, S. Wertz, G. A. Alves, E. Coelho, C. Hensel, A. Moraes, P. Rebello Teles, W. L. Aldá Júnior, M. Alves Gallo Pereira, M. Barroso Ferreira Filho, H. Brandao Malbouisson, W. Carvalho, J. Chinellato, E. M. Da Costa, G. G. Da Silveira, D. De Jesus Damiao, V. Dos Santos Sousa, S. Fonseca De Souza, J. Martins, C. Mora Herrera, K. Mota Amarilo, L. Mundim, H. Nogima, A. Santoro, S. M. Silva Do Amaral, A. Sznajder, M. Thiel, F. Torres Da Silva De Araujo, A. Vilela Pereira, C. A. Bernardes, L. Calligaris, E. M. Gregores, P. G. Mercadante, S. F. Novaes, Sandra S. Padula, T. R. Fernandez Perez Tomei, A. Aleksandrov, G. Antchev, R. Hadjiiska, P. Iaydjiev, M. Misheva, M. Rodozov, M. Shopova, G. Sultanov, A. Dimitrov, T. Ivanov, L. Litov, B. Pavlov, P. Petkov, A. Petrov, E. Shumka, T. Cheng, T. Javaid, M. Mittal, L. Yuan, M. Ahmad, G. Bauer, Z. Hu, S. Lezki, K. Yi, G. M. Chen, H. S. Chen, M. Chen, F. Iemmi, C. H. Jiang, A. Kapoor, H. Liao, Z.-A. Liu, V. Milosevic, F. Monti, R. Sharma, J. Tao, J. Thomas-Wilsker, J. Wang, H. Zhang, J. Zhao, A. Agapitos, Y. An, Y. Ban, C. Chen, A. Levin, C. Li, Q. Li, X. Lyu, Y. Mao, S. J. Qian, X. Sun, D. Wang, J. Xiao, H. Yang, J. Li, M. Lu, Z. You, X. Gao, D. Leggat, H. Okawa, Y. Zhang, Z. Lin, C. Lu, M. Xiao, C. Avila, D. A. Barbosa Trujillo, A. Cabrera, C. Florez, J. Fraga, J. Mejia Guisao, F. Ramirez, M. Rodriguez, J. D. Ruiz Alvarez, D. Giljanovic, N. Godinovic, D. Lelas, I. Puljak, Z. Antunovic, M. Kovac, T. Sculac, V. Brigljevic, B. K. Chitroda, D. Ferencek, D. Majumder, M. Roguljic, A. Starodumov, T. Susa, A. Attikis, K. Christoforou, G. Kole, M. Kolosova, S. Konstantinou, J. Mousa, C. Nicolaou, F. Ptochos, P. A. Razis, H. Rykaczewski, H. Saka, M. Finger, M. Finger, A. Kveton, E. Ayala, E. Carrera Jarrin, A. A. Abdelalim, E. Salama, M. A. Mahmoud, Y. Mohammed, S. Bhowmik, R. K. Dewanjee, K. Ehataht, M. Kadastik, S. Nandan, C. Nielsen, J. Pata, M. Raidal, L. Tani, C. Veelken, P. Eerola, H. Kirschenmann, K. Osterberg, M. Voutilainen, S. Bharthuar, E. Brücken, F. Garcia, J. Havukainen, M. S. Kim, R. Kinnunen, T. Lampén, K. Lassila-Perini, S. Lehti, T. Lindén, M. Lotti, L. Martikainen, M. Myllymäki, J. Ott, M. M. Rantanen, H. Siikonen, E. Tuominen, J. Tuominiemi, P. Luukka, H. Petrow, T. Tuuva, C. Amendola, M. Besancon, F. Couderc, M. Dejardin, D. Denegri, J. L. Faure, F. Ferri, S. Ganjour, P. Gras, G. Hamel de Monchenault, P. Jarry, V. Lohezic, J. Malcles, J. Rander, A. Rosowsky, M. Ö. Sahin, A. Savoy-Navarro, P. Simkina, M. Titov, C. Baldenegro Barrera, F. Beaudette, A. Buchot Perraguin, P. Busson, A. Cappati, C. Charlot, O. Davignon, B. Diab, G. Falmagne, B. A. Fontana Santos Alves, S. Ghosh, R. Granier de Cassagnac, A. Hakimi, B. Harikrishnan, J. Motta, M. Nguyen, C. Ochando, L. Portales, J. Rembser, R. Salerno, U. Sarkar, J. B. Sauvan, Y. Sirois, A. Tarabini, E. Vernazza, A. Zabi, A. Zghiche, J.-L. Agram, J. Andrea, D. Apparu, D. Bloch, G. Bourgatte, J.-M. Brom, E. C. Chabert, C. Collard, D. Darej, U. Goerlach, C. Grimault, A.-C. Le Bihan, P. Van Hove, S. Beauceron, C. Bernet, G. Boudoul, C. Camen, A. Carle, N. Chanon, J. Choi, D. Contardo, P. Depasse, C. Dozen, H. El Mamouni, J. Fay, S. Gascon, M. Gouzevitch, G. Grenier, B. Ille, I. B. Laktineh, M. Lethuillier, L. Mirabito, S. Perries, K. Shchablo, V. Sordini, L. Torterotot, M. Vander Donckt, P. Verdier, S. Viret, I. Lomidze, T. Toriashvili, Z. Tsamalaidze, V. Botta, L. Feld, K. Klein, M. Lipinski, D. Meuser, A. Pauls, N. Röwert, M. Teroerde, S. Diekmann, A. Dodonova, N. Eich, D. Eliseev, M. Erdmann, P. Fackeldey, B. Fischer, T. Hebbeker, K. Hoepfner, F. Ivone, M. Y. Lee, L. Mastrolorenzo, M. Merschmeyer, A. Meyer, S. Mondal, S. Mukherjee, D. Noll, A. Novak, F. Nowotny, A. Pozdnyakov, Y. Rath, W. Redjeb, H. Reithler, A. Schmidt, S. C. Schuler, A. Sharma, L. Vigilante, S. Wiedenbeck, S. Zaleski, C. Dziwok, G. Flügge, W. Haj Ahmad, O. Hlushchenko, T. Kress, A. Nowack, O. Pooth, A. Stahl, T. Ziemons, A. Zotz, H. Aarup Petersen, M. Aldaya Martin, P. Asmuss, S. Baxter, M. Bayatmakou, O. Behnke, A. Bermúdez Martínez, S. Bhattacharya, A. A. Bin Anuar, F. Blekman, K. Borras, D. Brunner, A. Campbell, A. Cardini, C. Cheng, F. Colombina, S. Consuegra Rodríguez, G. Correia Silva, M. De Silva, L. Didukh, G. Eckerlin, D. Eckstein, L. I. Estevez Banos, O. Filatov, E. Gallo, A. Geiser, A. Giraldi, G. Greau, A. Grohsjean, V. Guglielmi, M. Guthoff, A. Jafari, N. Z. Jomhari, B. Kaech, M. Kasemann, H. Kaveh, C. Kleinwort, R. Kogler, M. Komm, D. Krücker, W. Lange, D. Leyva Pernia, K. Lipka, W. Lohmann, R. Mankel, I.-A. Melzer-Pellmann, M. Mendizabal Morentin, J. Metwally, A. B. Meyer, G. Milella, A. Kasem, M. Mormile, A. Mussgiller, A. Nürnberg, Y. Otarid, D. Pérez Adán, A. Raspereza, B. Ribeiro Lopes, J. Rübenach, A. Saggio, A. Saibel, M. Savitskyi, M. Scham, V. Scheurer, S. Schnake, P. Schütze, C. Schwanenberger, M. Shchedrolosiev, R. E. Sosa Ricardo, D. Stafford, N. Tonon, M. Van De Klundert, F. Vazzoler, A. Ventura Barroso, R. Walsh, D. Walter, Q. Wang, Y. Wen, K. Wichmann, L. Wiens, C. Wissing, S. Wuchterl, Y. Yang, A. Zimermmane Castro Santos, R. Aggleton, A. Albrecht, S. Albrecht, M. Antonello, S. Bein, L. Benato, M. Bonanomi, P. Connor, K. De Leo, M. Eich, K. El Morabit, F. Feindt, A. Fröhlich, C. Garbers, E. Garutti, M. Hajheidari, J. Haller, A. Hinzmann, H. R. Jabusch, G. Kasieczka, R. Klanner, W. Korcari, T. Kramer, V. Kutzner, J. Lange, T. Lange, A. Lobanov, C. Matthies, A. Mehta, L. Moureaux, M. Mrowietz, A. Nigamova, Y. Nissan, A. Paasch, K. J. Pena Rodriguez, M. Rieger, O. Rieger, P. Schleper, M. Schröder, J. Schwandt, H. Stadie, G. Steinbrück, A. Tews, M. Wolf, J. Bechtel, S. Brommer, M. Burkart, E. Butz, R. Caspart, T. Chwalek, A. Dierlamm, A. Droll, N. Faltermann, M. Giffels, J. O. Gosewisch, A. Gottmann, F. Hartmann, C. Heidecker, M. Horzela, U. Husemann, P. Keicher, M. Klute, R. Koppenhöfer, S. Maier, S. Mitra, Th. Müller, M. Neukum, G. Quast, K. Rabbertz, J. Rauser, D. Savoiu, M. Schnepf, D. Seith, I. Shvetsov, H. J. Simonis, N. Trevisani, R. Ulrich, J. van der Linden, R. F. Von Cube, M. Wassmer, M. Weber, S. Wieland, R. Wolf, S. Wozniewski, S. Wunsch, G. Anagnostou, P. Assiouras, G. Daskalakis, A. Kyriakis, A. Stakia, M. Diamantopoulou, D. Karasavvas, P. Kontaxakis, A. Manousakis-Katsikakis, A. Panagiotou, I. Papavergou, N. Saoulidou, K. Theofilatos, E. Tziaferi, K. Vellidis, E. Vourliotis, G. Bakas, T. Chatzistavrou, K. Kousouris, I. Papakrivopoulos, G. Tsipolitis, A. Zacharopoulou, K. Adamidis, I. Bestintzanos, I. Evangelou, C. Foudas, P. Gianneios, C. Kamtsikis, P. Katsoulis, P. Kokkas, P. G. Kosmoglou Kioseoglou, N. Manthos, I. Papadopoulos, J. Strologas, M. Csanád, K. Farkas, M. M. A. Gadallah, S. Lökös, P. Major, K. Mandal, G. Pásztor, A. J. Rádl, O. Surányi, G. I. Veres, M. Bartók, G. Bencze, C. Hajdu, D. Horvath, F. Sikler, V. Veszpremi, N. Beni, S. Czellar, D. Fasanella, J. Karancsi, J. Molnar, Z. Szillasi, D. Teyssier, B. Ujvari, G. Zilizi, T. Csorgo, F. Nemes, T. Novak, J. Babbar, S. Bansal, S. B. Beri, V. Bhatnagar, G. Chaudhary, S. Chauhan, N. Dhingra, R. Gupta, A. Kaur, A. K. Virdi, H. Kaur, M. Kaur, S. Kumar, P. Kumari, M. Meena, K. Sandeep, T. Sheokand, J. B. Singh, A. Singla, A. Ahmed, A. Bhardwaj, B. C. Choudhary, M. Gola, S. Keshri, A. Kumar, M. Naimuddin, P. Priyanka, K. Ranjan, S. Saumya, A. Shah, S. Baradia, S. Barman, S. Bhattacharya, D. Bhowmik, S. Dutta, B. Gomber, M. Maity, P. Palit, P. K. Rout, G. Saha, B. Sahu, S. Sarkar, P. K. Behera, S. C. Behera, P. Kalbhor, J. R. Komaragiri, D. Kumar, A. Muhammad, L. Panwar, R. Pradhan, P. R. Pujahari, A. Sharma, A. K. Sikdar, P. C. Tiwari, S. Verma, K. Naskar, T. Aziz, I. Das, S. Dugad, M. Kumar, G. B. Mohanty, P. Suryadevara, S. Banerjee, R. Chudasama, M. Guchait, S. Karmakar, S. Kumar, G. Majumder, K. Mazumdar, S. Mukherjee, A. Thachayath, S. Bahinipati, A. K. Das, C. Kar, P. Mal, T. Mishra, V. K. Muraleedharan Nair Bindhu, A. Nayak, P. Saha, N. Sur, S. K. Swain, D. Vats, A. Alpana, S. Dube, B. Kansal, A. Laha, S. Pandey, A. Rastogi, S. Sharma, H. Bakhshiansohi, E. Khazaie, M. Zeinali, S. Chenarani, S. M. Etesami, M. Khakzad, M. Mohammadi Najafabadi, M. Felcini, M. Grunewald, M. Abbrescia, R. Aly, C. Aruta, A. Colaleo, D. Creanza, N. De Filippis, M. De Palma, A. Di Florio, W. Elmetenawee, F. Errico, L. Fiore, G. Iaselli, M. Ince, G. Maggi, M. Maggi, I. Margjeka, V. Mastrapasqua, S. My, S. Nuzzo, A. Pellecchia, A. Pompili, G. Pugliese, R. Radogna, D. Ramos, A. Ranieri, G. Selvaggi, L. Silvestris, F. M. Simone, Ü. Sözbilir, A. Stamerra, R. Venditti, P. Verwilligen, A. Zaza, G. Abbiendi, C. Battilana, D. Bonacorsi, L. Borgonovi, L. Brigliadori, R. Campanini, P. Capiluppi, A. Castro, F. R. Cavallo, M. Cuffiani, G. M. Dallavalle, T. Diotalevi, F. Fabbri, A. Fanfani, P. Giacomelli, L. Giommi, C. Grandi, L. Guiducci, S. Lo Meo, L. Lunerti, S. Marcellini, G. Masetti, F. L. Navarria, A. Perrotta, F. Primavera, A. M. Rossi, T. Rovelli, G. P. Siroli, S. Costa, A. Di Mattia, R. Potenza, A. Tricomi, C. Tuve, G. Barbagli, B. Camaiani, A. Cassese, R. Ceccarelli, V. Ciulli, C. Civinini, R. D’Alessandro, E. Focardi, G. Latino, P. Lenzi, M. Lizzo, M. Meschini, S. Paoletti, R. Seidita, G. Sguazzoni, L. Viliani, L. Benussi, S. Bianco, S. Meola, D. Piccolo, M. Bozzo, F. Ferro, R. Mulargia, E. Robutti, S. Tosi, A. Benaglia, G. Boldrini, F. Brivio, F. Cetorelli, F. De Guio, M. E. Dinardo, P. Dini, S. Gennai, A. Ghezzi, P. Govoni, L. Guzzi, M. T. Lucchini, M. Malberti, S. Malvezzi, A. Massironi, D. Menasce, L. Moroni, M. Paganoni, D. Pedrini, B. S. Pinolini, S. Ragazzi, N. Redaelli, T. Tabarelli de Fatis, D. Zuolo, S. Buontempo, F. Carnevali, N. Cavallo, A. De Iorio, F. Fabozzi, A. O. M. Iorio, L. Lista, P. Paolucci, B. Rossi, C. Sciacca, P. Azzi, N. Bacchetta, D. Bisello, P. Bortignon, A. Bragagnolo, R. Carlin, P. Checchia, T. Dorigo, F. Gasparini, U. Gasparini, G. Grosso, L. Layer, E. Lusiani, M. Margoni, A. T. Meneguzzo, J. Pazzini, P. Ronchese, R. Rossin, F. Simonetto, G. Strong, M. Tosi, H. Yarar, M. Zanetti, P. Zotto, A. Zucchetta, G. Zumerle, C. Aimè, A. Braghieri, S. Calzaferri, D. Fiorina, P. Montagna, V. Re, C. Riccardi, P. Salvini, I. Vai, P. Vitulo, P. Asenov, G. M. Bilei, D. Ciangottini, L. Fanò, M. Magherini, G. Mantovani, V. Mariani, M. Menichelli, F. Moscatelli, A. Piccinelli, M. Presilla, A. Rossi, A. Santocchia, D. Spiga, T. Tedeschi, P. Azzurri, G. Bagliesi, V. Bertacchi, R. Bhattacharya, L. Bianchini, T. Boccali, E. Bossini, D. Bruschini, R. Castaldi, M. A. Ciocci, V. D’Amante, R. Dell’Orso, M. R. Di Domenico, S. Donato, A. Giassi, F. Ligabue, E. Manca, G. Mandorli, D. Matos Figueiredo, A. Messineo, M. Musich, F. Palla, S. Parolia, G. Ramirez-Sanchez, A. Rizzi, G. Rolandi, S. Roy Chowdhury, A. Scribano, N. Shafiei, P. Spagnolo, R. Tenchini, G. Tonelli, N. Turini, A. Venturi, P. G. Verdini, P. Barria, M. Campana, F. Cavallari, D. Del Re, E. Di Marco, M. Diemoz, E. Longo, P. Meridiani, G. Organtini, F. Pandolfi, R. Paramatti, C. Quaranta, S. Rahatlou, C. Rovelli, F. Santanastasio, L. Soffi, R. Tramontano, N. Amapane, R. Arcidiacono, S. Argiro, M. Arneodo, N. Bartosik, R. Bellan, A. Bellora, J. Berenguer Antequera, C. Biino, N. Cartiglia, M. Costa, R. Covarelli, N. Demaria, M. Grippo, B. Kiani, F. Legger, C. Mariotti, S. Maselli, A. Mecca, E. Migliore, E. Monteil, M. Monteno, M. M. Obertino, G. Ortona, L. Pacher, N. Pastrone, M. Pelliccioni, M. Ruspa, K. Shchelina, F. Siviero, V. Sola, A. Solano, D. Soldi, A. Staiano, M. Tornago, D. Trocino, G. Umoret, A. Vagnerini, S. Belforte, V. Candelise, M. Casarsa, F. Cossutti, A. Da Rold, G. Della Ricca, G. Sorrentino, S. Dogra, C. Huh, B. Kim, D. H. Kim, G. N. Kim, J. Kim, J. Lee, S. W. Lee, C. S. Moon, Y. D. Oh, S. I. Pak, S. Sekmen, Y. C. Yang, H. Kim, D. H. Moon, E. Asilar, T. J. Kim, J. Park, S. Cho, S. Choi, S. Han, B. Hong, K. Lee, K. S. Lee, J. Lim, J. Park, S. K. Park, J. Yoo, J. Goh, H. S. Kim, Y. Kim, S. Lee, J. Almond, J. H. Bhyun, J. Choi, S. Jeon, W. Jun, J. S. Kim, J. Kim, S. Ko, H. Kwon, H. Lee, J. Lee, S. Lee, B. H. Oh, M. Oh, S. B. Oh, H. Seo, U. K. Yang, I. Yoon, W. Jang, D. Y. Kang, Y. Kang, D. Kim, S. Kim, B. Ko, J. S. H. Lee, Y. Lee, J. A. Merlin, I. C. Park, Y. Roh, M. S. Ryu, D. Song, I. J. Watson, S. Yang, S. Ha, H. D. Yoo, M. Choi, H. Lee, Y. Lee, I. Yu, T. Beyrouthy, Y. Maghrbi, K. Dreimanis, A. Gaile, A. Potrebko, T. Torims, V. Veckalns, M. Ambrozas, A. Carvalho Antunes De Oliveira, A. Juodagalvis, A. Rinkevicius, G. Tamulaitis, N. Bin Norjoharuddeen, S. Y. Hoh, I. Yusuff, Z. Zolkapli, J. F. Benitez, A. Castaneda Hernandez, H. A. Encinas Acosta, L. G. Gallegos Maríñez, M. León Coello, J. A. Murillo Quijada, A. Sehrawat, L. Valencia Palomo, G. Ayala, H. Castilla-Valdez, E. De La Cruz-Burelo, I. Heredia-De La Cruz, R. Lopez-Fernandez, C. A. Mondragon Herrera, D. A. Perez Navarro, A. Sánchez Hernández, C. Oropeza Barrera, F. Vazquez Valencia, I. Pedraza, H. A. Salazar Ibarguen, C. Uribe Estrada, I. Bubanja, J. Mijuskovic, N. Raicevic, A. Ahmad, M. I. Asghar, A. Awais, M. I. M. Awan, M. Gul, H. R. Hoorani, W. A. Khan, V. Avati, L. Grzanka, M. Malawski, H. Bialkowska, M. Bluj, B. Boimska, M. Górski, M. Kazana, M. Szleper, P. Zalewski, K. Bunkowski, K. Doroba, A. Kalinowski, M. Konecki, J. Krolikowski, M. Araujo, P. Bargassa, D. Bastos, A. Boletti, P. Faccioli, M. Gallinaro, J. Hollar, N. Leonardo, T. Niknejad, M. Pisano, J. Seixas, O. Toldaiev, J. Varela, P. Adzic, M. Dordevic, P. Milenovic, J. Milosevic, M. Aguilar-Benitez, J. Alcaraz Maestre, A. Álvarez Fernández, M. Barrio Luna, C. A. Carrillo Montoya, M. Cepeda, M. Cerrada, N. Colino, B. De La Cruz, A. Delgado Peris, Cristina F. Bedoya, D. Fernández Del Val, J. P. Fernández Ramos, J. Flix, M. C. Fouz, O. Gonzalez Lopez, S. Goy Lopez, J. M. Hernandez, M. I. Josa, J. León Holgado, D. Moran, C. Perez Dengra, A. Pérez-Calero Yzquierdo, J. Puerta Pelayo, D. D. Redondo Ferrero, I. Redondo, L. Romero, S. Sánchez Navas, J. Sastre, L. Urda Gómez, J. Vazquez Escobar, C. Willmott, J. F. de Trocóniz, B. Alvarez Gonzalez, J. Cuevas, J. Fernandez Menendez, S. Folgueras, I. Gonzalez Caballero, J. R. González Fernández, E. Palencia Cortezon, C. Ramón Álvarez, V. Rodríguez Bouza, A. Soto Rodríguez, A. Trapote, C. Vico Villalba, J. A. Brochero Cifuentes, I. J. Cabrillo, A. Calderon, J. Duarte Campderros, C. Fernandez Madrazo, M. Fernandez, A. García Alonso, G. Gomez, C. Lasaosa García, C. Martinez Rivero, P. Martinez Ruiz del Arbol, P. Matorras Cuevas, F. Matorras, J. Piedra Gomez, C. Prieels, A. Ruiz-Jimeno, L. Scodellaro, I. Vila, J. M. Vizan Garcia, D. D. C. Wickramarathna, B. Kailasapathy, M. K. Jayananda, D. U. J. Sonnadara, W. G. D. Dharmaratna, K. Liyanage, N. Perera, N. Wickramage, D. Abbaneo, J. Alimena, E. Auffray, G. Auzinger, J. Baechler, P. Baillon, D. Barney, J. Bendavid, M. Bianco, B. Bilin, A. Bocci, E. Brondolin, C. Caillol, T. Camporesi, G. Cerminara, N. Chernyavskaya, S. S. Chhibra, S. Choudhury, M. Cipriani, L. Cristella, D. d’Enterria, A. Dabrowski, A. David, A. De Roeck, M. M. Defranchis, M. Deile, M. Dobson, M. Dünser, N. Dupont, A. Elliott-Peisert, F. Fallavollita, A. Florent, L. Forthomme, G. Franzoni, W. Funk, S. Ghosh, S. Giani, D. Gigi, K. Gill, F. Glege, L. Gouskos, E. Govorkova, M. Haranko, J. Hegeman, V. Innocente, T. James, P. Janot, J. Kaspar, J. Kieseler, N. Kratochwil, S. Laurila, P. Lecoq, A. Lintuluoto, C. Lourenço, B. Maier, L. Malgeri, M. Mannelli, A. C. Marini, F. Meijers, S. Mersi, E. Meschi, F. Moortgat, M. Mulders, S. Orfanelli, L. Orsini, F. Pantaleo, E. Perez, M. Peruzzi, A. Petrilli, G. Petrucciani, A. Pfeiffer, M. Pierini, D. Piparo, M. Pitt, H. Qu, T. Quast, D. Rabady, A. Racz, G. Reales Gutiérrez, M. Rovere, H. Sakulin, J. Salfeld-Nebgen, S. Scarfi, M. Selvaggi, A. Sharma, P. Silva, P. Sphicas, A. G. Stahl Leiton, S. Summers, K. Tatar, V. R. Tavolaro, D. Treille, P. Tropea, A. Tsirou, J. Wanczyk, K. A. Wozniak, W. D. Zeuner, L. Caminada, A. Ebrahimi, W. Erdmann, R. Horisberger, Q. Ingram, H. C. Kaestli, D. Kotlinski, C. Lange, M. Missiroli, L. Noehte, T. Rohe, T. K. Aarrestad, K. Androsov, M. Backhaus, P. Berger, A. Calandri, K. Datta, A. De Cosa, G. Dissertori, M. Dittmar, M. Donegà, F. Eble, M. Galli, K. Gedia, F. Glessgen, T. A. Gómez Espinosa, C. Grab, D. Hits, W. Lustermann, A.-M. Lyon, R. A. Manzoni, L. Marchese, C. Martin Perez, A. Mascellani, M. T. Meinhard, F. Nessi-Tedaldi, J. Niedziela, F. Pauss, V. Perovic, S. Pigazzini, M. G. Ratti, M. Reichmann, C. Reissel, T. Reitenspiess, B. Ristic, F. Riti, D. Ruini, D. A. Sanz Becerra, J. Steggemann, D. Valsecchi, R. Wallny, C. Amsler, P. Bärtschi, C. Botta, D. Brzhechko, M. F. Canelli, K. Cormier, A. De Wit, R. Del Burgo, J. K. Heikkilä, M. Huwiler, W. Jin, A. Jofrehei, B. Kilminster, S. Leontsinis, S. P. Liechti, A. Macchiolo, P. Meiring, V. M. Mikuni, U. Molinatti, I. Neutelings, A. Reimers, P. Robmann, S. Sanchez Cruz, K. Schweiger, M. Senger, Y. Takahashi, C. Adloff, C. M. Kuo, W. Lin, S. S. Yu, L. Ceard, Y. Chao, K. F. Chen, P. S. Chen, H. Cheng, W.-S. Hou, Y. Y. Li, R.-S. Lu, E. Paganis, A. Psallidas, A. Steen, H. Y. Wu, E. Yazgan, P. R. Yu, C. Asawatangtrakuldee, N. Srimanobhas, D. Agyel, F. Boran, Z. S. Demiroglu, F. Dolek, I. Dumanoglu, E. Eskut, Y. Guler, E. Gurpinar Guler, C. Isik, O. Kara, A. Kayis Topaksu, U. Kiminsu, G. Onengut, K. Ozdemir, A. Polatoz, A. E. Simsek, B. Tali, U. G. Tok, S. Turkcapar, E. Uslan, I. S. Zorbakir, G. Karapinar, K. Ocalan, M. Yalvac, B. Akgun, I. O. Atakisi, E. Gülmez, M. Kaya, O. Kaya, Ö. Özçelik, S. Tekten, A. Cakir, K. Cankocak, Y. Komurcu, S. Sen, O. Aydilek, S. Cerci, B. Hacisahinoglu, I. Hos, B. Isildak, B. Kaynak, S. Ozkorucuklu, C. Simsek, D. Sunar Cerci, B. Grynyov, L. Levchuk, D. Anthony, E. Bhal, J. J. Brooke, A. Bundock, E. Clement, D. Cussans, H. Flacher, M. Glowacki, J. Goldstein, G. P. Heath, H. F. Heath, L. Kreczko, B. Krikler, S. Paramesvaran, S. Seif El Nasr-Storey, V. J. Smith, N. Stylianou, K. Walkingshaw Pass, R. White, A. H. Ball, K. W. Bell, A. Belyaev, C. Brew, R. M. Brown, D. J. A. Cockerill, C. Cooke, K. V. Ellis, K. Harder, S. Harper, M.-L. Holmberg, J. Linacre, K. Manolopoulos, D. M. Newbold, E. Olaiya, D. Petyt, T. Reis, G. Salvi, T. Schuh, C. H. Shepherd-Themistocleous, I. R. Tomalin, T. Williams, R. Bainbridge, P. Bloch, S. Bonomally, J. Borg, S. Breeze, C. E. Brown, O. Buchmuller, V. Cacchio, V. Cepaitis, G. S. Chahal, D. Colling, J. S. Dancu, P. Dauncey, G. Davies, J. Davies, M. Della Negra, S. Fayer, G. Fedi, G. Hall, M. H. Hassanshahi, A. Howard, G. Iles, J. Langford, L. Lyons, A.-M. Magnan, S. Malik, A. Martelli, M. Mieskolainen, D. G. Monk, J. Nash, M. Pesaresi, B. C. Radburn-Smith, D. M. Raymond, A. Richards, A. Rose, E. Scott, C. Seez, A. Shtipliyski, R. Shukla, A. Tapper, K. Uchida, G. P. Uttley, L. H. Vage, T. Virdee, M. Vojinovic, N. Wardle, S. N. Webb, D. Winterbottom, K. Coldham, J. E. Cole, A. Khan, P. Kyberd, I. D. Reid, L. Teodorescu, S. Abdullin, A. Brinkerhoff, B. Caraway, J. Dittmann, K. Hatakeyama, A. R. Kanuganti, B. McMaster, M. Saunders, S. Sawant, C. Sutantawibul, J. Wilson, R. Bartek, A. Dominguez, R. Uniyal, A. M. Vargas Hernandez, A. Buccilli, S. I. Cooper, D. Di Croce, S. V. Gleyzer, C. Henderson, C. U. Perez, P. Rumerio, C. West, A. Akpinar, A. Albert, D. Arcaro, C. Cosby, Z. Demiragli, C. Erice, E. Fontanesi, D. Gastler, S. May, J. Rohlf, K. Salyer, D. Sperka, D. Spitzbart, I. Suarez, A. Tsatsos, S. Yuan, G. Benelli, B. Burkle, X. Coubez, D. Cutts, M. Hadley, U. Heintz, J. M. Hogan, T. Kwon, G. Landsberg, K. T. Lau, D. Li, J. Luo, M. Narain, N. Pervan, S. Sagir, F. Simpson, E. Usai, W. Y. Wong, X. Yan, D. Yu, W. Zhang, J. Bonilla, C. Brainerd, R. Breedon, M. Calderon De La Barca Sanchez, M. Chertok, J. Conway, P. T. Cox, R. Erbacher, G. Haza, F. Jensen, O. Kukral, G. Mocellin, M. Mulhearn, D. Pellett, B. Regnery, D. Taylor, Y. Yao, F. Zhang, M. Bachtis, R. Cousins, A. Datta, D. Hamilton, J. Hauser, M. Ignatenko, M. A. Iqbal, T. Lam, W. A. Nash, S. Regnard, D. Saltzberg, B. Stone, V. Valuev, Y. Chen, R. Clare, J. W. Gary, M. Gordon, G. Hanson, G. Karapostoli, O. R. Long, N. Manganelli, W. Si, S. Wimpenny, J. G. Branson, P. Chang, S. Cittolin, S. Cooperstein, D. Diaz, J. Duarte, R. Gerosa, L. Giannini, J. Guiang, R. Kansal, V. Krutelyov, R. Lee, J. Letts, M. Masciovecchio, F. Mokhtar, M. Pieri, B. V. Sathia Narayanan, V. Sharma, M. Tadel, F. Würthwein, Y. Xiang, A. Yagil, N. Amin, C. Campagnari, M. Citron, G. Collura, A. Dorsett, V. Dutta, J. Incandela, M. Kilpatrick, J. Kim, A. J. Li, B. Marsh, P. Masterson, H. Mei, M. Oshiro, M. Quinnan, J. Richman, U. Sarica, R. Schmitz, F. Setti, J. Sheplock, P. Siddireddy, D. Stuart, S. Wang, A. Bornheim, O. Cerri, I. Dutta, J. M. Lawhorn, N. Lu, J. Mao, H. B. Newman, T. Q. Nguyen, M. Spiropulu, J. R. Vlimant, C. Wang, S. Xie, Z. Zhang, R. Y. Zhu, J. Alison, S. An, M. B. Andrews, P. Bryant, T. Ferguson, A. Harilal, C. Liu, T. Mudholkar, S. Murthy, M. Paulini, A. Roberts, A. Sanchez, W. Terrill, J. P. Cumalat, W. T. Ford, A. Hassani, G. Karathanasis, E. MacDonald, F. Marini, R. Patel, A. Perloff, C. Savard, N. Schonbeck, K. Stenson, K. A. Ulmer, S. R. Wagner, N. Zipper, J. Alexander, S. Bright-Thonney, X. Chen, D. J. Cranshaw, J. Fan, X. Fan, D. Gadkari, S. Hogan, J. Monroy, J. R. Patterson, D. Quach, J. Reichert, M. Reid, A. Ryd, J. Thom, P. Wittich, R. Zou, M. Albrow, M. Alyari, G. Apollinari, A. Apresyan, L. A. T. Bauerdick, D. Berry, J. Berryhill, P. C. Bhat, K. Burkett, J. N. Butler, A. Canepa, G. B. Cerati, H. W. K. Cheung, F. Chlebana, K. F. Di Petrillo, J. Dickinson, V. D. Elvira, Y. Feng, J. Freeman, A. Gandrakota, Z. Gecse, L. Gray, D. Green, S. Grünendahl, O. Gutsche, R. M. Harris, R. Heller, T. C. Herwig, J. Hirschauer, L. Horyn, B. Jayatilaka, S. Jindariani, M. Johnson, U. Joshi, T. Klijnsma, B. Klima, K. H. M. Kwok, S. Lammel, D. Lincoln, R. Lipton, T. Liu, C. Madrid, K. Maeshima, C. Mantilla, D. Mason, P. McBride, P. Merkel, S. Mrenna, S. Nahn, J. Ngadiuba, V. Papadimitriou, N. Pastika, K. Pedro, C. Pena, F. Ravera, A. Reinsvold Hall, L. Ristori, E. Sexton-Kennedy, N. Smith, A. Soha, L. Spiegel, J. Strait, L. Taylor, S. Tkaczyk, N. V. Tran, L. Uplegger, E. W. Vaandering, H. A. Weber, I. Zoi, P. Avery, D. Bourilkov, L. Cadamuro, V. Cherepanov, R. D. Field, D. Guerrero, M. Kim, E. Koenig, J. Konigsberg, A. Korytov, K. H. Lo, K. Matchev, N. Menendez, G. Mitselmakher, A. Muthirakalayil Madhu, N. Rawal, D. Rosenzweig, S. Rosenzweig, K. Shi, J. Wang, Z. Wu, T. Adams, A. Askew, R. Habibullah, V. Hagopian, R. Khurana, T. Kolberg, G. Martinez, H. Prosper, C. Schiber, O. Viazlo, R. Yohay, J. Zhang, M. M. Baarmand, S. Butalla, T. Elkafrawy, M. Hohlmann, R. Kumar Verma, D. Noonan, M. Rahmani, F. Yumiceva, M. R. Adams, H. Becerril Gonzalez, R. Cavanaugh, D. S. Lemos, S. Dittmer, O. Evdokimov, C. E. Gerber, D. J. Hofman, A. H. Merrit, C. Mills, G. Oh, T. Roy, S. Rudrabhatla, M. B. Tonjes, N. Varelas, X. Wang, Z. Ye, J. Yoo, M. Alhusseini, K. Dilsiz, L. Emediato, R. P. Gandrajula, G. Karaman, O. K. Köseyan, J.-P. Merlo, A. Mestvirishvili, J. Nachtman, O. Neogi, H. Ogul, Y. Onel, A. Penzo, C. Snyder, E. Tiras, O. Amram, B. Blumenfeld, L. Corcodilos, J. Davis, A. V. Gritsan, L. Kang, S. Kyriacou, P. Maksimovic, J. Roskes, S. Sekhar, M. Swartz, T. Á. Vámi, A. Abreu, L. F. Alcerro Alcerro, J. Anguiano, P. Baringer, A. Bean, Z. Flowers, T. Isidori, S. Khalil, J. King, G. Krintiras, M. Lazarovits, C. Le Mahieu, C. Lindsey, J. Marquez, N. Minafra, M. Murray, M. Nickel, C. Rogan, C. Royon, R. Salvatico, S. Sanders, E. Schmitz, C. Smith, Q. Wang, Z. Warner, J. Williams, G. Wilson, B. Allmond, S. Duric, R. Gujju Gurunadha, A. Ivanov, K. Kaadze, D. Kim, Y. Maravin, T. Mitchell, A. Modak, K. Nam, J. Natoli, D. Roy, F. Rebassoo, D. Wright, E. Adams, A. Baden, O. Baron, A. Belloni, A. Bethani, S. C. Eno, N. J. Hadley, S. Jabeen, R. G. Kellogg, T. Koeth, Y. Lai, S. Lascio, A. C. Mignerey, S. Nabili, C. Palmer, C. Papageorgakis, M. Seidel, L. Wang, K. Wong, D. Abercrombie, R. Bi, W. Busza, I. A. Cali, Y. Chen, M. D’Alfonso, J. Eysermans, C. Freer, G. Gomez-Ceballos, M. Goncharov, P. Harris, M. Hu, D. Kovalskyi, J. Krupa, Y.-J. Lee, K. Long, C. Mironov, C. Paus, D. Rankin, C. Roland, G. Roland, Z. Shi, G. S. F. Stephans, J. Wang, Z. Wang, B. Wyslouch, R. M. Chatterjee, B. Crossman, A. Evans, J. Hiltbrand, Sh. Jain, B. M. Joshi, C. Kapsiak, M. Krohn, Y. Kubota, J. Mans, M. Revering, R. Rusack, R. Saradhy, N. Schroeder, N. Strobbe, M. A. Wadud, L. M. Cremaldi, K. Bloom, M. Bryson, S. Chauhan, D. R. Claes, C. Fangmeier, L. Finco, F. Golf, C. Joo, I. Kravchenko, I. Reed, J. E. Siado, G. R. Snow, W. Tabb, A. Wightman, F. Yan, A. G. Zecchinelli, G. Agarwal, H. Bandyopadhyay, L. Hay, I. Iashvili, A. Kharchilava, C. McLean, M. Morris, D. Nguyen, J. Pekkanen, S. Rappoccio, A. Williams, G. Alverson, E. Barberis, Y. Haddad, Y. Han, A. Krishna, J. Li, J. Lidrych, G. Madigan, B. Marzocchi, D. M. Morse, V. Nguyen, T. Orimoto, A. Parker, L. Skinnari, A. Tishelman-Charny, T. Wamorkar, B. Wang, A. Wisecarver, D. Wood, S. Bhattacharya, J. Bueghly, Z. Chen, A. Gilbert, T. Gunter, K. A. Hahn, Y. Liu, N. Odell, M. H. Schmitt, M. Velasco, R. Band, R. Bucci, S. Castells, M. Cremonesi, A. Das, R. Goldouzian, M. Hildreth, K. Hurtado Anampa, C. Jessop, K. Lannon, J. Lawrence, N. Loukas, L. Lutton, J. Mariano, N. Marinelli, I. Mcalister, T. McCauley, C. Mcgrady, K. Mohrman, C. Moore, Y. Musienko, H. Nelson, R. Ruchti, A. Townsend, M. Wayne, H. Yockey, M. Zarucki, L. Zygala, B. Bylsma, M. Carrigan, L. S. Durkin, B. Francis, C. Hill, A. Lesauvage, M. Nunez Ornelas, K. Wei, B. L. Winer, B. R. Yates, F. M. Addesa, B. Bonham, P. Das, G. Dezoort, P. Elmer, A. Frankenthal, B. Greenberg, N. Haubrich, S. Higginbotham, A. Kalogeropoulos, G. Kopp, S. Kwan, D. Lange, D. Marlow, K. Mei, I. Ojalvo, J. Olsen, D. Stickland, C. Tully, S. Malik, S. Norberg, A. S. Bakshi, V. E. Barnes, R. Chawla, S. Das, L. Gutay, M. Jones, A. W. Jung, D. Kondratyev, A. M. Koshy, M. Liu, G. Negro, N. Neumeister, G. Paspalaki, S. Piperov, A. Purohit, J. F. Schulte, M. Stojanovic, J. Thieman, F. Wang, R. Xiao, W. Xie, J. Dolen, N. Parashar, D. Acosta, A. Baty, T. Carnahan, M. Decaro, S. Dildick, K. M. Ecklund, P. J. Fernández Manteca, S. Freed, P. Gardner, F. J. M. Geurts, A. Kumar, W. Li, B. P. Padley, R. Redjimi, J. Rotter, W. Shi, S. Yang, E. Yigitbasi, L. Zhang, Y. Zhang, X. Zuo, A. Bodek, P. de Barbaro, R. Demina, J. L. Dulemba, C. Fallon, T. Ferbel, M. Galanti, A. Garcia-Bellido, O. Hindrichs, A. Khukhunaishvili, E. Ranken, R. Taus, G. P. Van Onsem, K. Goulianos, B. Chiarito, J. P. Chou, Y. Gershtein, E. Halkiadakis, A. Hart, M. Heindl, O. Karacheban, I. Laflotte, A. Lath, R. Montalvo, K. Nash, M. Osherson, S. Salur, S. Schnetzer, S. Somalwar, R. Stone, S. A. Thayil, S. Thomas, H. Wang, H. Acharya, A. G. Delannoy, S. Fiorendi, T. Holmes, E. Nibigira, S. Spanier, O. Bouhali, M. Dalchenko, A. Delgado, R. Eusebi, J. Gilmore, T. Huang, T. Kamon, H. Kim, S. Luo, S. Malhotra, R. Mueller, D. Overton, D. Rathjens, A. Safonov, N. Akchurin, J. Damgov, V. Hegde, K. Lamichhane, S. W. Lee, T. Mengke, S. Muthumuni, T. Peltola, I. Volobouev, Z. Wang, A. Whitbeck, E. Appelt, S. Greene, A. Gurrola, W. Johns, A. Melo, F. Romeo, P. Sheldon, S. Tuo, J. Velkovska, J. Viinikainen, B. Cardwell, B. Cox, G. Cummings, J. Hakala, R. Hirosky, M. Joyce, A. Ledovskoy, A. Li, C. Neu, C. E. Perez Lara, B. Tannenwald, P. E. Karchin, N. Poudyal, S. Banerjee, K. Black, T. Bose, S. Dasu, I. De Bruyn, P. Everaerts, C. Galloni, H. He, M. Herndon, A. Herve, C. K. Koraka, A. Lanaro, A. Loeliger, R. Loveless, J. Madhusudanan Sreekala, A. Mallampalli, A. Mohammadi, S. Mondal, G. Parida, D. Pinna, A. Savin, V. Shang, V. Sharma, W. H. Smith, D. Teague, H. F. Tsoi, W. Vetens, S. Afanasiev, V. Andreev, Yu. Andreev, T. Aushev, M. Azarkin, A. Babaev, A. Belyaev, V. Blinov, E. Boos, V. Borshch, D. Budkouski, V. Chekhovsky, R. Chistov, A. Dermenev, T. Dimova, I. Dremin, M. Dubinin, L. Dudko, V. Epshteyn, A. Ershov, G. Gavrilov, V. Gavrilov, S. Gninenko, V. Golovtcov, N. Golubev, I. Golutvin, I. Gorbunov, A. Gribushin, Y. Ivanov, V. Ivanchenko, V. Kachanov, L. Kardapoltsev, V. Karjavine, A. Karneyeu, V. Kim, M. Kirakosyan, D. Kirpichnikov, M. Kirsanov, V. Klyukhin, O. Kodolova, D. Konstantinov, V. Korenkov, A. Kozyrev, N. Krasnikov, E. Kuznetsova, A. Lanev, P. Levchenko, A. Litomin, N. Lychkovskaya, V. Makarenko, A. Malakhov, V. Matveev, V. Murzin, A. Nikitenko, S. Obraztsov, V. Okhotnikov, A. Oskin, I. Ovtin, V. Palichik, P. Parygin, V. Perelygin, S. Petrushanko, G. Pivovarov, S. Polikarpov, V. Popov, E. Popova, O. Radchenko, M. Savina, V. Savrin, D. Selivanova, V. Shalaev, S. Shmatov, S. Shulha, Y. Skovpen, S. Slabospitskii, V. Smirnov, A. Snigirev, D. Sosnov, A. Stepennov, V. Sulimov, E. Tcherniaev, A. Terkulov, O. Teryaev, I. Tlisova, M. Toms, A. Toropin, L. Uvarov, A. Uzunian, E. Vlasov, A. Vorobyev, N. Voytishin, B. S. Yuldashev, A. Zarubin, I. Zhizhin, A. Zhokin

**Affiliations:** 1 Yerevan Physics Institute, Yerevan, Armenia; 2 Institut für Hochenergiephysik, Vienna, Austria; 3 Universiteit Antwerpen, Antwerpen, Belgium; 4 Vrije Universiteit Brussel, Brussel, Belgium; 5 Université Libre de Bruxelles, Bruxelles, Belgium; 6 Ghent University, Ghent, Belgium; 7 Université Catholique de Louvain, Louvain-la-Neuve, Belgium; 8 Centro Brasileiro de Pesquisas Fisicas, Rio de Janeiro, Brazil; 9 Universidade do Estado do Rio de Janeiro, Rio de Janeiro, Brazil; 10 Universidade Estadual Paulista, Universidade Federal do ABC, São Paulo, Brazil; 11 Institute for Nuclear Research and Nuclear Energy, Bulgarian Academy of Sciences, Sofia, Bulgaria; 12 University of Sofia, Sofia, Bulgaria; 13 Beihang University, Beijing, China; 14 Department of Physics, Tsinghua University, Beijing, China; 15 Institute of High Energy Physics, Beijing, China; 16 State Key Laboratory of Nuclear Physics and Technology, Peking University, Beijing, China; 17 Sun Yat-Sen University, Guangzhou, China; 18 Institute of Modern Physics and Key Laboratory of Nuclear Physics and Ion-beam Application (MOE) -Fudan University, Shanghai, China; 19 Zhejiang University, Hangzhou, Zhejiang, China; 20 Universidad de Los Andes, Bogota, Colombia; 21 Universidad de Antioquia, Medellin, Colombia; 22 University of Split, Faculty of Electrical Engineering, Mechanical Engineering and Naval Architecture, Split, Croatia; 23 University of Split, Faculty of Science, Split, Croatia; 24 Institute Rudjer Boskovic, Zagreb, Croatia; 25 University of Cyprus, Nicosia, Cyprus; 26 Charles University, Prague, Czech Republic; 27 Escuela Politecnica Nacional, Quito, Ecuador; 28 Universidad San Francisco de Quito, Quito, Ecuador; 29 Academy of Scientific Research and Technology of the Arab Republic of Egypt, Egyptian Network of High EnergyPhysics, Cairo, Egypt; 30 Center for High Energy Physics (CHEP-FU), Fayoum University, El-Fayoum, Egypt; 31 National Institute of Chemical Physics and Biophysics, Tallinn, Estonia; 32 Department of Physics, University of Helsinki, Helsinki, Finland; 33 Helsinki Institute of Physics, Helsinki, Finland; 34 Lappeenranta-Lahti University of Technology, Lappeenranta, Finland; 35 IRFU, CEA, Université Paris-Saclay, Gif-sur-Yvette, France; 36 Laboratoire Leprince-Ringuet, CNRS/IN2P3, Ecole Polytechnique, Institut Polytechnique de Paris, Palaiseau, France; 37 Université de Strasbourg, CNRS, IPHC UMR 7178, Strasbourg, France; 38 Institut de Physique des 2 Infinis de Lyon (IP2I), Villeurbanne, France; 39 Georgian Technical University, Tbilisi, Georgia; 40 RWTH Aachen University, I. Physikalisches Institut, Aachen, Germany; 41 RWTH Aachen University, III. Physikalisches Institut A, Aachen, Germany; 42 RWTH Aachen University, III. Physikalisches Institut B, Aachen, Germany; 43 Deutsches Elektronen-Synchrotron, Hamburg, Germany; 44 University of Hamburg, Hamburg, Germany; 45 Karlsruher Institut fuer Technologie, Karlsruhe, Germany; 46 Institute of Nuclear and Particle Physics (INPP), NCSR Demokritos, Aghia Paraskevi, Greece; 47 National and Kapodistrian University of Athens, Athens, Greece; 48 National Technical University of Athens, Athens, Greece; 49 University of Ioánnina, Ioánnina, Greece; 50 MTA-ELTE Lendület CMS Particle and Nuclear Physics Group, Eötvös Loránd University, Budapest, Hungary; 51 Wigner Research Centre for Physics, Budapest, Hungary; 52 Institute of Nuclear Research ATOMKI, Debrecen, Hungary; 53 Institute of Physics, University of Debrecen, Debrecen, Hungary; 54 Karoly Robert Campus, MATE Institute of Technology, Gyongyos, Hungary; 55 Panjab University, Chandigarh, India; 56 University of Delhi, Delhi, India; 57 Saha Institute of Nuclear Physics, HBNI, Kolkata, India; 58 Indian Institute of Technology Madras, Madras, India; 59 Bhabha Atomic Research Centre, Mumbai, India; 60 Tata Institute of Fundamental Research-A, Mumbai, India; 61 Tata Institute of Fundamental Research-B, Mumbai, India; 62 National Institute of Science Education and Research, An OCC of Homi Bhabha National Institute, Bhubaneswar, Odisha India; 63 Indian Institute of Science Education and Research (IISER), Pune, India; 64 Isfahan University of Technology, Isfahan, Iran; 65 Institute for Research in Fundamental Sciences (IPM), Tehran, Iran; 66 University College Dublin, Dublin, Ireland; 67 INFN Sezione di Bari, Bari, Italy; 68 Università di Bari, Bari, Italy; 69 Politecnico di Bari, Bari, Italy; 70 INFN Sezione di Bologna, Bologna, Italy; 71 Università di Bologna, Bologna, Italy; 72 INFN Sezione di Catania, Catania, Italy; 73 Università di Catania, Catania, Italy; 74 INFN Sezione di Firenze, Firenze, Italy; 75 Università di Firenze, Firenze, Italy; 76 INFN Laboratori Nazionali di Frascati, Frascati, Italy; 77 INFN Sezione di Genova, Genova, Italy; 78 Università di Genova, Genova, Italy; 79 INFN Sezione di Milano-Bicocca, Milano, Italy; 80 Università di Milano-Bicocca, Milano, Italy; 81 INFN Sezione di Napoli, Napoli, Italy; 82 Università di Napoli ’Federico II’, Napoli, Italy; 83 Università della Basilicata, Potenza, Italy; 84 Università G. Marconi, Roma, Italy; 85 INFN Sezione di Padova, Padova, Italy; 86 Università di Padova, Padova, Italy; 87 Università di Trento, Trento, Italy; 88 INFN Sezione di Pavia, Pavia, Italy; 89 Università di Pavia, Pavia, Italy; 90 INFN Sezione di Perugia, Perugia, Italy; 91 Università di Perugia, Perugia, Italy; 92 INFN Sezione di Pisa, Pisa, Italy; 93 Scuola Normale Superiore di Pisa, Pisa, Italy; 94 Università di Pisa, Pisa, Italy; 95 Università di Siena, Siena, Italy; 96 INFN Sezione di Roma, Roma, Italy; 97 Sapienza Università di Roma, Roma, Italy; 98 INFN Sezione di Torino, Torino, Italy; 99 Università di Torino, Torino, Italy; 100 Università del Piemonte Orientale, Novara, Italy; 101 INFN Sezione di Trieste, Trieste, Italy; 102 Università di Trieste, Trieste, Italy; 103 Kyungpook National University, Daegu, Korea; 104 Chonnam National University, Institute for Universe and Elementary Particles, Kwangju, Korea; 105 Hanyang University, Seoul, Korea; 106 Korea University, Seoul, Korea; 107 Kyung Hee University, Department of Physics, Seoul, Korea; 108 Sejong University, Seoul, Korea; 109 Seoul National University, Seoul, Korea; 110 University of Seoul, Seoul, Korea; 111 Yonsei University, Department of Physics, Seoul, Korea; 112 Sungkyunkwan University, Suwon, Korea; 113 College of Engineering and Technology, American University of the Middle East (AUM), Dasman, Kuwait; 114 Riga Technical University, Riga, Latvia; 115 Vilnius University, Vilnius, Lithuania; 116 National Centre for Particle Physics, Universiti Malaya, Kuala Lumpur, Malaysia; 117 Universidad de Sonora (UNISON), Hermosillo, Mexico; 118 Centro de Investigacion y de Estudios Avanzados del IPN, Mexico City, Mexico; 119 Universidad Iberoamericana, Mexico City, Mexico; 120 Benemerita Universidad Autonoma de Puebla, Puebla, Mexico; 121 University of Montenegro, Podgorica, Montenegro; 122 National Centre for Physics, Quaid-I-Azam University, Islamabad, Pakistan; 123 AGH University of Science and Technology Faculty of Computer Science, Electronics and Telecommunications, Krakow, Poland; 124 National Centre for Nuclear Research, Swierk, Poland; 125 Institute of Experimental Physics, Faculty of Physics, University of Warsaw, Warsaw, Poland; 126 Laboratório de Instrumentação e Física Experimental de Partículas, Lisboa, Portugal; 127 VINCA Institute of Nuclear Sciences, University of Belgrade, Belgrade, Serbia; 128 Centro de Investigaciones Energéticas Medioambientales y Tecnológicas (CIEMAT), Madrid, Spain; 129 Universidad Autónoma de Madrid, Madrid, Spain; 130 Universidad de Oviedo, Instituto Universitario de Ciencias y Tecnologías Espaciales de Asturias (ICTEA), Oviedo, Spain; 131 Instituto de Física de Cantabria (IFCA), CSIC-Universidad de Cantabria, Santander, Spain; 132 University of Colombo, Colombo, Sri Lanka; 133 University of Ruhuna, Department of Physics, Matara, Sri Lanka; 134 CERN, European Organization for Nuclear Research, Geneva, Switzerland; 135 Paul Scherrer Institut, Villigen, Switzerland; 136 ETH Zurich -Institute for Particle Physics and Astrophysics (IPA), Zurich, Switzerland; 137 Universität Zürich, Zurich, Switzerland; 138 National Central University, Chung-Li, Taiwan; 139 National Taiwan University (NTU), Taipei, Taiwan; 140 Chulalongkorn University, Faculty of Science, Department of Physics, Bangkok, Thailand; 141 ukurovaÇ University, Physics Department, Science and Art Faculty, Adana, Turkey; 142 Middle East Technical University, Physics Department, Ankara, Turkey; 143 Bogazici University, Istanbul, Turkey; 144 Istanbul Technical University, Istanbul, Turkey; 145 Istanbul University, Istanbul, Turkey; 146 Institute for Scintillation Materials of National Academy of Science of Ukraine, Kharkiv, Ukraine; 147 National Science Centre, Kharkiv Institute of Physics and Technology, Kharkiv, Ukraine; 148 University of Bristol, Bristol, United Kingdom; 149 Rutherford Appleton Laboratory, Didcot, United Kingdom; 150 Imperial College, London, United Kingdom; 151 Brunel University, Uxbridge, United Kingdom; 152 Baylor University, Waco, Texas USA; 153 Catholic University of America, Washington, DC USA; 154 The University of Alabama, Tuscaloosa, Alabama USA; 155 Boston University, Boston, Massachusetts, USA; 156 Brown University, Providence, Rhode Island USA; 157 University of California, Davis, Davis, California USA; 158 University of California, Los Angeles, California USA; 159 University of California, Riverside, Riverside, California USA; 160 University of California, San Diego, La Jolla, California, USA; 161 University of California, Santa Barbara -Department of Physics, Santa Barbara, California, USA; 162 California Institute of Technology, Pasadena, California, USA; 163 Carnegie Mellon University, Pittsburgh, Pennsylvania USA; 164 University of Colorado Boulder, Boulder, Colorado, USA; 165 Cornell University, Ithaca, New York, USA; 166 Fermi National Accelerator Laboratory, Batavia, Illinois USA; 167 California Institute of Technology, Pasadena, California USA; 168 University of Florida, Gainesville, Florida USA; 169 Florida State University, Tallahassee, Florida USA; 170 Florida Institute of Technology, Melbourne, Florida USA; 171 University of Illinois at Chicago (UIC), Chicago, Illinois USA; 172 The University of Iowa, Iowa City, Iowa USA; 173 Johns Hopkins University, Baltimore, Maryland USA; 174 The University of Kansas, Lawrence, Kansas USA; 175 Kansas State University, Manhattan, Kansas USA; 176 Lawrence Livermore National Laboratory, Livermore, California USA; 177 University of Maryland, College Park, Maryland USA; 178 Massachusetts Institute of Technology, Cambridge, Massachusetts USA; 179 University of Minnesota, Minneapolis, Minnesota USA; 180 University of Mississippi, Oxford, Mississippi USA; 181 University of Nebraska-Lincoln, Lincoln, Nebraska USA; 182 State University of New York at Buffalo, Buffalo, New York USA; 183 Northeastern University, Boston, Massachusetts USA; 184 Northwestern University, Evanston, Illinois USA; 185 University of Notre Dame, Notre Dame, Indiana, USA; 186 The Ohio State University, Columbus, Ohio USA; 187 Princeton University, Princeton, New Jersey USA; 188 University of Puerto Rico, Mayaguez, Puerto Rico USA; 189 Purdue University, West Lafayette, Indiana, USA; 190 Purdue University Northwest, Hammond, Indiana, USA; 191 Rice University, Houston, Texas USA; 192 University of Rochester, Rochester, New York, USA; 193 The Rockefeller University, New York, New York USA; 194 Rutgers, The State University of New Jersey, Piscataway, New Jersey USA; 195 University of Tennessee, Knoxville, Tennessee USA; 196 Texas A&M University, College Station, Texas USA; 197 Texas Tech University, Lubbock, Texas USA; 198 Vanderbilt University, Nashville, Tennessee USA; 199 University of Virginia, Charlottesville, Virginia USA; 200 Wayne State University, Detroit, Michigan USA; 201 University of Wisconsin -Madison, Madison, Wisconsin USA; 202 Present Address: Istanbul University, Istanbul, Turkey; 203 Yerevan Physics Institute, Yerevan, Armenia; 204 Present Address: University of Florida, Gainesville, Florida USA; 205 Imperial College, London, United Kingdom; 206 Present Address: University of Rochester, Rochester, New York USA; 207 Present Address: University of Cyprus, Nicosia, Cyprus; 208 Present Address: Baylor University, Waco, Texas USA; 209 Present Address: INFN Sezione di Torino, Università di Torino, Torino, Italy; 210 Università del Piemonte Orientale., Novara, Italy; 211 Institute of Nuclear Physics of the Uzbekistan Academy of Sciences, Tashkent, Uzbekistan; 212 TU Wien, Vienna, Austria; 213 Institute of Basic and Applied Sciences, Faculty of Engineering, Arab Academy for Science, Technology and Maritime Transport, Alexandria, Egypt; 214 Université Libre de Bruxelles, Bruxelles, Belgium; 215 Universidade Estadual de Campinas, Campinas, Brazil; 216 Federal University of Rio Grande do Sul, Porto Alegre, Brazil; 217 UFMS, Nova Andradina, Brazil; 218 The University of the State of Amazonas, Manaus, Brazil; 219 University of Chinese Academy of Sciences, Beijing, China; 220 Nanjing Normal University Department of Physics, Nanjing, China; 221 Present Address: The University of Iowa, Iowa City, Iowa USA; 222 University of Chinese Academy of Sciences., Beijing, China; 223 Helwan University, Cairo, Egypt; 224 Present Address: Zewail City of Science and Technology, Zewail, Egypt; 225 British University in Egypt, Cairo, Egypt; 226 Ain Shams University, Cairo, Egypt; 227 Purdue University, West Lafayette, Indiana USA; 228 Université de Haute Alsace, Mulhouse, France; 229 Department of Physics, Tsinghua University, Beijing, China; 230 Tbilisi State University, Tbilisi, Georgia; 231 Erzincan Binali Yildirim University, Erzincan, Turkey; 232 CERN, European Organization for Nuclear Research, Geneva, Switzerland; 233 University of Hamburg, Hamburg, Germany; 234 RWTH Aachen University, III. Physikalisches Institut A, Aachen, Germany; 235 Isfahan University of Technology, Isfahan, Iran; 236 Brandenburg University of Technology, Cottbus, Germany; 237 Forschungszentrum Jülich, Juelich, Germany; 238 Physics Department, Faculty of Science, Assiut University, Assiut, Egypt; 239 Karoly Robert Campus, MATE Institute of Technology, Gyongyos, Hungary; 240 Wigner Research Centre for Physics, Budapest, Hungary; 241 Institute of Physics, University of Debrecen, Debrecen, Hungary; 242 Institute of Nuclear Research ATOMKI, Debrecen, Hungary; 243 Present Address: Universitatea Babes-Bolyai -Facultatea de Fizica, Cluj-Napoca, Romania; 244 Faculty of Informatics, University of Debrecen, Debrecen, Hungary; 245 Punjab Agricultural University, Ludhiana, India; 246 UPES -University of Petroleum and Energy Studies, Dehradun, India; 247 University of Visva-Bharati, Santiniketan, India; 248 University of Hyderabad, Hyderabad, India; 249 Indian Institute of Science (IISc), Bangalore, India; 250 Indian Institute of Technology (IIT), Mumbai, India; 251 IIT Bhubaneswar, Bhubaneswar, India; 252 Institute of Physics, Bhubaneswar, India; 253 Deutsches Elektronen-Synchrotron, Hamburg, Germany; 254 Department of Physics, Isfahan University of Technology, Isfahan, Iran; 255 Sharif University of Technology, Tehran, Iran; 256 Department of Physics, University of Science and Technology of Mazandaran, Behshahr, Iran; 257 Italian National Agency for New Technologies, Energy and Sustainable Economic Development, Bologna, Italy; 258 Centro Siciliano di Fisica Nucleare e di Struttura Della Materia, Catania, Italy; 259 Scuola Superiore Meridionale, Università di Napoli ’Federico II’, Napoli, Italy; 260 Fermi National Accelerator Laboratory, Batavia, Illinois USA; 261 Università di Napoli ’Federico II’, Napoli, Italy; 262 Consiglio Nazionale delle Ricerche -Istituto Officina dei Materiali, Perugia, Italy; 263 Riga Technical University, Riga, Latvia; 264 Department of Applied Physics, Faculty of Science and Technology, Universiti Kebangsaan Malaysia, Bangi, Malaysia; 265 Consejo Nacional de Ciencia y Tecnología, Mexico City, Mexico; 266 IRFU, CEA, Université Paris-Saclay, Gif-sur-Yvette, France; 267 Faculty of Physics, University of Belgrade, Belgrade, Serbia; 268 Trincomalee Campus, Eastern University, Sri Lanka, Nilaveli, Sri Lanka; 269 INFN Sezione di Pavia, Università di Pavia, Pavia, Italy; 270 National and Kapodistrian University of Athens, Athens, Greece; 271 Ecole Polytechnique Fédérale Lausanne, Lausanne, Switzerland; 272 Universität Zürich, Zurich, Switzerland; 273 Stefan Meyer Institute for Subatomic Physics, Vienna, Austria; 274 Laboratoire d’Annecy-le-Vieux de Physique des Particules, IN2P3-CNRS, Annecy-le-Vieux, France; 275 Near East University, Research Center of Experimental Health Science, Nicosia, Turkey; 276 Konya Technical University, Konya, Turkey; 277 Piri Reis University, Istanbul, Turkey; 278 Adiyaman University, Adiyaman, Turkey; 279 Necmettin Erbakan University, Konya, Turkey; 280 Bozok Universitetesi Rektörlügü, Yozgat, Turkey; 281 Marmara University, Istanbul, Turkey; 282 Milli Savunma University, Istanbul, Turkey; 283 Kafkas University, Kars, Turkey; 284 Hacettepe University, Ankara, Turkey; 285 Istanbul University -Cerrahpasa, Faculty of Engineering, Istanbul, Turkey; 286 Ozyegin University, Istanbul, Turkey; 287 Vrije Universiteit Brussel, Brussel, Belgium; 288 School of Physics and Astronomy, University of Southampton, Southampton, United Kingdom; 289 University of Bristol, Bristol, United Kingdom; 290 IPPP Durham University, Durham, United Kingdom; 291 Monash University, Faculty of Science, Clayton, Australia; 292 Università di Torino, Torino, Italy; 293 Bethel University, St. Paul, Minnesota, USA; 294 Karamanoğlu Mehmetbey University, Karaman, Turkey; 295 University of Science and Technology of China, Hefei, China; 296 United States Naval Academy, Annapolis, Maryland, USA; 297 Bingol University, Bingol, Turkey; 298 Georgian Technical University, Tbilisi, Georgia; 299 Sinop University, Sinop, Turkey; 300 Erciyes University, Kayseri, Turkey; 301 Institute of Modern Physics and Key Laboratory of Nuclear Physics and Ion-beam Application (MOE) -Fudan University, Shanghai, China; 302 Texas A&M University at Qatar, Doha, Qatar; 303 Kyungpook National University, Daegu, Korea

**Keywords:** Experimental particle physics

Correction to: *Nature* 10.1038/s41586-022-04892-x Published online 4 July 2022

In the version of this article initially published, CMS Collaboration author names, affiliations and acknowledgements were omitted and have now been included in the HTML and PDF versions of the article.

